# EHD1 promotes CP110 ubiquitination by centriolar satellite delivery of HERC2 to the mother centriole

**DOI:** 10.15252/embr.202256317

**Published:** 2023-04-19

**Authors:** Shuwei Xie, Naava Naslavsky, Steve Caplan

**Affiliations:** ^1^ Department of Biochemistry and Molecular Biology University of Nebraska Medical Center Omaha NE USA; ^2^ Fred and Pamela Buffett Cancer Center University of Nebraska Medical Center Omaha NE USA

**Keywords:** centriolar satellites, CP110, EHD1, HERC2, ubiquitination, Cell Adhesion, Polarity & Cytoskeleton, Membranes & Trafficking, Post-translational Modifications & Proteolysis

## Abstract

Primary cilia are sensory organelles that coordinate diverse signaling pathways, controlling development and homeostasis. Progression beyond the early steps of ciliogenesis requires the removal of a distal end protein, CP110, from the mother centriole, a process mediated by Eps15 Homology Domain protein 1 (EHD1). We show that EHD1 regulates CP110 ubiquitination during ciliogenesis, and identify two E3 ubiquitin ligases, HECT domain and RCC1‐like domain 2 (HERC2) and mindbomb homolog 1 (MIB1), that interact with and ubiquitinate CP110. We determined that HERC2 is required for ciliogenesis and localizes to centriolar satellites, which are peripheral aggregates of centriolar proteins known to regulate ciliogenesis. We reveal a role for EHD1 in the transport of centriolar satellites and HERC2 to the mother centriole during ciliogenesis. Taken together, our work showcases a mechanism whereby EHD1 controls centriolar satellite movement to the mother centriole, thus delivering the E3 ubiquitin ligase HERC2 to promote CP110 ubiquitination and degradation.

## Graphical abstract

Primary cilia are sensory organelles that coordinate diverse signaling pathways, controlling development and homeostasis. The endocytic regulatory protein, EHD1, facilitates centriolar satellite movement to the mother centriole leading to ubiquitination and degradation of the capping protein, CP110, thereby promoting primary ciliogenesis.

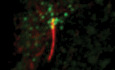

## Introduction

Primary cilia are evolutionarily conserved microtubule‐based structures that extend from the surface of the plasma membrane of most mammalian cells. Unlike the motile cilia that function in fluid movement in airway epithelial and other cells, the primary cilium is immotile and serves as a signaling hub that controls development and homeostasis. Indeed, impaired primary ciliogenesis can lead to multiple types of developmental disorders known as ciliopathies, including Bardet–Biedl syndrome (Novas *et al*, 2015), Joubert syndrome (Brancati *et al*, 2010), polycystic kidney disease (Ghata & Cowley Jr., 2017), retinal degeneration and a variety of other maladies (Fliegauf *et al*, 2007).

In the intracellular ciliogenesis pathway common to most fibroblasts and retinal pigmented epithelial (RPE‐1) cells (Sorokin, 1962), the generation of the primary cilium is induced by environmental cues that lead to the formation of a microtubule‐based axoneme that extends from the older of the two centrioles, the mother centriole (m‐centriole), which forms the basal body (Gerdes *et al*, 2009). One of the early events in generation of the primary cilium is the docking of Myosin‐Va‐containing precilliary vesicles on the distal appendages of the m‐centriole, suggesting regulation of ciliogenesis by the endocytic pathways (Wu *et al*, 2018). Indeed, ciliogenesis is a multistep process that is controlled by a variety of endocytic regulatory proteins. For example, the Eps15 Homology Domain protein, EHD1, is recruited to the centrosome by its interaction partner MICAL‐L1, which in turn is anchored to the centrosome by interactions with γ‐tubulin (Lu *et al*, 2015; Xie *et al*, 2019; Jones *et al*, 2022). Through its EH domain, EHD1 binds to the SNARE protein SNAP29 (Rotem‐Yehudar *et al*, 2001; Xu *et al*, 2004). EHD1 has also been implicated in the removal of CP110, a crucial negative regulator of ciliogenesis, from the m‐centriole to facilitate fusion of distal appendage vesicles and generation of the ciliary vesicle (CV; Lu *et al*, 2015). Additional endocytic regulators are key to the next steps of ciliogenesis, and a “RAB cascade” takes place in which ARL13b and RAB11 recruit an effector known as RABIN8 to the ciliary membrane, leading to the activation of RAB8 and driving the later stages of ciliogenesis (Knodler *et al*, 2010).

CP110 is one of the major negative regulators of primary ciliogenesis, and along with its interaction partner, CEP97, must be removed from the m‐centriole prior to extension of the axoneme and ciliary growth. CP110 is also key to centrosome biology and its phosphorylation by cyclin‐dependent kinases promotes centrosome duplication (Chen *et al*, 2002). In addition to micro RNAs (Cao *et al*, 2012; Walentek *et al*, 2016), a variety of proteins have been proposed to interact with and/or regulate CP110 removal from the m‐centriole, including KIF24 (Kobayashi *et al*, 2011), CEP104 (Jiang *et al*, 2012), Talpid3 (Kobayashi *et al*, 2014), CEP290 (Kobayashi *et al*, 2014), CEP164 (Cajanek & Nigg, 2014), Centrin2 (Prosser & Morrison, 2015), M‐Phase Phosphoprotein 9 (Huang *et al*, 2018), Centrobin (Ogungbenro *et al*, 2018), Tau‐tubulin kinase‐2‐dependent phosphorylation of CEP83 (Lo *et al*, 2019), CEP78 (Goncalves *et al*, 2021), NUDCL2 (Liu *et al*, 2021), linear ubiquitin chain assembly complex (LUBAC; Shen *et al*, 2022), and ENKD1 (Song *et al*, 2022). While the majority of these proteins are thought to be involved in the removal and/or degradation of CP110, the precise mechanisms of CP110 removal from the m‐centriole and its proteolytic degradation remain poorly understood.

## Results

**Figure 1:**
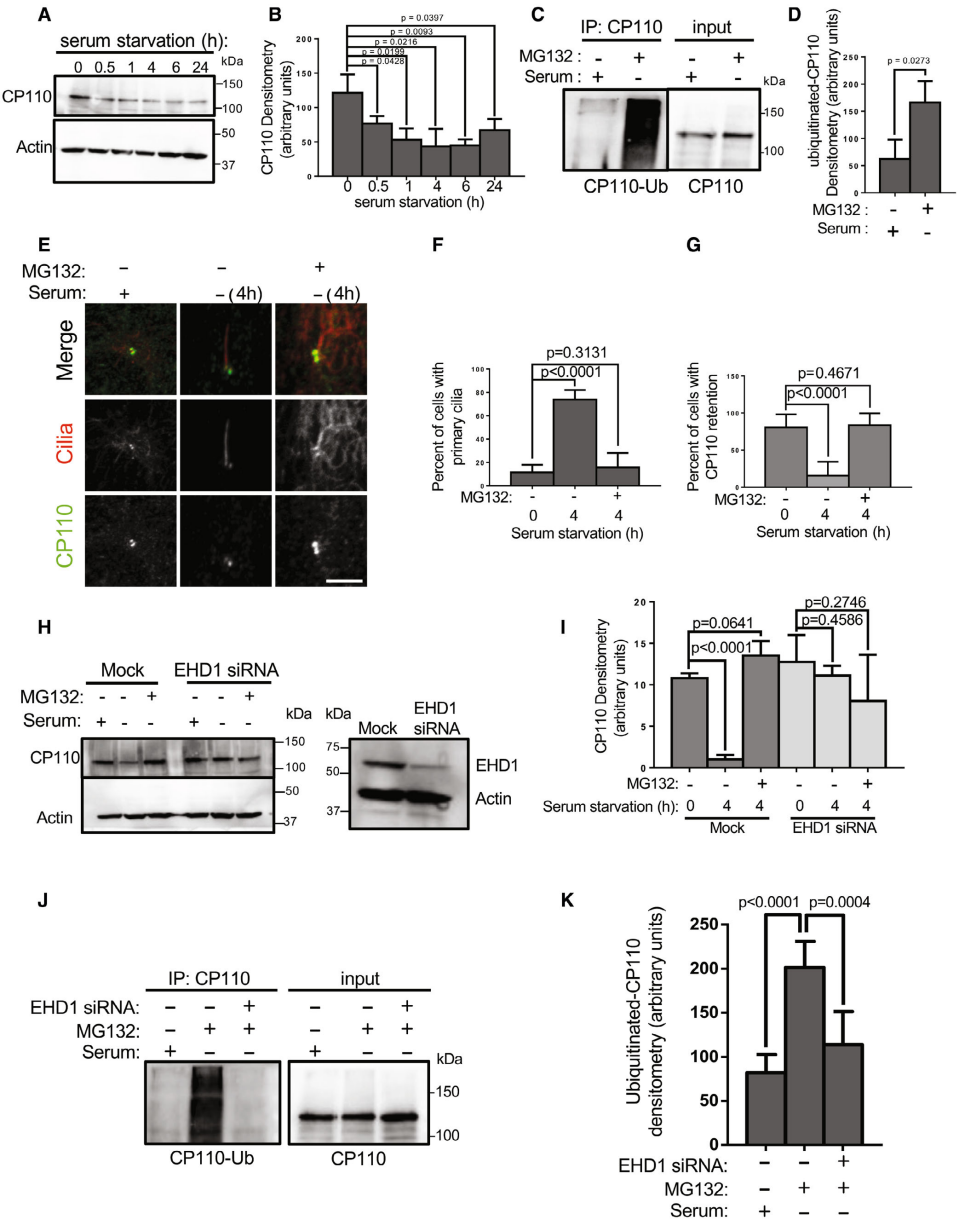
EHD1 depletion impairs CP110 ubiquitination, which is required for CP110 removal from the m‐centriole and ciliogenesis ASerum starvation induces CP110 degradation. RPE‐1 cells were either mock‐treated or serum‐starved for 0.5, 1, 4, 6, and 24 h and then lysed and subjected to immunoblotting with anti‐CP110 antibodies (top panel) and anti‐actin antibodies (loading control; bottom panel).BGraph shows CP110 levels normalized to actin. Normal distribution was determined by the Shapiro–Wilk normality test.CCP110 is ubiquitinated upon serum starvation. RPE‐1 cells were either grown in media with serum, or serum‐starved in the presence of the proteasomal inhibitor, MG132 for 4 h. Cells were then lysed and immunoprecipitated with anti‐CP110, and then immunoblotted with anti‐ubiquitin antibodies (left panel; IP: CP110). The anti‐ubiquitin antibodies were then stripped by incubating the nitrocellulose membrane with 3 M guanidine thiocyanate for 3 min. The membrane was then reblotted with anti‐CP110 (right panel; input).DGraph shows ubiquitinated CP110 normalized to total CP110 immunoprecipitated. Normal distribution was determined by the Shapiro–Wilk normality test.E–GInhibition of proteasomal degradation leads to CP110 stabilization on the m‐centriole and impaired ciliogenesis. RPE‐1 cells on coverslips were either grown in media with serum, serum‐starved for 4 h in the absence of MG132, or serum‐starved for 4 h in the presence of MG132 prior to fixation and immunostaining with antibodies for acetylated tubulin (to detect cilia) and anti‐CP110 antibodies. (E) Representative micrographs showing MG132 treatment impedes removal of CP110 from the m‐centriole and impairs ciliogenesis. Bar, 10 μm. (F) Graph quantifying percent of cells displaying primary cilia. Normal distribution was determined by the D'Agostino and Pearson normality test. (G) Graph quantifying percent of cells with CP110 retention on the m‐centriole. Assumption of normality was not met by the D'Agostino and Pearson normality test, and the Mann–Whitney test was used to determine statistical significance (*P*‐values).H, IEHD1 depletion prevents CP110 degradation upon serum starvation. (H) Mock‐transfected RPE‐1 cells or EHD1 siRNA‐transfected RPE‐1 cells were grown in media containing serum, or serum‐starved for 4 h in the absence or presence of MG132, and then lysed, separated by SDS–PAGE and immunoblotted with anti‐CP110 antibodies (upper panel) or anti‐actin antibodies (loading control; lower panel). Validation of EHD1 depletion is shown in the right panel. (I) Graph displaying densitometric analysis of the intensity of the CP110 bands shown in H normalized to actin (means and *P*‐values are representative of three independent experiments). Normal distribution was determined by the Shapiro–Wilk normality test.J, KEHD1 depletion prevents CP110 ubiquitination upon serum starvation. (J) Mock‐transfected RPE‐1 cells or EHD1 siRNA‐transfected RPE‐1 cells were grown in media containing serum, or serum‐starved for 4 h in the presence of MG132, immunoprecipitated with anti‐CP110 antibodies, and then immunoblotted with anti‐ubiquitin antibodies (IP: CP110; left panel). The blot was then stripped and reimmunoblotted with anti‐CP110 antibodies (right panel). (K) Graph displaying densitometric analysis of ubiquitinated CP110 bands from the left panel of (J) normalized to total CP110 immunoprecipitated. Normal distribution was determined by the Shapiro–Wilk normality test. Data information: All gels portrayed are representative of three individual biological experiments, and graphs displaying standard deviation and *P*‐values are from three individual experiments. More than 50 cells were quantified for each experiment. Statistical significance was calculated with an unpaired two‐tailed *t*‐test for normally distributed samples, or by the Mann–Whitney two‐tailed test for samples that did not meet the assumption of normality. Source data are available online for this figure.

Recent studies suggest that the removal of CP110 from the m‐centriole leads to its degradation. To address this idea, we serum‐starved RPE‐1 cells for 0, 0.5, 1, 4, 6, and 24 h to induce primary ciliogenesis and then assessed total CP110 protein content in each set of cells. As demonstrated, CP110 protein expression progressively decreased between 0.5 and 6 h of serum starvation, remaining significantly lower than the baseline 0 time point even at 24 h (Fig 1A and quantified in B), suggesting that serum starvation promotes degradation of CP110 over time. To determine whether CP110 undergoes ubiquitination upon serum starvation, RPE‐1 cells were serum‐starved for 4 h in the presence of the proteasomal inhibitor MG132. As shown, compared with cells grown in the presence of serum, MG132 treatment stabilized CP110 levels detected in the lysate in serum‐starved cells (Fig 1C input; see similar levels in both lanes). However, upon immunoprecipitation with anti‐CP110 antibodies, a significantly increased level of ubiquitinated CP110 was detected in the serum‐starved/MG132‐treated cells compared with cells maintained in media with serum (Fig 1C, left panel, see immunoprecipitation; quantified in D).

**Figure EV1:**
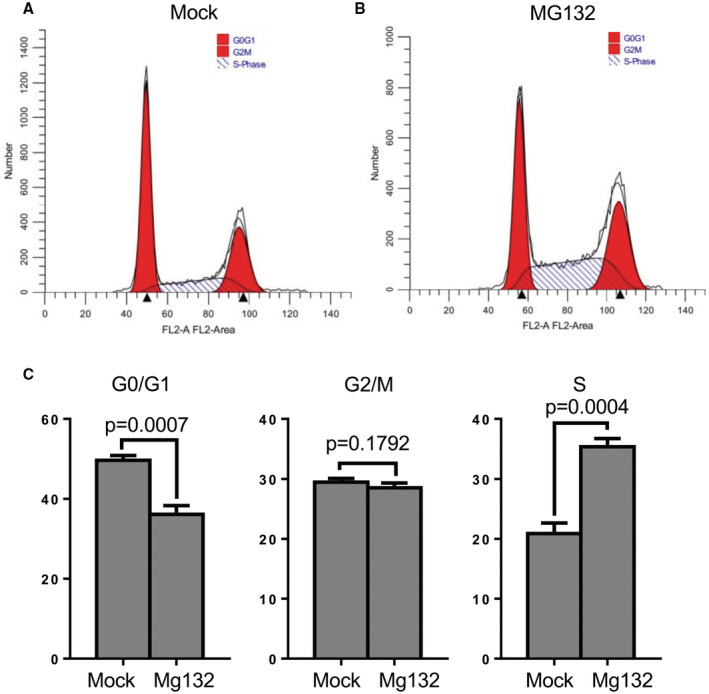
Effect of MG132 on cell cycle A–CRPE‐1 cells were treated with either DMSO (Mock‐treated, A) or MG132 (B) for 4 h, fixed with 70% ethanol at 4°C, stained with Telford reagent at 4°C overnight, and subjected to a flow cytometry cell cycle assay. The percentage of cells in G1/G0, G2/M, and S phase are presented as bar graphs in (C). After normal distribution was determined by the Shapiro–Wilk normality test, statistical significance was calculated with an unpaired two‐tailed *t*‐test. Graphs show standard deviation and *P*‐values from three independent experiments.

We next addressed the impact of inhibiting proteasomal degradation on removal of CP110 from the m‐centriole upon serum starvation. RPE‐1 cells were either grown for 4 h in the presence of serum, in the absence of serum, or in the absence of serum with MG132 treatment (Fig 1E). As observed, in the presence of serum, cilia were rarely detected (Fig 1E, left column, quantified in F) and CP110 was observed on both centrioles over 80% of the time (Fig 1E, left column, quantified in G). Upon 4 h of growth in the absence of serum, primary cilia were generated (Fig 1E, middle column, quantified in F) and CP110 was retained on the m‐centriole about 20% of the time (Fig 1E, middle column, quantified in G). However, when serum‐starved cells were also treated with MG132, few cilia were detected (Fig 1E, right column, quantified in F) and the percentage of cells that retained CP110 on both centrioles was similar to nonstarved cells at ~80% (Fig 1E, right column, quantified in G). Since MG132 treatment has been linked to apoptosis and cell cycle in some cell lines (Han *et al*, 2009; Lee *et al*, 2021), we assessed whether MG132 treatment impacts cell cycle in RPE‐1 cells (Fig EV1). As shown, MG132 treatment does decrease the percentage of cells found in G0/G1 and increase the percentage of cells in S phase (Fig EV1B; quantified in C), compared with mock‐treated cells (Fig EV1A; quantified in C). However, the reduction in G0/G1 cells upon MG132 treatment represents only a ~25% decrease, whereas there is a ~5‐fold (500%) decrease in primary ciliation. Accordingly, for any reduction in primary ciliation observed upon MG132 treatment, only about 5% can likely be attributed to decreased cells in G0/G1 phase. These data show that MG132 treatment leads to impaired CP110 removal from the m‐centriole, raising the possibility that CP110 ubiquitination and degradation are required for primary ciliogenesis in serum‐starved cells.

We have previously shown that the endocytic regulatory protein, EHD1, is required for normal primary ciliogenesis and for the removal of CP110 from the m‐centriole (Lu *et al*, 2015; Jones *et al*, 2022). However, the mechanism by which EHD1 functions in this capacity remains unknown. To evaluate whether EHD1 is required for CP110 degradation, we compared the levels of CP110 upon serum starvation in the presence or absence of MG132, in mock‐transfected or EHD1 siRNA‐transfected RPE‐1 cells (Fig 1H). In the mock‐transfected cells, serum starvation led to a decrease in CP110 levels (Fig 1H; compare lanes 1 and 2 from the left, and quantified in I). However, MG132 treatment again preserved the levels of CP110 in the serum‐starved cells (Fig 1H; lane 3 from the left). In the EHD1 siRNA‐transfected cells, no significant decrease in CP110 levels was observed upon serum starvation, in the absence or presence of MG132 (Fig 1H; lanes 4 and 5 from the left, and quantified in I). Moreover, whereas serum starvation in the presence of MG132 led to increased levels of ubiquitinated CP110, upon EHD1 depletion the CP110 ubiquitination was ablated (Fig 1J, left panel, and quantified in K). Collectively, these data support a role for EHD1 in the regulation of CP110 ubiquitination and degradation leading to primary ciliogenesis.

**Figure 2:**
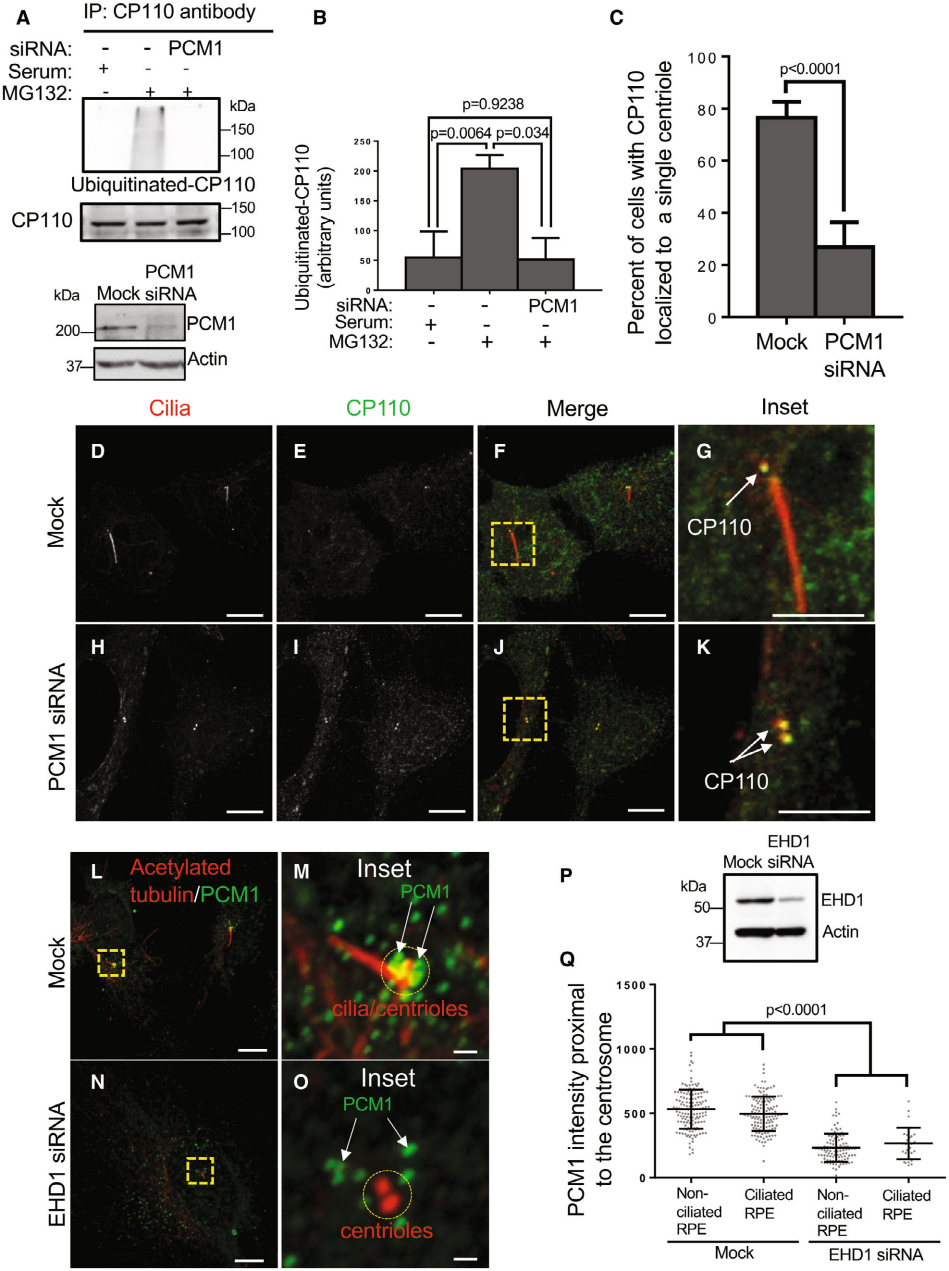
PCM1‐marked centriolar satellites are required for CP110 ubiquitination and its removal from the m‐centriole, and centriolar satellite distribution is regulated by EHD1 A, BPCM1‐depletion impedes CP110 ubiquitination upon serum starvation. (A) Mock‐transfected RPE‐1 cells were maintained in media with serum, and both mock‐transfected and PCM1 siRNA‐transfected cells were serum‐starved for 4 h in the presence of MG132 prior to lysis and immunoblotting with anti‐CP110 antibodies (second panel from top), or first immunoprecipitated with anti‐CP110 antibodies and then immunoblotted with antibodies to detect ubiquitinated CP110 (top panel). Bottom panels depict the efficacy of PCM1 depletion upon PCM1 siRNA‐transfection, with actin as a loading control. (B) Graph displaying densitometric analysis of ubiquitinated CP110 levels (from the upper panel of A) normalized to total CP110 immunoprecipitated. Normal distribution was determined by the Shapiro–Wilk normality test.C–KPCM1 depletion impairs the removal of CP110 from the m‐centriole. (C) Graph displaying the percentage of mock‐transfected and PCM1 siRNA‐transfected RPE‐1 cells with CP110 removed from the m‐centriole upon 4 h of serum starvation. Assumption of normality was not met by the D'Agostino and Pearson normality test, and the Mann–Whitney test was used to determine statistical significance (*P*‐values). (D–G) Serum‐starved mock‐transfected RPE‐1 cells on coverslips were fixed and immunostained with antibodies directed against acetylated tubulin and CP110 and displayed primary cilia generation (marked by acetylated tubulin; D, F and inset in G) and removal of CP110 from the m‐centriole (E–G; see arrow marking CP110 only on the daughter centriole). Bars, (D–F), 10 μm. Bar, (G), 5 μm. (H–K) Serum‐starved PCM siRNA‐transfected RPE‐1 cells on coverslips were fixed and immunostained with antibodies directed against acetylated tubulin and CP110 and displayed limited ciliogenesis (H, J and inset in K) and failure to remove CP110 from the m‐centriole (I–K; see arrows identifying CP110 on both centrioles). Bars (H–J), 10 μm. Bar (K), 5 μm.L–QEHD1 regulates the distribution of centriolar satellites to the centrosomal region. Mock‐transfected RPE‐1 cells on coverslips were serum‐starved and immunostained with antibodies against acetylated tubulin and PCM1 (L; see inset in M), and EHD1 siRNA‐transfected cells on coverslips were serum‐starved and immunostained with antibodies against acetylated tubulin and PCM1 (N; see inset in O). Bars (L and N), 10 μm. Bars (M and O), 1 μm. (P) Anti‐EHD1 antibodies were used in immunoblotting to validate EHD1 depletion in the RPE‐1 cells. (Q) Graphical representation of the mean PCM1 intensity (centriolar satellite intensity) proximal to the centrosome region in serum‐starved RPE‐1 cells that were mock‐transfected or transfected with EHD1 siRNA oligonucleotides. The *P*‐values are calculated for all nonciliated and ciliated mock‐transfected cells compared to all EHD1 siRNA nonciliated and ciliated cells. Note that as expected, among the EHD1‐depleted cells, there are minimal cells that display ciliation. Assumption of normality was not met by the D'Agostino and Pearson normality test, and the Mann–Whitney test was used to determine statistical significance (*P*‐values). Data information: All gels portrayed are representative of three individual experiments, and graphs displaying standard deviation and *P*‐values are from three individual biological experiments. At least 30 cells were quantified for each experiment. Statistical significance was calculated with an unpaired two‐tailed *t*‐test for normally distributed samples, or by the Mann–Whitney two‐tailed test for samples that did not meet the assumption of normality. Source data are available online for this figure.

In addition to the recent identification of endocytic regulatory proteins such as EHD1 (Lu *et al*, 2015; Jones *et al*, 2022), MICAL‐L1 (Xie *et al*, 2019), and the retromer complex (Xie *et al*, 2022), studies have implicated centriolar satellites as crucial regulators of primary ciliogenesis (Kubo *et al*, 1999; Stowe *et al*, 2012; Conkar *et al*, 2017; Odabasi *et al*, 2019; Aydin *et al*, 2020). Accordingly, we hypothesized that centriolar satellite integrity might be required for CP110 ubiquitination. To address this notion, we depleted cells of PCM1 (Fig 2A; bottom panel), a key centriolar satellite component whose ablation disrupts the generation of centriolar satellites (Dammermann & Merdes, 2002; Kubo & Tsukita, 2003). As demonstrated, depletion of PCM1 led to a significant decrease in the level of ubiquitinated CP110 in serum‐starved cells that were treated with MG132 (Fig 2A; quantified in B). We next analyzed the impact of PCM1 depletion on the removal of CP110 from the m‐centriole in serum‐starved cells. Upon serum starvation, mock‐treated RPE‐1 cells typically displayed CP110 localization primarily to the daughter centriole, and primary cilium generation was frequently observed (Fig 2D–G; quantified in C). However, upon PCM1 depletion, CP110 remained associated with the m‐centriole and primary cilia were rarely observed (Fig 2H–K; quantified in C).

**Figure EV2:**
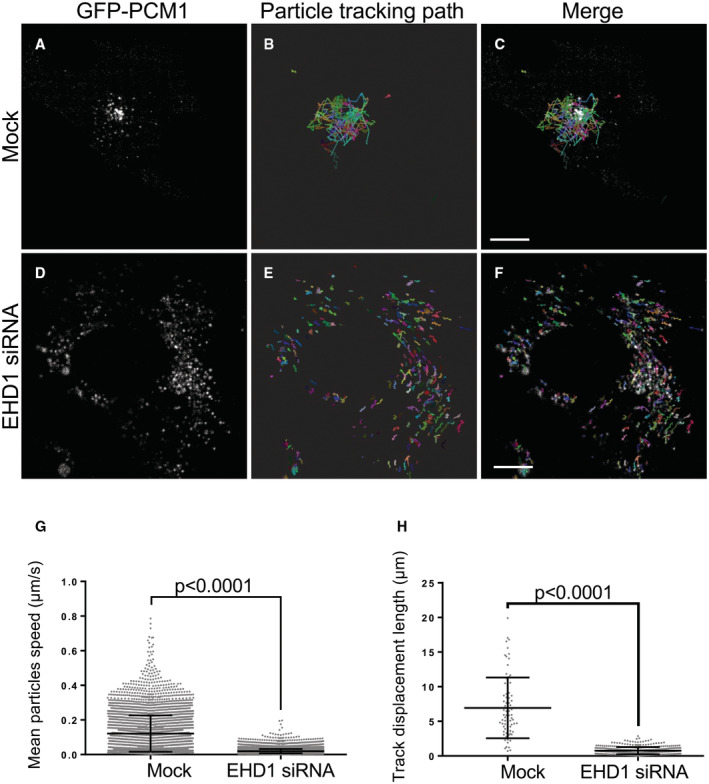
Depletion of EHD1 impedes the distribution of centriolar satellites to the centrosomal region A–FMock‐ (A–C) or EHD‐siRNA‐treated (D–F) RPE‐1 cells were transfected with PCM‐1‐GFP for 24 h and then serum‐starved for 15 min. The distributions and dynamics of PCM‐1 labeled centriolar satellites were monitored by live‐cell imaging. Representative images showing the distributions of PCM‐1‐GFP in the mock‐ (A) or EHD1‐siRNA‐ (D) treated cells are shown. Particle tracking analysis was done with Imaris 9.9.1 software (Oxford Instruments) using an autoregressive motion tracking algorithm with appropriate threshold. Full tracks marking the movement of each detected particle during live‐cell imaging from mock‐treated (B, merged in C) and EHD1‐siRNA‐treated (E, merged in F) RPE‐1 cells are displayed. Each color represents the tracking path of an individual particle detected by the algorithm. Bars, 10 μm.GGraph showing the mean particle speed over 10 min of live‐cell imaging in mock‐ and EHD1‐siRNA‐treated RPE‐1 cells. Assumption of normality was not met by the D'Agostino and Pearson normality test, and the Mann–Whitney test was used to determine statistical significance (*P*‐values).HGraph displaying the mean track displacement length during the 10 min live‐cell imaging in mock‐ and EHD1‐siRNA‐treated RPE‐1 cells. Assumption of normality was not met by the D'Agostino and Pearson normality test, and the Mann–Whitney test was used to determine statistical significance (*P*‐values). Data information: Graphs displaying standard deviation and *P*‐values are from three individual biological experiments. More than 300 particles were detected, and their speeds were calculated and quantified for each experiment. At least 17 particle tracking paths were measured for each experiment. Statistical significance was calculated with the Mann–Whitney two‐tailed test for samples that did not meet the assumption of normality.

It has been demonstrated that centriolar satellites are dynamic and are localized both peripherally to the centrosome and at the centrosome itself, and they may serve as reservoirs that supply the centrosome with regulatory proteins (Aydin *et al*, 2020). Since endocytic regulatory proteins such as EHD1 and centriolar satellites appear to similarly regulate CP110 ubiquitination, removal from the m‐centriole, and primary ciliogenesis, we next hypothesized that EHD1 might regulate centriolar satellite trafficking and access to the m‐centriole, potentially controlling ciliogenesis through the transport and provision of key proteins required for the process. To initially address this idea, we depleted EHD1 from RPE‐1 cells (Fig 2P) and measured the PCM1‐marked centriolar satellites proximal to the cilia or centrioles in mock‐transfected and EHD1‐depleted cells (Fig 2L–O; quantified in Q). As shown, whereas PCM1‐marked centriolar satellites were frequently visible in the immediate radius of the centrosome region, few centriolar satellites were observed in the proximity of the centrosome/centrioles upon EHD1 depletion. Moreover, dynamic movement of PCM1‐containing centriolar satellites could be observed toward (and away) from the centrosomal region in Mock‐transfected cells with a mean particle velocity of ~0.12 μm/s and a mean displacement of ~7 μm over 10 min (Movie EV1 and Fig EV2A–C; quantified in G and H). However, movement was sharply curtailed upon EHD1 depletion, with many centriolar satellites showing a limited range of motion that appears more random and less directed with a mean particle velocity of ~0.02 μm/s and a mean displacement of ~1 μm over 10 min (Movie EV2 and Fig EV2D–F; quantified in G and H). These data support the notion that EHD1 controls centriolar satellite transport to the centrosomal region, which in turn may be required for ubiquitination of CP110.

**Figure 3:**
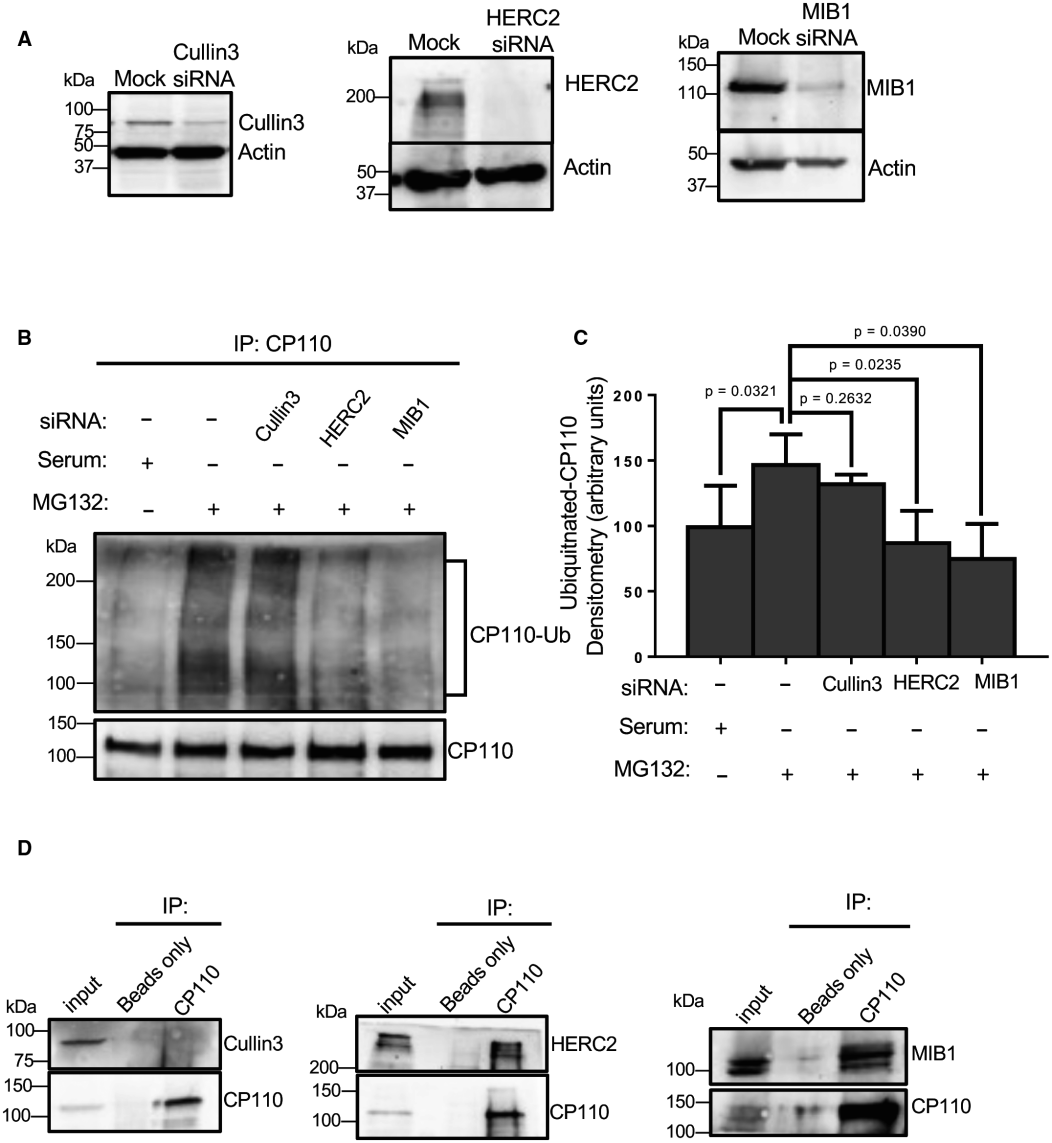
Depletion of HERC2 or MIB1 impedes CP110 ubiquitination upon serum starvation AValidation of siRNA depletion. RPE‐1 cells were mock‐transfected or transfected with siRNA oligonucleotides to impair translation of Cullin3, HERC2, or MIB1. 72 h later the cells were lysed, subjected to SDS–PAGE and immunoblotted. Immunoblotting with anti‐actin antibodies was done as a loading control.BHERC2 or MIB1 depletion impairs CP110 ubiquitination. RPE‐1 cells were grown in serum (first lane from left), or with MG132 in the absence of serum for 4 h (second lane from left), transfected with Cullin3 siRNA oligonucleotides and treated with MG132 in the absence of serum for 4 h (third lane from left), transfected with HERC2 siRNA oligonucleotides and treated with MG132 in the absence of serum for 4 h (fourth lane from left), or transfected with MIB1 siRNA oligonucleotides and treated with MG132 in the absence of serum for 4 h (fifth lane from left). Cells were lysed and immunoprecipitated with anti‐CP110 antibodies, and the resultant immunoprecipitates were immunoblotted with anti‐ubiquitin to identify ubiquitinated CP110 (upper panel). The blot was then stripped and reimmunoblotted with anti‐CP110 to reveal the amount of CP110 pulled down from the lysate (lower panel).CGraph displaying densitometric analysis of ubiquitinated CP110 bands normalized to total CP110 immunoprecipitated. Normal distribution was determined by the Shapiro–Wilk normality test, and statistical significance was calculated with an unpaired two‐tailed *t*‐test.DCP110 interacts with HERC2 and MIB1, but not Cullin3. RPE‐1 cells were lysed and immunoprecipitated with beads only or anti‐CP110, and then subjected to SDS–PAGE and immunoblotting with CP110 to validate the immunoprecipitation (lower panel of each gel) and either Cullin3 (left gel, upper panel), HERC2 (middle gel, upper panel) or MIB1 (right gel, upper panel). 7% of inputs are shown on the left of each gel. Data information: All gels are representative ones from three individual biological experiments. Graph displays mean densitometry representing ubiquitination of CP110 from three individual experiments, bars indicate standard deviation, and statistical significance was calculated with an unpaired two‐tailed *t*‐test, since the Shapiro–Wilk test indicated a normal distribution. Source data are available online for this figure.

Given the idea that centriolar satellites may be required for CP110 ubiquitination, we hypothesized that these satellites may host a select E3 ligase that is transported to the m‐centriole to ubiquitinate CP110 and promote ciliogenesis. We therefore sought to test the potential role of two E3 ligases that had been previously identified as components of centriolar satellites: HECT domain and RCC1‐like domain 2 (HERC2; Quarantotti *et al*, 2019) and mindbomb homolog 1 (MIB1; Villumsen *et al*, 2013; Cajanek *et al*, 2015; Wang *et al*, 2016; Tozer *et al*, 2017), along with Cullin3, an E3 ligase previously implicated in ciliogenesis (Kasahara *et al*, 2014; Nagai *et al*, 2018) but not identified as a centriolar satellite component. Accordingly, we individually depleted Cullin3, HERC2, and MIB1 from RPE‐1 cells (Fig 3A) and then assessed the level of CP110 ubiquitination in serum‐starved cells treated with MG132 (Fig 3B, quantified in C). As demonstrated, serum‐starved cells displayed a significant level of CP110 ubiquitination compared with cells with serum (Fig 3B; compare lanes 1 and 2, quantified in C). However, depletion of HERC2 or MIB1 (but not Cullin3) led to dramatically reduced CP110 ubiquitination, similar to nonstarved cells, suggesting that HERC2 and/or MIB1 may be required for CP110 ubiquitination.

We next hypothesized that if HERC2 and MIB1 are capable of ubiquitinating CP110, they might assemble in a protein complex. To test this, we performed co‐immunoprecipitation experiments to determine whether CP110 interacts with each of these E3 ligases, as well as Cullin3. As demonstrated, whereas CP110 antibodies precipitated CP110, no Cullin3 was observed precipitating with CP110 (Fig 3D, left panel). However, CP110 antibodies pulled down both HERC2 and MIB1 (Fig 3D, middle and right panels), suggesting that these ligases interact with and ubiquitinate CP110.

**Figure 4:**
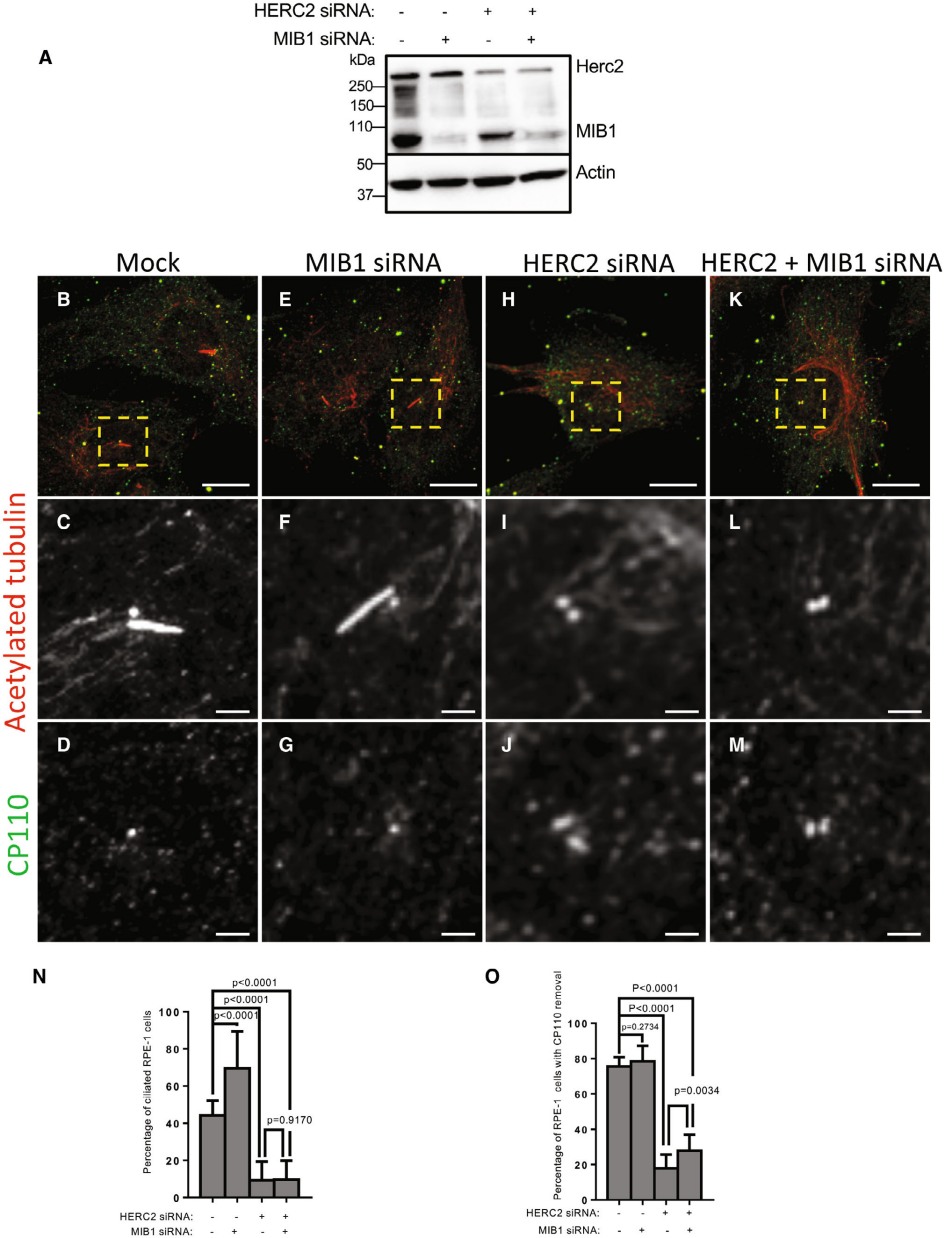
Depletion HERC2 but not MIB1 impairs primary ciliogenesis and CP110 removal from the m‐centriole AValidation of siRNA depletion. RPE‐1 cells were mock‐transfected or transfected with MIB1 siRNA oligonucleotides, HERC2 siRNA oligonucleotides, or transfected simultaneously with oligonucleotides directed against both MIB1 and HERC2. Lysates were collected, subjected to SDS–PAGE and immunoblotted with the designated antibodies as shown. Anti‐actin antibodies were used as a loading control (lower panel).B–M(B–D) Mock‐transfected cells, (E–G) MIB1 siRNA‐transfected cells, (H–J) HERC2 siRNA‐transfected cells, or (K–M) MIB1 and HERC2 siRNA‐transfected cells on coverslips were fixed and immunostained with antibodies against acetylated tubulin (insets; C, F, I, L: merged field; B, E, H, K) and CP110 (insets; D, G, J, M: merged field; B, E, H, K). Bars (B, E, H, K), 10 μm. Bars (C, D, F, G, I, J, L, M), 2 μm.NGraph displaying the percentage of ciliated RPE‐1 cells upon serum starvation for mock‐transfected, MIB1 siRNA‐transfected, HERC2 siRNA‐transfected and simultaneous MIB1 siRNA‐transfected and HERC2 siRNA‐transfected cells. Normal distribution was determined by the D'Agostino and Pearson normality test.OGraph displaying the percentage of RPE‐1 cells with CP110 on a single centriole upon serum starvation for mock‐transfected, MIB1 siRNA‐transfected, HERC2 siRNA‐transfected and simultaneous MIB1 siRNA‐transfected and HERC2 siRNA‐transfected cells. Normal distribution was determined by the D'Agostino and Pearson normality test. Data information: All gels are representative ones from three individual experiments. Graphs show standard deviation and *P*‐values from three independent biological experiments. More than 100 cells were quantified for each experiment. Statistical significance was calculated with an unpaired two‐tailed *t*‐test. Source data are available online for this figure.

Given the potential roles of the centriolar satellite E3 ligases MIB1 and HERC2 in CP110 ubiquitination, we reasoned that either or both proteins might be required for the removal of CP110 from the m‐centriole and generation of the primary cilium. To test this, RPE‐1 cells were either mock‐transfected, or depleted of MIB1, HERC2 or both MIB1 and HERC2 together (Fig 4A). We then serum‐starved cells on coverslips and assessed primary cilia generation and CP110 removal from the m‐centriole (Fig 4B–M; quantified in N and O). As demonstrated, both mock‐transfected cells (Fig 4B–D) and MIB1‐depleted cells (Fig 4E–G) displayed ciliogenesis in 40–65% of serum‐starved cells (quantified in N). Similarly, CP110 was removed from the m‐centriole in ~80% of mock‐transfected and MIB1‐depleted cells (Fig 4B–G; quantified in O). Alternatively, HERC2 depletion prevented primary cilia generation (Fig 4H–J; quantified in N) and limited the removal of CP110 from the m‐centriole (Fig 4H–J; quantified in O). Interestingly, depletion of both HERC2 and MIB1 together led to impaired ciliogenesis and removal of CP110 from the m‐centriole similar to depletion of HERC2 alone (Fig 4K–M; quantified in N and O). Taken together, these data suggest that HERC2 is a crucial centriolar satellite E3 ligase that is required for CP110 ubiquitination, its removal from the m‐centriole, and generation of the primary cilium.

**Figure 5:**
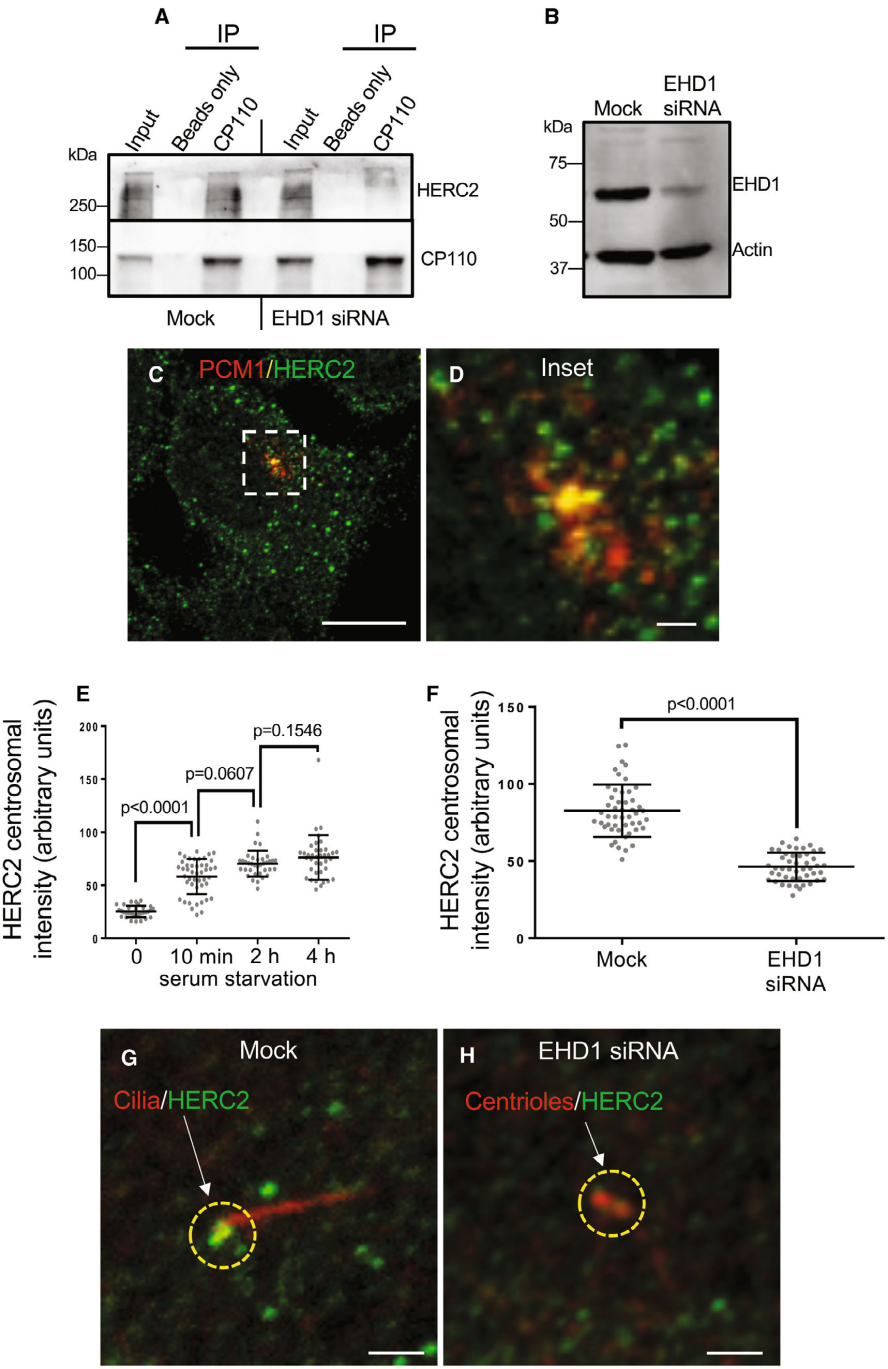
EHD1 is required for HERC2 localization to the m‐centriole and binding to CP110 AEHD1 is required for the interaction between HERC2 and CP110. RPE‐1 cells were mock‐transfected or transfected with EHD1 siRNA oligonucleotides for 72 h. Cells were lysed and immunoprecipitated with anti‐CP110 or beads only (control) and separated by SDS–PAGE along with 7% of the lysate (input). After transfer to nitrocellulose, antibodies were used to detect CP110 (lower panel) and HERC2 (upper panel).BImmunoblotting of mock‐transfected and EHD1 siRNA‐transfected cells demonstrating the efficacy of EHD1 depletion. Actin was used as a loading control.C, DHERC2 partially localizes to PCM1‐containing centriolar satellites. RPE‐1 cells were serum‐starved for 4 h, fixed and immunostained with antibodies against PCM1 (red) and HERC2 (green). The micrographs show a single representative cell (C) and an inset (D). Bar (C), 10 μm. Bar (D), 1 μm.ERPE‐1 cells were serum‐starved for 0, 10 min, 2 h, or 4 h, fixed and immunostained with anti‐HERC2 antibodies to quantify centrosomal HERC2. Quantification was done by drawing a circular region of interest of 2.5 μm in diameter around the centrosome/basal body of each cell and measuring the intensity of HERC2 localized to this area. Assumption of normality was not met by the D'Agostino and Pearson normality test, and the Mann–Whitney test was used to determine statistical significance (*P*‐values).FMock‐transfected or EHD1 siRNA‐transfected RPE‐1 cells were serum‐starved for 4 h, fixed and immunostained with anti‐HERC2 antibodies to quantify centrosomal HERC2 as described in (E). Assumption of normality was not met by the D'Agostino and Pearson normality test, and the Mann–Whitney test was used to determine statistical significance (*P*‐values).G, HRepresentative micrographs of RPE‐1 cells on coverslips that were mock‐transfected (G) or EHD1 siRNA‐transfected (H) and immunostained with antibodies to acetylated tubulin (red) or HERC2 (green). Dashed yellow regions of interest highlight the centrosomal area. Arrows denote the HERC2 signal observed in each micrograph. Bars, 1 μm. Data information: All gels are representative ones from three individual experiments. Graphs show standard deviation and *P*‐values from three independent biological experiments. More than 50 cells were quantified in each experiment. Statistical significance was calculated with the Mann–Whitney two‐tailed test for samples that did not meet the assumption of normality. Source data are available online for this figure.

Given the potentially significant role for HERC2 in the regulation of primary ciliogenesis, we next hypothesized that EHD1 may control HERC2 transport on centriolar satellites and thus regulate its distribution and interaction with CP110. Therefore, we reasoned that EHD1 depletion might lead to reduced interactions between HERC2 and CP110. To address this, we depleted RPE‐1 cells of EHD1 and compared the ability of CP110 antibodies to co‐precipitate HERC2 from mock‐transfected and EHD1‐depleted cells (Fig 5A and B). As demonstrated, HERC2 could be observed precipitating with CP110 in mock‐transfected cells, but not EHD1‐depleted cells. To support the notion that EHD1 regulates HERC2 and centriolar satellite movement to the centriolar region, we first demonstrated that HERC2 displayed partial co‐localization with the centriolar satellite marker, PCM1 (Fig 5C and D). We then measured the level of HERC2 in the radius of the centrosome in the presence of serum, and after serum starvation for 10 min, 2 h, and 4 h, and observed a progressive increase in HERC2 localization to the centriolar region over time (Fig 5E). Finally, to address whether EHD1 was required for the redistribution of HERC2 from the periphery to the centriolar region upon serum starvation, we depleted EHD1 and measured HERC2 recruitment to the centriolar area. As demonstrated, EHD1 depletion led to a significant decrease in the level of HERC2 at the centriolar region (Fig 5G and H; quantified in F). Collectively, these data support the notion that EHD1 serves to regulate the access of centriolar satellite proteins, such as HERC2, to the centriolar region where it ubiquitinates CP110 in response to serum starvation.

**Figure 6:**
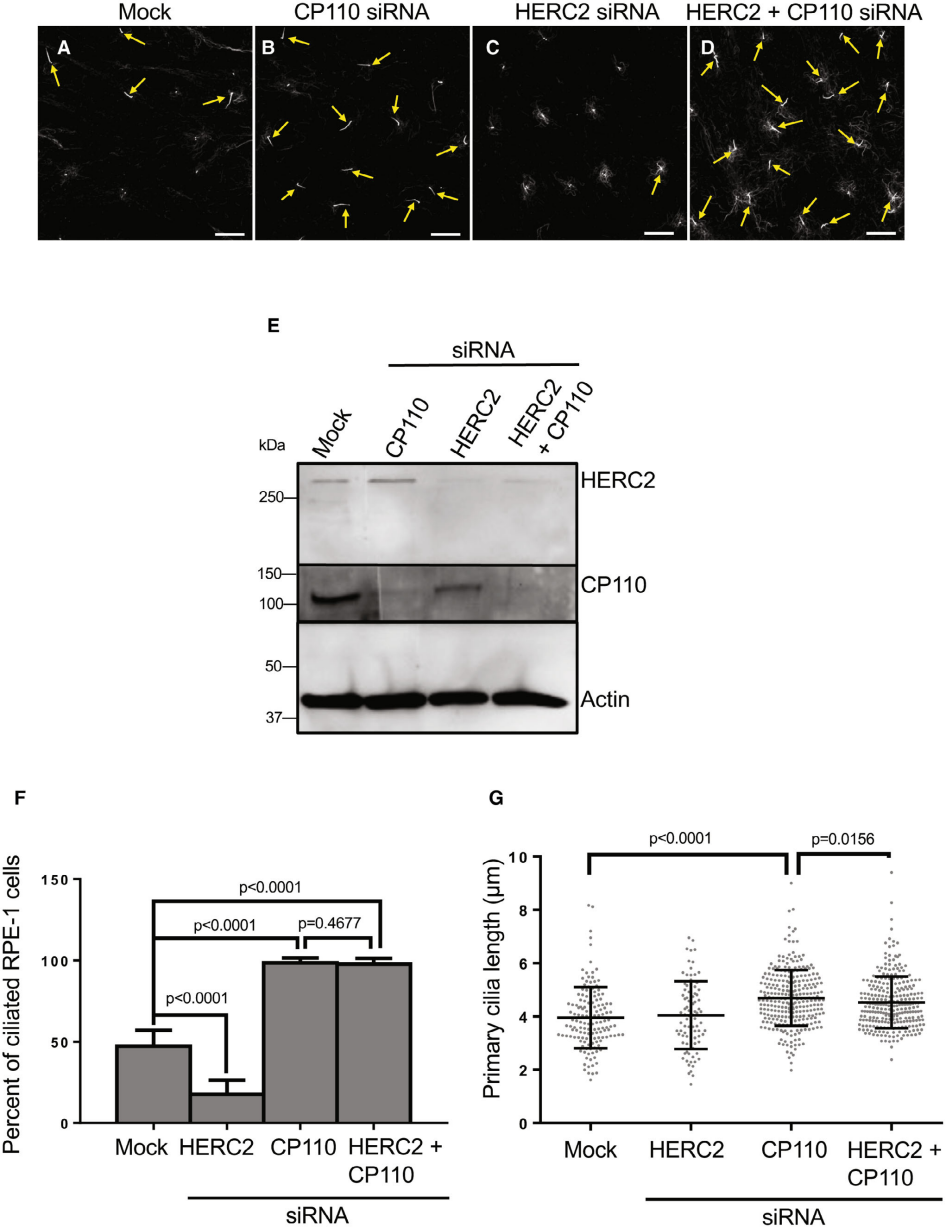
CP110 depletion overrides the ciliogenesis defect observed upon HERC2 depletion A–DRPE‐1 cells on coverslips were mock‐transfected (A), transfected with CP110 siRNA oligonucleotides (B) transfected with HERC2 siRNA oligonucleotides (C), or transfected with both CP110 and HERC2 siRNA oligonucleotides (D). Cells were fixed and immunostained with acetylated tubulin to detect primary cilia (yellow arrows). Bars, 10 μm.EImmunoblot demonstrating the efficacy of depletion for CP110 (middle panel), HERC2 (top panel), and simultaneous CP110 and HERC2 depletion (middle and top panels). Actin was used as a loading control (bottom panel).FGraph displaying the percentage of RPE‐1 cells with primary cilia (quantified from the experiments depicted by the representative micrographs in A–D). Assumption of normality was not met by the D'Agostino and Pearson normality test, and the Mann–Whitney test was used to determine statistical significance (*P*‐values).GGraph depicting the mean length of primary cilia from serum‐starved RPE‐1 cells that were mock‐transfected, transfected with HERC2 siRNA oligonucleotides, transfected with CP110 siRNA oligonucleotides, or transfected with both CP110 and HERC2 siRNA oligonucleotides. Assumption of normality was not met by the D'Agostino and Pearson normality test, and the Mann–Whitney test was used to determine statistical significance (*P*‐values). Data information: Graphs show standard deviation and *P*‐values from three independent experiments. The micrographs (A–D) and immunoblots (E) are representative ones from the three individual biological experiments. More than 50 cells were quantified in each experiment, although fewer ciliated cells were observed and measured under the HERC2 knockdown condition. Statistical significance was calculated with the Mann–Whitney two‐tailed test for samples that did not meet the assumption of normality. Source data are available online for this figure.

Thus far, our findings suggest that HERC2 is a key E3 ligase that localizes to centriolar satellites and is actively transported to the centriolar region to affect the ubiquitination of CP110, its removal and degradation, and the generation of the primary cilium. To further test this model, RPE‐1 cells on coverslips were mock‐transfected, CP110 depleted, or HERC2 depleted (Fig 6A–C; knockdowns validated in E). Upon serum starvation, about 50% of cells displayed a primary cilium (Fig 6A; quantified in F). When CP110 was depleted, this allowed unrestrained ciliary generation and nearly 100% of cells generated a primary cilium when serum‐starved (Fig 6B; quantified in F). On the contrary, knockdown of HERC2 expression dramatically reduced cilia generation to about threefold lower than the levels observed in mock‐transfected cells (Fig 6C; quantified F). To further validate that HERC2 functions via ubiquitination and control of CP110 removal from the m‐centriole, we depleted both HERC2 and CP110 from RPE‐1 cells (validated in Fig 6E). When simultaneous knockdown of both HERC2 and CP110 was done, the impaired ciliogenesis observed with HERC2 depletion alone was rescued and the level of ciliogenesis approached 100% of cells, similar to CP110 depletion alone (Fig 6D; quantified in F). Knockdown of CP110 alone or CP110 together with HERC2 also led to a small but significant increase in cilia length (Fig 6G). Overall, these data imply that HERC2 functions upstream of CP110, and its involvement in ciliogenesis stems from its ability to ubiquitinate CP110 and induce its proteasomal degradation.

## Discussion

The removal of CP110 along with its interaction partner, CEP97, from the m‐centriole represents a crucial stage of regulation in the generation of the primary cilium (Spektor *et al*, 2007; Tsang *et al*, 2008; Kobayashi *et al*, 2011). It is therefore not surprising that a variety of different mechanisms have been proposed for CP110 removal from the m‐centriole, including putative vesicular transport pathways, and proteolytic mechanisms for its degradation. Despite a growing number of proteins recently identified having involvement in the regulation of this process (Kobayashi *et al*, 2014; Prosser & Morrison, 2015; Huang *et al*, 2018; Lo *et al*, 2019; Goncalves *et al*, 2021; Liu *et al*, 2021; Shen *et al*, 2022; Song *et al*, 2022), information remains sparse on the precise mechanisms by which CP110 removal is accomplished, and how these proteins/mechanisms might be coordinated.

Several endocytic regulatory proteins have also been linked to CP110 removal from the m‐centriole. Most notably, Rab11 and EHD1 both regulate primary ciliogenesis and CP110 removal from the m‐centriole (Knodler *et al*, 2010; Lu *et al*, 2015), as do MICAL‐L1 (Xie *et al*, 2019) and the retromer complex (Xie *et al*, 2022). However, in addition to endocytic proteins, centriolar satellites have also been directly implicated in ciliogenesis (Kubo *et al*, 1999; Stowe *et al*, 2012; Lee & Stearns, 2013; Villumsen *et al*, 2013; Odabasi *et al*, 2019; Prosser & Pelletier, 2020). Despite these exciting findings, it is unclear what role the centriolar satellites play in facilitating ciliogenesis. Moreover, whether endocytic regulatory proteins coordinate control of ciliogenesis with centriolar satellites remains unknown.

**Figure 7:**
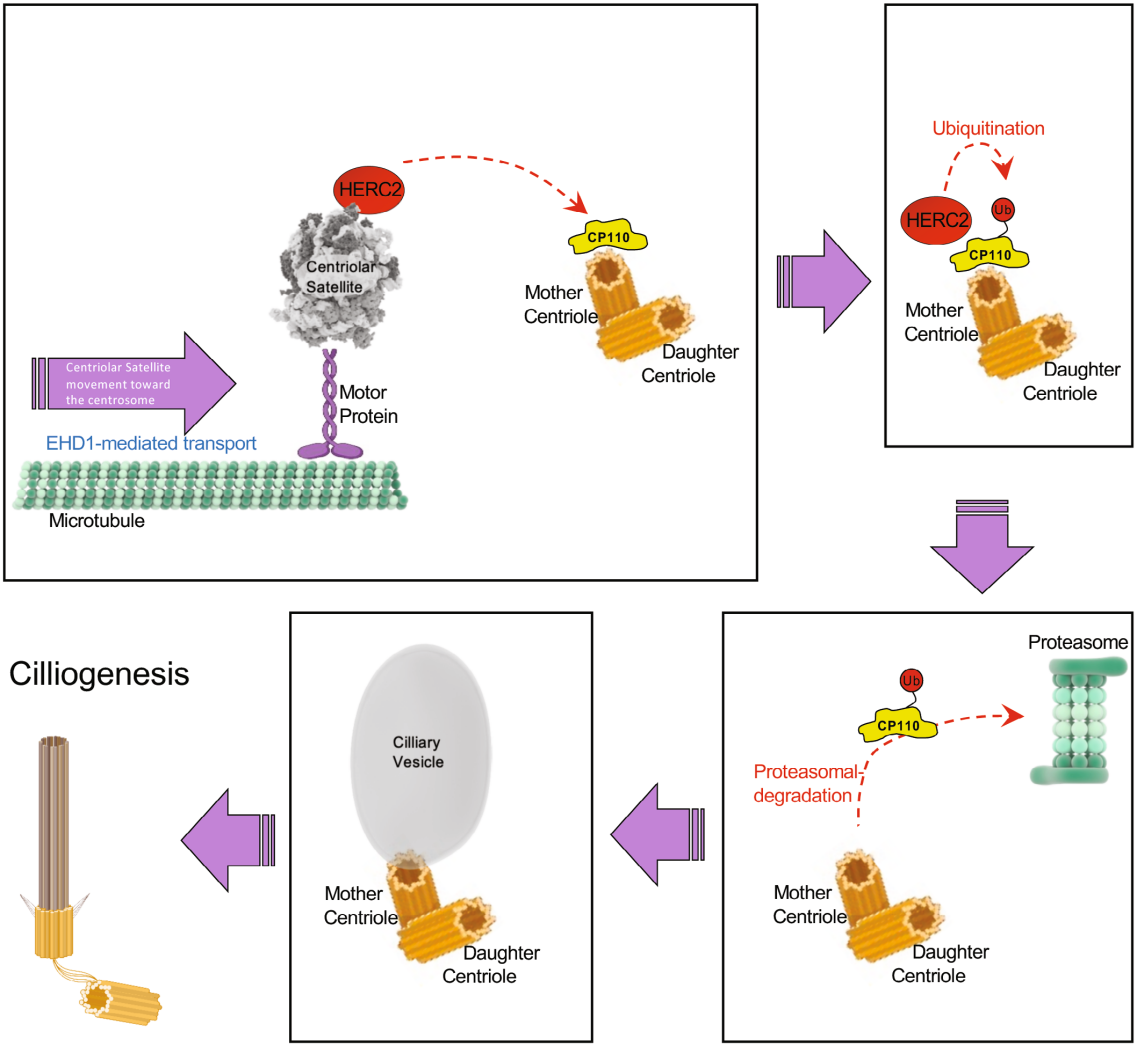
Model for the removal of CP110 from the m‐centriole upon initiation of ciliogenesis Upon serum starvation or initiation of ciliogenesis, EHD1 mediates microtubule‐dependent trafficking of centriolar satellites carrying HERC2 to the centriolar region. This in turn facilitates an interaction between HERC2 and CP110 on the m‐centriole, leading to its ubiquitination and degradation in the proteasome. As a result, the ciliary vesicle forms and the axoneme is extended to generate the primary cilium. Figure prepared using BioRender software license # AF256M9O6P.

**Figure EV3:**
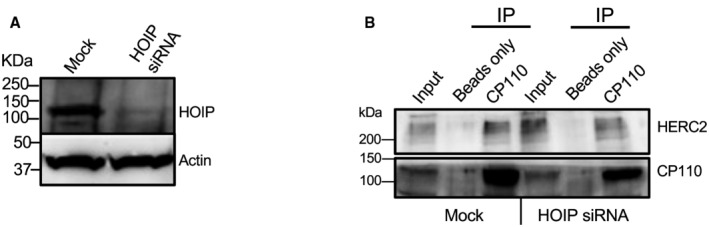
Interaction between CP110 and HERC2 is independent of LUBAC component HOIP Validation of HOIP depletion in RPE‐1 cells by immunoblotting.RPE‐1 cells following mock treatment or HOIP siRNA treatment were lysed and immunoprecipitated with beads‐only control, or anti‐CP110 antibodies, and subjected to SDS–PAGE and immunoblotting with antibodies directed against CP110 (lower panel) and HERC2 (upper panel). 7% of inputs are shown on the left lane of the gel. The presented gel is a representative of three individual experiments.

Here, we describe a novel mechanism to explain a coordinated role for the endocytic regulatory protein, EHD1, with centriolar satellites in the delivery of HERC2 to the m‐centriole. We have demonstrated that this E3 ligase interacts with CP110 to affect its ubiquitination, thus targeting the latter for proteasomal degradation and facilitating ciliary vesicle growth and ciliogenesis (see Fig 7). Ubiquitination of CP110 has been described for the process of centrosome duplication and homeostasis by the SCF(Cyclin F) ubiquitin ligase complex (D'Angiolella *et al*, 2010; Li *et al*, 2013) and by the EDD‐DYRK2‐DDB1^VprBP^ complex (Hossain *et al*, 2017). However, only very recently has ubiquitination been identified as a requirement for CP110 removal from the m‐centriole by the linear ubiquitin chain assembly complex (LUBAC; Shen *et al*, 2022). We have identified the E3 ligase HERC2 as crucial for the ubiquitination of CP110, its removal from the m‐centriole, and primary ciliogenesis. HERC2 was previously identified as an interaction partner for CP110 on the centrosome (Al‐Hakim *et al*, 2012), it has been identified as a potential component of centriolar satellites (Odabasi *et al*, 2019), and a recent study supports a role for it in ciliogenesis (Quarantotti *et al*, 2019). Our data, while supporting a mechanism in which HERC2 is delivered to the m‐centriole by centriolar satellites, do not rule out potential coordination between HERC2 and LUBAC; indeed, it is possible that linear chain ubiquitination may first require a regulated step of ubiquitination by HERC2 on an ε‐amino group of a lysine residue on CP110. In such a scenario, both ligases would be required for effective CP110 removal from the m‐centriole, and depletion of either ligase would prevent effective primary ciliogenesis. Indeed, an initial series of experiments demonstrates that knockdown of the LUBAC component HOIP (Fig EV3A) does not interfere with the interaction between HERC2 and CP110 (Fig EV3B), suggesting that LUBAC function may be downstream of HERC2 activity. A study just published also indicates a role for ubiquitination of the octameric BBSome, a complex that regulates receptor trafficking within the primary cilium (Chiuso *et al*, 2023). Accordingly, a complete understanding of the orchestration of HERC2, LUBAC, and potentially additional E3 ligases in CP110 ubiquitination and removal remains an important goal of future studies.

While our data support a multistep process in which centriolar satellite movement to the centrosome region is controlled by EHD1, thus regulating HERC2 delivery to CP110 at the m‐centriole, we recognize that the mechanism by which EHD1 controls centriolar satellite movement along microtubules remains to be elucidated. Previous hints at EHD1's potential role in the regulation of the microtubule network arise from several directions. First, we have previously shown that the distribution of endosomes within the cell is dramatically altered upon EHD1 depletion (Caplan *et al*, 2002). This distribution pattern could result from indirect effects on the microtubules via the EHD1 interaction partner MICAL‐L1 and its binding protein, CRMP2, the latter which interacts with tubulin dimers and kinesin to negatively regulate dynein‐based transport (Rahajeng *et al*, 2010). Moreover, MICAL‐L1 interacts directly with γ‐tubulin (Xie *et al*, 2019). EHD1 also regulates cytokinesis and central spindle formation hinting at a role in microtubule regulation (Reinecke *et al*, 2015). Most recently, EHD1 has been identified in an interaction with the TUBB3 gene product and postulated to affect microtubule instability (Huang *et al*, 2020). Whether EHD1 directly affects microtubule dynamics remains an important future goal.

Our study connects between endocytic regulation, centriolar satellite movement/distribution and the delivery of a key centriolar satellite‐localized E3 ubiquitin ligase, HERC2, to the m‐centriole region. HERC2 subsequently ubiquitinates CP110 and spurs its removal from the m‐centriole to facilitate ciliogenesis. Particularly, compelling is the requirement for EHD1 in centriolar satellite mobility and HERC2 localization to the centrosomal region and the failure of CP110 to undergo ubiquitination in the absence of EHD1. In addition, the ability of CP110 knockdown to override HERC2 depletion and allow ciliogenesis to proceed strongly supports the notion that the role of HERC2 in ciliogenesis is to ubiquitinate and remove CP110 from the m‐centriole. Overall, our study further elucidates the role of centriolar satellites and EHD1 in ciliogenesis and provides new insight into the mechanism of CP110 removal upon its ubiquitination by the E3 ligase HERC2.

## Materials and Methods

### Antibodies and reagents

Primary and secondary antibodies used in this study were rabbit anti‐human Ubiquitin (P37, cat. no. 58395; Cell Signaling Technology, Danvers, MA), rabbit anti‐human PCM1 (G2000, cat. no. 5213; Cell Signaling Technology), rabbit anti‐human acetylated tubulin (K40, cat. no. 3971; Cell Signaling Technology), rabbit anti‐human Cullin3 (cat. no. 2759; Cell Signaling Technology), mouse anti‐human acetylated tubulin (cat. no. T7451; Sigma‐Aldrich, St. Louis, MO), rabbit anti‐human MIB1 (cat. no. HPA019100; Sigma‐Aldrich), rabbit anti‐human CP110 (cat. no. 12780‐1‐AP; Proteintech, Rosemont, IL), mouse anti‐human HERC2 (cat. no. 612366; BD Biosciences, Haryana, India), mouse anti‐human PCM1 (cat. no. sc‐398365; Santa Cruz Biotechnology, Dallas, TX), and rabbit anti‐human EHD1 (cat. no. ab109311; Abcam, Somerset, NJ), mouse anti‐human pan‐actin (cat. no. NB600‐535; Novus, Littleton, CO), rabbit anti‐HOIP (cat. no. PA5‐114380; Invitrogen, Waltham, MA), goat anti‐mouse horseradish peroxidase (HRP), (cat. no. 115‐035‐003; Jackson ImmunoResearch Laboratories, West Grove, PA), donkey anti‐rabbit HRP (cat. no. NA934V; GE Healthcare, Pittsburgh, PA), Alexa 568–conjugated goat anti‐mouse (cat. no. A11031; Life Technologies, Carlsbad, CA), and Alexa 488–conjugated goat anti‐rabbit (cat. no. A11034; Life Technologies). The proteasome inhibitor MG132 was obtained from Millipore (St. Louis, MO).

### Cell culture and treatments

Fresh original stock from the human epithelial cell line hTERT RPE‐1 (ATCC‐CRL4000) was grown at 37°C in 5% CO_2_ in DMEM/F12 (Thermo Fisher Scientific, Carlsbad, CA) containing 10% fetal bovine serum (FBS; Sigma‐Aldrich), 2 mM L‐glutamine, 100 U/ml penicillin/streptomycin and 1× nonessential amino acids (ThermoFisher Scientific, Waltham, MA). All cell lines in the laboratory are also grown in 100 μg/ml Normocin (Fisher Scientific, Waltham, MA) to prevent mycoplasma and other contamination and routinely tested for mycoplasma contamination. To induce ciliogenesis, RPE‐1 cells were incubated in DMEM/F12 with 2 mM L‐glutamine, 1× nonessential amino acids and 0.2% FBS for the indicated times. The pEGFP‐N‐PCM1 construct was a kind gift from Andreas Merdes (Université Toulouse III, Toulouse, FR). For live‐cell imaging, RPE‐1 cells were transfected with 1.5 μg of DNA construct using Lipofectamine 2000 (Invitrogen, Carlsbad, CA) for 24 h in antibiotics‐free culture medium according to the manufacturer's protocol. Small interfering siRNA (siRNA) oligonucleotides targeting human EHD1 (5′‐CCAAUGUCUUUGGUAAAGA[dT][dT]‐3′), human HERC2 (5′‐GAAUCAGCAGCUACGAUAA[dT][dT]‐3′), human MIB1 (5′‐CUGUUAGAGUCUCGUAGGA[dT][dT]‐3′), human PCM1 (5′‐CAGACUUCCCUCCAGGCUA[dT][dT]‐3′), and human HOIP (5′‐GAGAUGUGCUGCGAUUAUA[dT][dT]‐3′) were purchased from Sigma‐Aldrich. ON‐TARGETplus SMARTpool siRNA targeting human CP110 (5′‐GGACAUACCUAUACGAACU‐3′, 5′‐GCAAUUAUCACUACUCAUA‐3′, 5′‐UGAGAGAGGCGCACACAUA‐3′, 5′‐GUACGGUAUUCAUGACAUA‐3′) were synthesized by Dharmacon (Lafayette, CO), 200 nM of oligonucleotides were transfected in RPE‐1 cells with Lipofectamine RNAi/MAX (Invitrogen, Carlsbad, CA) for 72 h in the absence of antibiotics, as per the manufacturer's protocol. The efficiency of knockdown was determined by immunoblotting.

### Cell cycle analysis

Mock‐ and MG132‐treated RPE‐1 cells were collected, washed with PBS, and then fixed with 70% ethanol at 4°C for 1 h. The fixed cells were washed with PBS, and stained with Telford Reagent (0.115 mM EDTA, 13.4 mg RNAse A (93 U/mg), 0.0748 mM Propidium Iodide, and 1% TX‐100 in 500 ml PBS) at 4°C overnight and subjected to flow cytometry analysis at the UNMC Flow Cytometry Research Facility.

### Co‐immunoprecipitation

Mock‐ or EHD1‐siRNA‐treated RPE‐1 cells growing in 100 mm dishes were lysed in lysis buffer containing 50 mM Tris–HCl pH 7.4, 150 mM NaCl, 1 mM MgCl_2_, 1% Triton X, and freshly added protease inhibitor cocktail (Roche, Basel, Switzerland). Cell debris was eliminated by centrifugation at 1889 *g* at 4°C for 10 min. The cleared lysate was collected, and 7% of the sample lysates were taken directly as input controls. The rest of the lysates were subjected to overnight incubation with anti‐CP110 antibodies at 4°C. Protein G‐agarose beads (GE Healthcare) were added to the lysate–antibody mix and left to rock at 4°C for 2 h. Samples were then washed three times with washing buffer containing 50 mM Tris–HCl pH 7.4, 150 mM NaCl, 1 mM MgCl_2_, and 0.1% Triton X‐100. Protein complexes were eluted from the beads by boiling the sample for 10 min in the presence of 4× loading buffer containing 250 mM Tris–HCl pH 6.8, 8% SDS, 40% glycerol, 5% β‐mercaptoethanol, and 0.2% (w/v) Bromophenol Blue. Eluted proteins were detected by immunoblotting.

### Immunoblotting

Cells in culture were washed three times with prechilled PBS and harvested with a rubber cell scraper. Cell pellets were resuspended in lysis buffer containing 50 mM Tris–HCl pH 7.4, 150 mM NaCl, 1% NP‐40, 0.5% sodium deoxycholate, and freshly added protease inhibitor cocktail (Roche) for 1 h on ice. The cell lysates were then centrifuged at 1889 *g* at 4°C for 10 min. The concentration of protein from each sample was measured with Bio‐Rad protein assay (Bio‐Rad, Hercules, CA), equalized, and eluted by boiling with 4× loading buffer. Proteins from either cell lysates or immunoprecipitations were separated by SDS–PAGE on 10% gels and transferred onto nitrocellulose membranes (GE Healthcare, Chicago, IL). Membranes were blocked for 30 min at room temperature in PBS containing 0.3% (v/v) Tween‐20 (PBST) and 5% dried milk and then incubated overnight at 4°C with diluted primary antibodies. Protein–antibody complexes were detected with HRP‐conjugated goat anti‐mouse‐IgG (Jackson Research Laboratories, Bar Harbor, ME) or donkey anti‐rabbit‐IgG (GE Healthcare) secondary antibodies for 1 h at room temperature, followed by enhanced chemiluminescence substrate (ThermoFisher Scientific). Immunoblot images were acquired by iBright Imaging Systems (Invitrogen).

### Ubiquitination assay

To measure level of CP110 ubiquitination during ciliogenesis, RPE‐1 cells were first serum‐starved in the presence of 10 μM MG132 for 4 h and then harvested and lysed with lysis buffer containing 150 mM NaCl, 50 mM HEPES‐KOH pH 7.2, 1 mM MgCl_2_, 1% Triton X‐100, and freshly added protease inhibitor cocktail (Roche) for 1 h at 4°C. Cell debris was cleared by centrifugation, and the supernatants were incubated with anti‐CP110 antibody overnight at 4°C. Protein G‐agarose beads (GE Healthcare) for 4 h at 4°C. Protein G‐agarose beads (GE Healthcare) were added to the lysate–antibody mix and left to rock at 4°C for 2 h. Samples were then washed three times, eluted, and subjected to immunoblotting. Anti‐CP110 antibody was used to reveal the amount of Immunoprecipitated CP110 from each sample, and anti‐ubiquitin antibody was used to detect ubiquitinated‐CP110.

### Immunofluorescence and microscopy imaging

Cells plated on coverslips were fixed in 100% Methanol at −20°C for 5 min, followed by three rinses with PBS buffer. For immunofluorescence staining, cells were first permeabilized in 0.5% Triton X plus 0.5% BSA in PBS for 30 min and then stained with appropriate primary antibodies diluted in PBS buffer containing 0.1% Triton X and 0.5% BSA for 1 h at room temperature. PBS washes were applied to remove unbound primary antibodies. Cells were then incubated with fluorochrome‐conjugated secondary antibodies for 1 h at room temperature and washed three more times in PBS. Coverslips were mounted in Fluoromount G Mounting medium (SouthernBiotech). Imaging was performed with a Zeiss LSM 800 confocal microscope (Carl Zeiss, Jena, Germany) using a Plan‐Apochromat 63×/1.4 NA oil objective and appropriate filters, as previously described. Image acquisition was carried out with Zen software (Carl Zeiss). Z‐slices were z‐projected with ImageJ (National Institutes of Health, Bethesda, MD). Images were cropped, and adjusted for brightness (whole‐image adjustment) with minimal manipulation for better presentation. Particle tracking analysis was done with Imaris 9.9.1 software (Oxford Instruments, UK) using an autoregressive motion tracking algorithm with appropriate threshold. For quantification, three independent experiments were carried out, and the number of samples collected for quantification is described in the text.

### Statistical analysis

Data obtained from ImageJ were exported to GraphPad Prism 7 (GraphPad, San Diego, CA). Bar graphs were created representing the mean and standard deviation from data obtained from three independent experiments. Normal distribution was assessed with the D'Agostino and Pearson normality test, unless sample size was too small, in which case we used the Shapiro–Wilk test for normality (method of Royston (Royston, 1995)). Statistical significance was calculated with an unpaired two‐tailed *t*‐test for normal distribution and with the Mann–Whitney nonparametric two‐tailed test for non‐normal distributions. No blinding was done in the experimentation.

### Live‐cell imaging

Mock‐ or EHD1‐siRNA‐treated RPE‐1 cells on 35 mm glass‐bottom tissue culture dishes (MatTek, Ashland, MA) were transiently transfected with PCM‐1‐GFP for 24 h, and then serum‐starved in DMEM/F12 with 2 mM L‐glutamine, 1× nonessential amino acids and 0.2% FBS for 15 min at 37°C. The cells were imaged in prewarmed Opti‐MEM medium (Gibco) containing 2 mM L‐glutamine with a Zeiss LSM 800 confocal microscope (Carl Zeiss, Jena, Germany). Images were obtained every 5 s for 10 min with the pinhole set to obtain z‐sections of 1 μm. Images and videos were cropped, adjusted for brightness (whole‐image adjustment), and time‐stamped with minimal manipulation for presentation. For quantification, we performed three independent experiments. pEGFP‐N‐PCM1 (Dammermann & Merdes, 2002) was a kind gift from Dr. Andreas Merdes (Université Toulouse III, Toulouse, France).

## Author contributions

**Shuwei Xie:** Conceptualization; formal analysis; validation; investigation; methodology; writing – original draft; writing – review and editing. **Naava Naslavsky:** Conceptualization; formal analysis; supervision; investigation; visualization. **Steve Caplan:** Conceptualization; formal analysis; supervision; funding acquisition; methodology; writing – original draft; project administration; writing – review and editing.

## Disclosure and competing interests statement

The authors declare that they have no conflict of interest.

## Supporting information



Expanded View Figures PDF

Movie EV1

Movie EV2

PDF+

Source Data for Figure 1

Source Data for Figure 2

Source Data for Figure 3

Source Data for Figure 4

Source Data for Figure 5

Source Data for Figure 6

## Data Availability

No data from this manuscript require deposition in a public database.
