# Supplementary material for: EHD1 promotes CP110 ubiquitination by centriolar satellite delivery of HERC2 to the mother centriole
Source: EMBO Rep. 2023 Apr 19;24(6):e56317. doi: 10.15252/embr.202256317 (PMC10240189; doi:10.15252/embr.202256317)
Supplement: Supplementary file 1 — Expanded View Figures PDF [file EMBR-24-e56317-s007.pdf]

Expanded View Figures

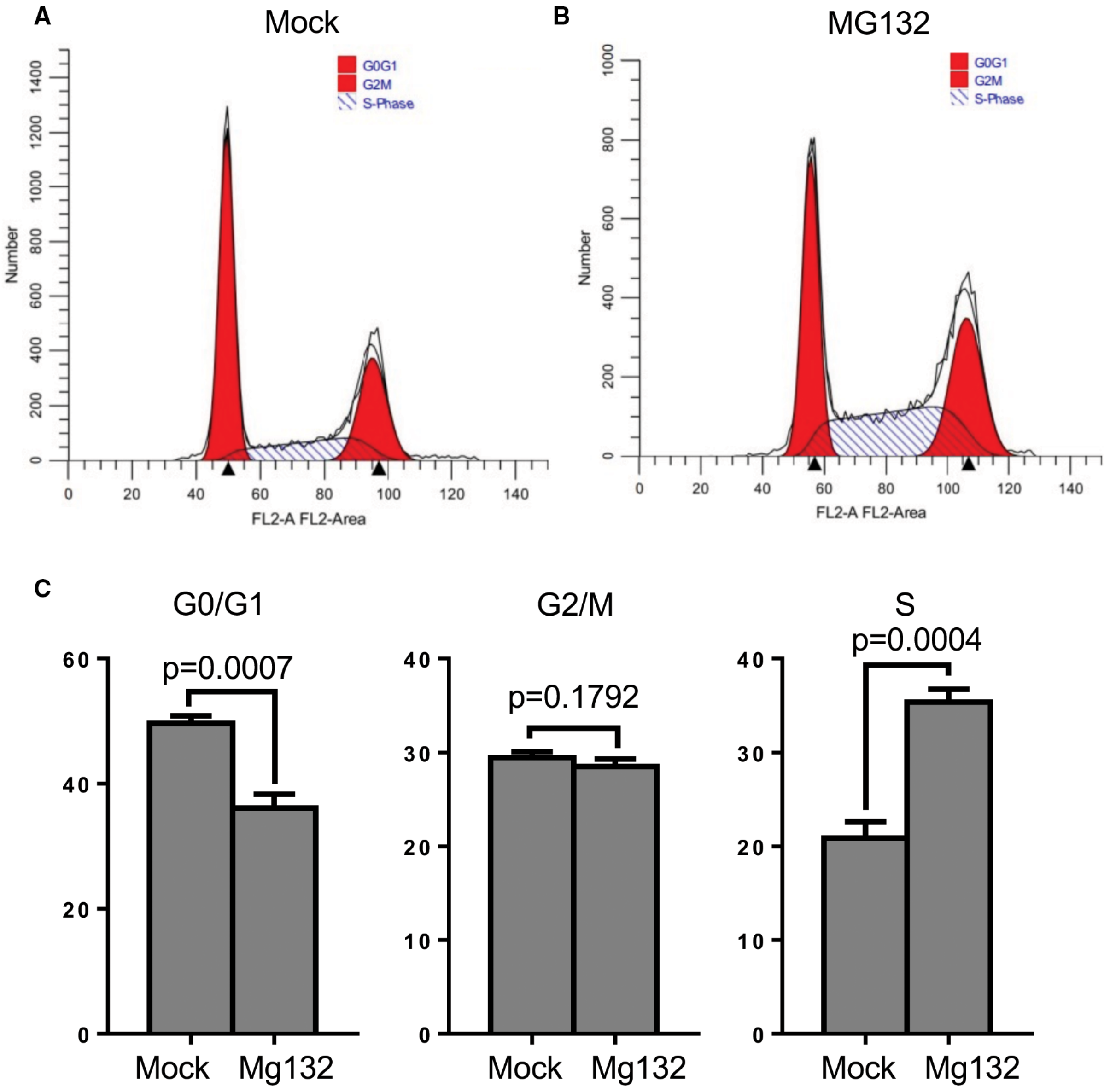

Figure EV1. Effect of MG132 on cell cycle.

A–C RPE-1 cells were treated with either DMSO (Mock-treated, A) or MG132 (B) for 4 h, fixed with 70% ethanol at 4°C, stained with Telford reagent at 4°C overnight, and subjected to a flow cytometry cell cycle assay. The percentage of cells in G1/G0, G2/M, and S phase are presented as bar graphs in (C). After normal distribution was determined by the Shapiro–Wilk normality test, statistical significance was calculated with an unpaired two-tailed *t*-test. Graphs show standard deviation and *P*-values from three independent experiments.

 © 2023 The Authors

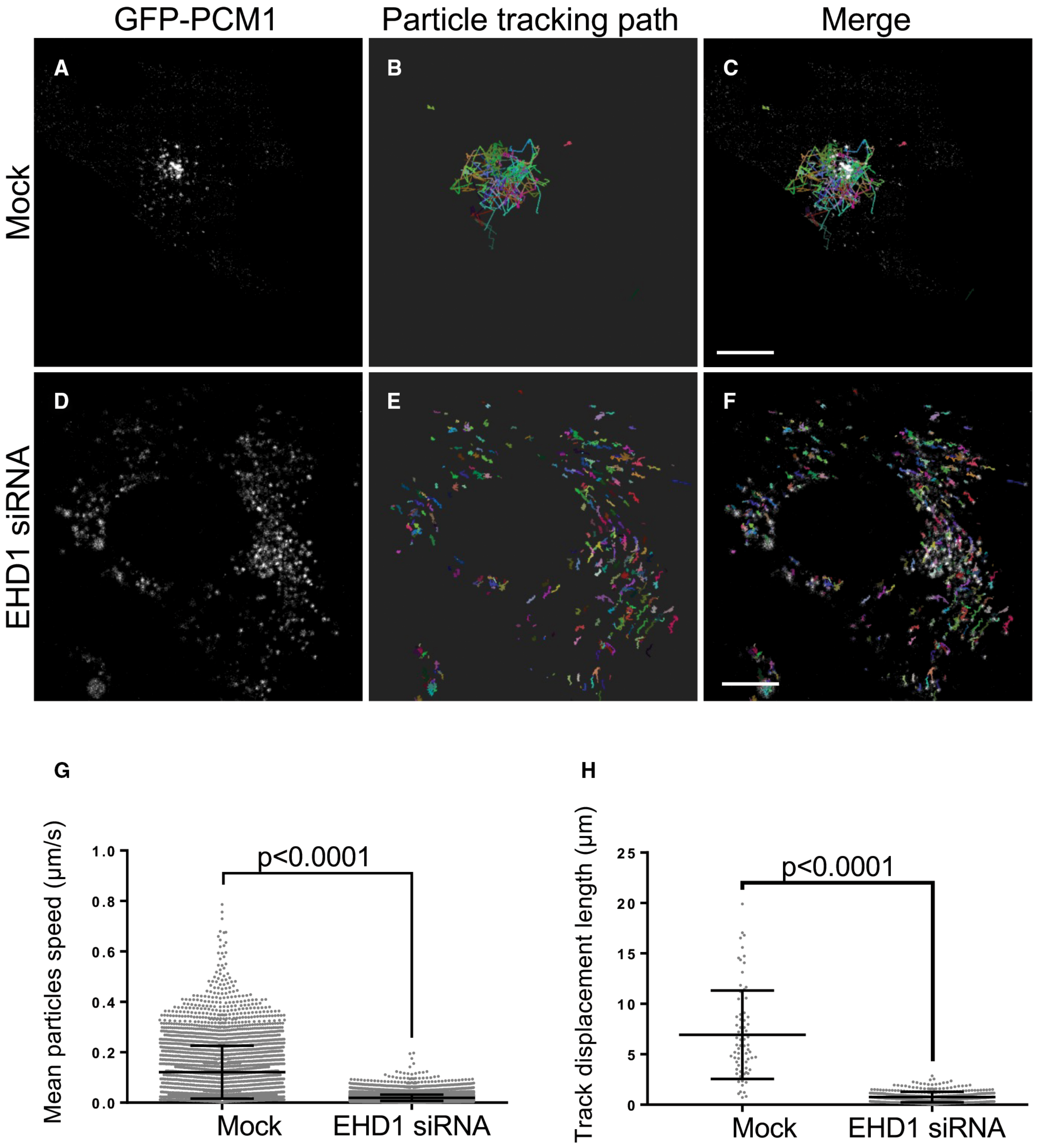
GFP-PCM1
Particle tracking path
Merge
A
B
C
Mock
D
E
F
EHD1 siRNA
G
H
Mean particles speed (μm/s)
1.0
0.8
0.6
0.4
0.2
0.0
p<0.0001
Mock
EHD1 siRNA
Track displacement length (μm)
25
20
15
10
5
0
p<0.0001
Mock
EHD1 siRNA

Figure EV2.

◀ **Figure EV2. Depletion of EHD1 impedes the distribution of centriolar satellites to the centrosomal region.**

A–F Mock- (A–C) or EHD-siRNA-treated (D–F) RPE-1 cells were transfected with PCM-1-GFP for 24 h and then serum-starved for 15 min. The distributions and dynamics of PCM-1 labeled centriolar satellites were monitored by live-cell imaging. Representative images showing the distributions of PCM-1-GFP in the mock- (A) or EHD1-siRNA- (D) treated cells are shown. Particle tracking analysis was done with Imaris 9.9.1 software (Oxford Instruments) using an autoregressive motion tracking algorithm with appropriate threshold. Full tracks marking the movement of each detected particle during live-cell imaging from mock-treated (B, merged in C) and EHD1-siRNA-treated (E, merged in F) RPE-1 cells are displayed. Each color represents the tracking path of an individual particle detected by the algorithm. Bars, 10 μm.

G Graph showing the mean particle speed over 10 min of live-cell imaging in mock- and EHD1-siRNA-treated RPE-1 cells. Assumption of normality was not met by the D'Agostino and Pearson normality test, and the Mann–Whitney test was used to determine statistical significance (*P*-values).

H Graph displaying the mean track displacement length during the 10 min live-cell imaging in mock- and EHD1-siRNA-treated RPE-1 cells. Assumption of normality was not met by the D'Agostino and Pearson normality test, and the Mann–Whitney test was used to determine statistical significance (*P*-values).

Data information: Graphs displaying standard deviation and *P*-values are from three individual biological experiments. More than 300 particles were detected, and their speeds were calculated and quantified for each experiment. At least 17 particle tracking paths were measured for each experiment. Statistical significance was calculated with the Mann–Whitney two-tailed test for samples that did not meet the assumption of normality.

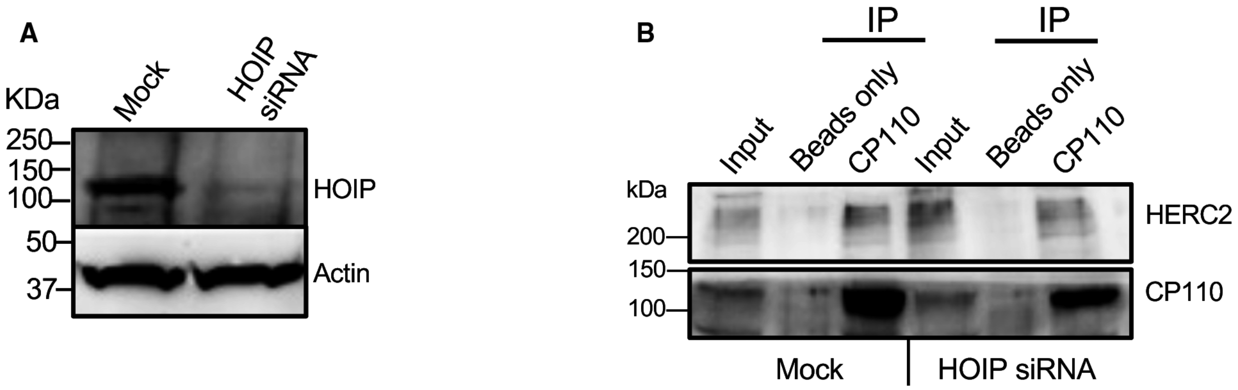

Figure EV3. Interaction between CP110 and HERC2 is independent of LUBAC component HOIP.

A Validation of HOIP depletion in RPE-1 cells by immunoblotting.

B RPE-1 cells following mock treatment or HOIP siRNA treatment were lysed and immunoprecipitated with beads-only control, or anti-CP110 antibodies, and subjected to SDS–PAGE and immunoblotting with antibodies directed against CP110 (lower panel) and HERC2 (upper panel). 7% of inputs are shown on the left lane of the gel. The presented gel is a representative of three individual experiments.

 © 2023 The Authors
