# Supplementary material for: EHD1 promotes CP110 ubiquitination by centriolar satellite delivery of HERC2 to the mother centriole
Source: EMBO Rep. 2023 Apr 19;24(6):e56317. doi: 10.15252/embr.202256317 (PMC10240189; doi:10.15252/embr.202256317)
Supplement: Supplementary file 3 — Movie EV2 [file EMBR-24-e56317-s003.zip › EMBOR-2022-56317V3-Movie_EV2/Movie_EV2- legend.docx]

Movie EV2. **Impaired** **PCM-1-GFP movement EHD1-depleted cells.** RPE-1 cells were EHD1-siRNA treated, serum starved for 15 min. and subjected to live-cell imaging as described in the Methods. The video shows representative dynamics of PCM-1-GFP in EHD1-siRNA-treated RPE-1 cells. This video is cropped and adjusted for brightness and contrast (whole-image adjustment), and time-stamped with minimal manipulation for presentation. The video is a representative one from 3 individual biological experiments.
