# Supplementary figures and images for: EHD1 promotes CP110 ubiquitination by centriolar satellite delivery of HERC2 to the mother centriole
Source: EMBO Rep. 2023 Apr 19;24(6):e56317. doi: 10.15252/embr.202256317 (PMC10240189; doi:10.15252/embr.202256317)

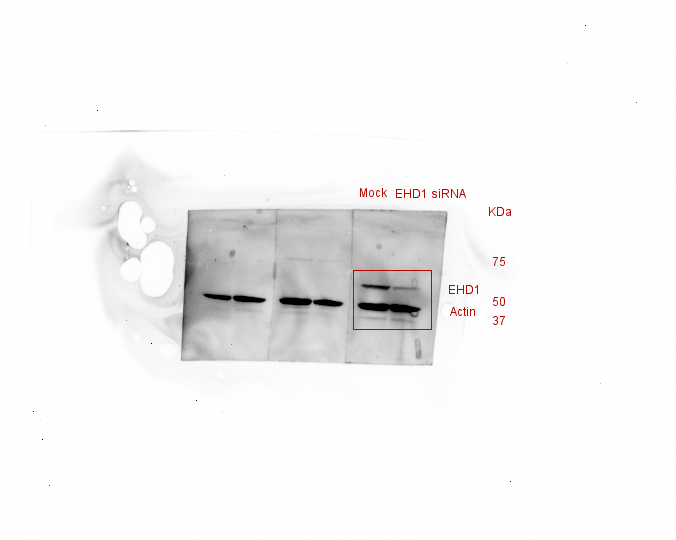

Supplement: Supplementary file 5 — Source Data for Figure 1 [file EMBR-24-e56317-s002.zip › Figure 1/1H/Western EHD1siRNA.tif]

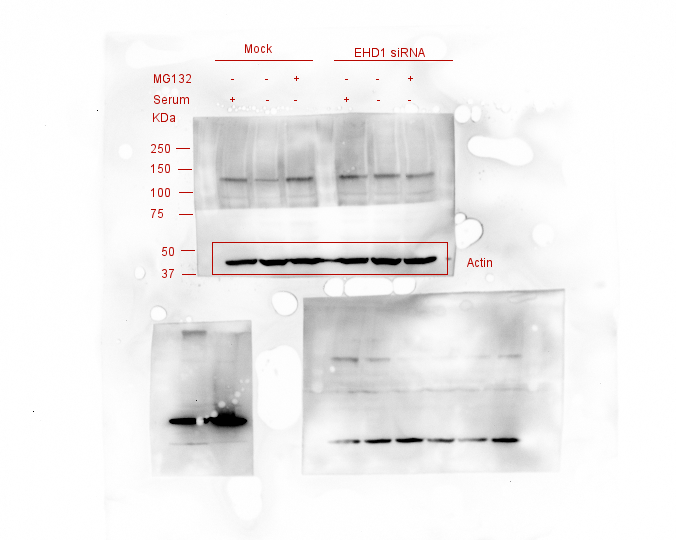

Supplement: Supplementary file 5 — Source Data for Figure 1 [file EMBR-24-e56317-s002.zip › Figure 1/1H/Western Actin.tif]

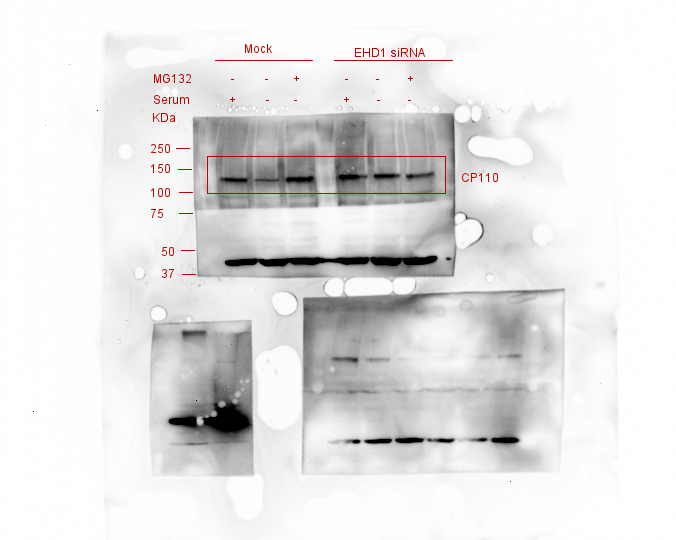

Supplement: Supplementary file 5 — Source Data for Figure 1 [file EMBR-24-e56317-s002.zip › Figure 1/1H/Western CP110.tif]

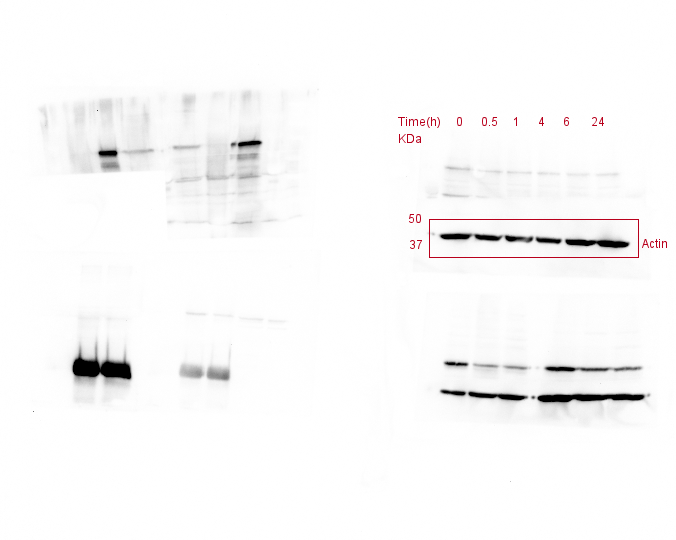

Supplement: Supplementary file 5 — Source Data for Figure 1 [file EMBR-24-e56317-s002.zip › Figure 1/1A/Western Actin.tif]

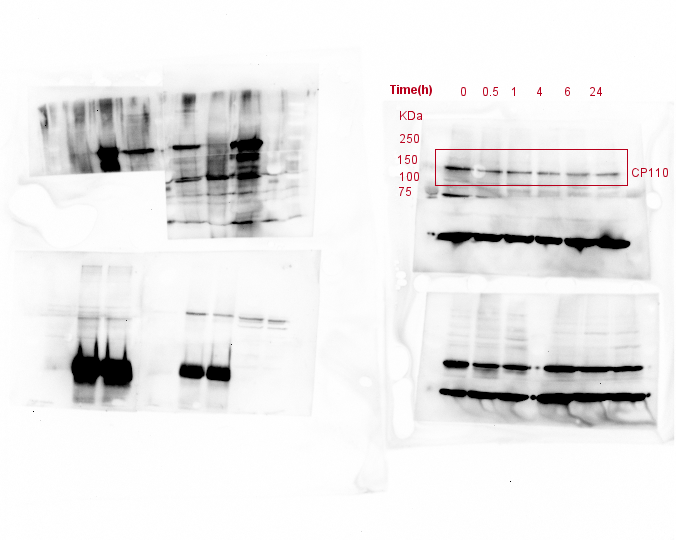

Supplement: Supplementary file 5 — Source Data for Figure 1 [file EMBR-24-e56317-s002.zip › Figure 1/1A/Western CP110.tif]

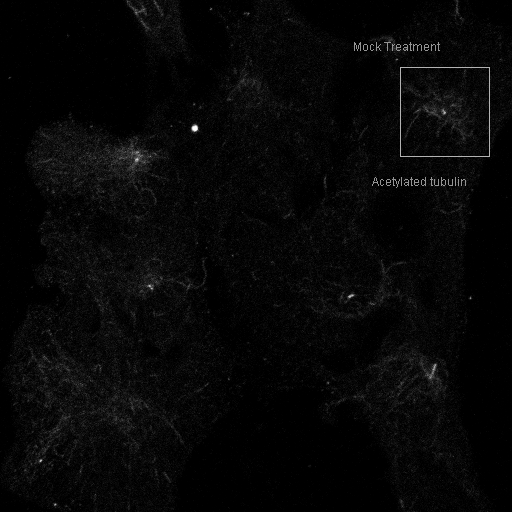

Supplement: Supplementary file 5 — Source Data for Figure 1 [file EMBR-24-e56317-s002.zip › Figure 1/1E/Acetylated tubulin-Mock treatment.tif]

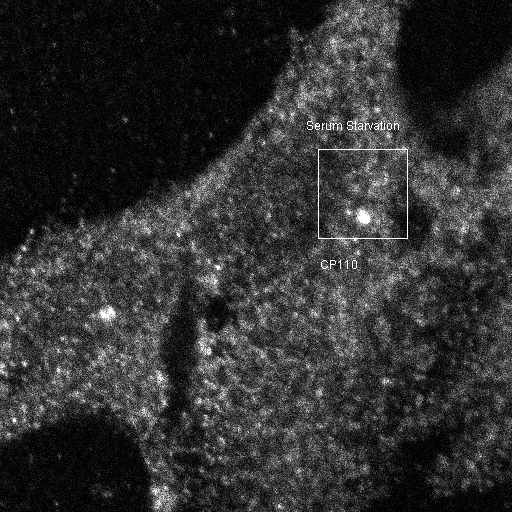

Supplement: Supplementary file 5 — Source Data for Figure 1 [file EMBR-24-e56317-s002.zip › Figure 1/1E/CP110-Serum Starvation.tif]

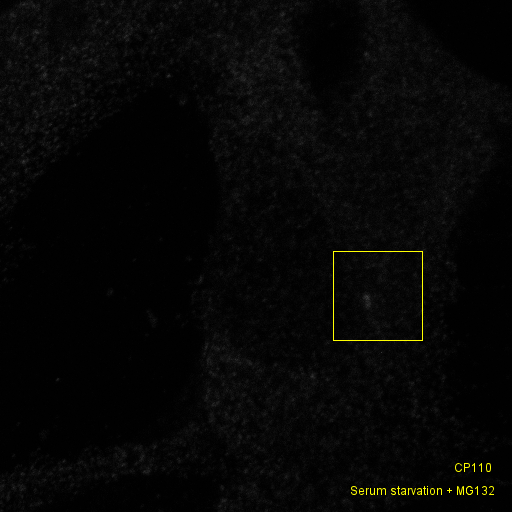

Supplement: Supplementary file 5 — Source Data for Figure 1 [file EMBR-24-e56317-s002.zip › Figure 1/1E/CP110-Serum Starvation+MG132.tif]

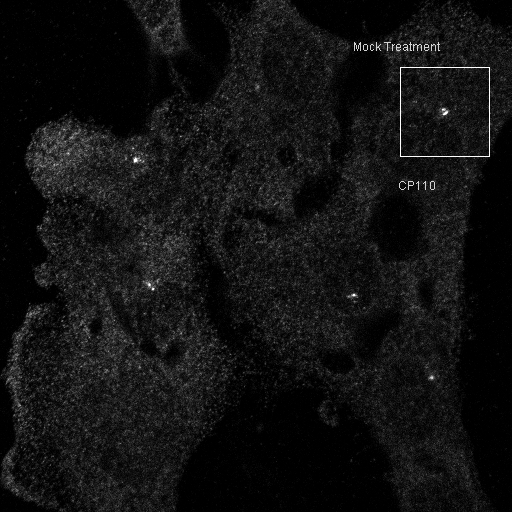

Supplement: Supplementary file 5 — Source Data for Figure 1 [file EMBR-24-e56317-s002.zip › Figure 1/1E/CP110-Mock treatment.tif]

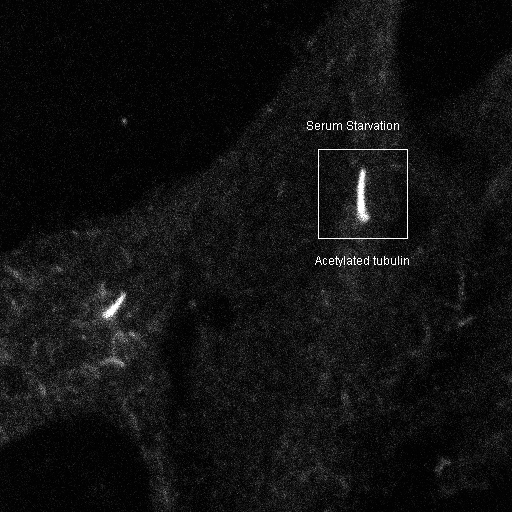

Supplement: Supplementary file 5 — Source Data for Figure 1 [file EMBR-24-e56317-s002.zip › Figure 1/1E/Acetylated tubulin-Serum Starvation.tif]

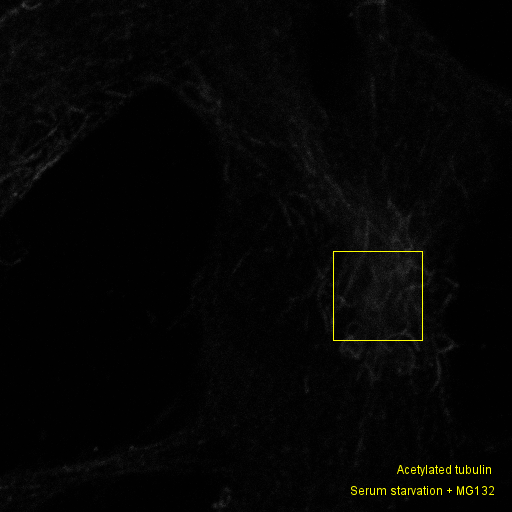

Supplement: Supplementary file 5 — Source Data for Figure 1 [file EMBR-24-e56317-s002.zip › Figure 1/1E/Acetylated tubulin-Serum Starvation+MG132.tif]

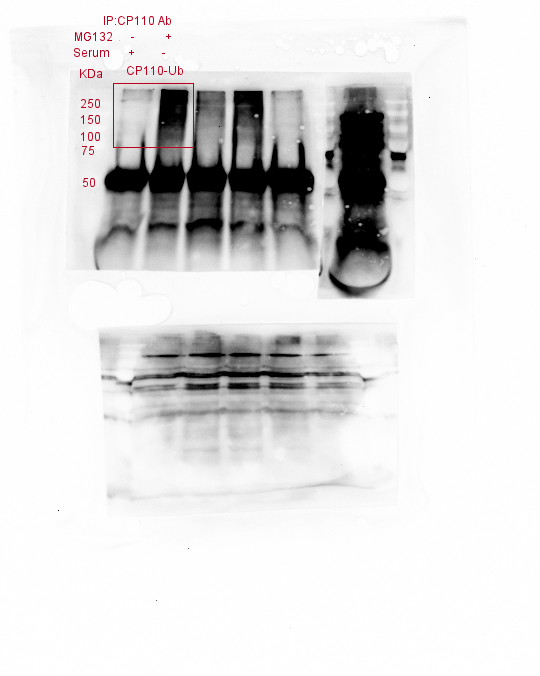

Supplement: Supplementary file 5 — Source Data for Figure 1 [file EMBR-24-e56317-s002.zip › Figure 1/1C/Western CP110-Ub.tif]

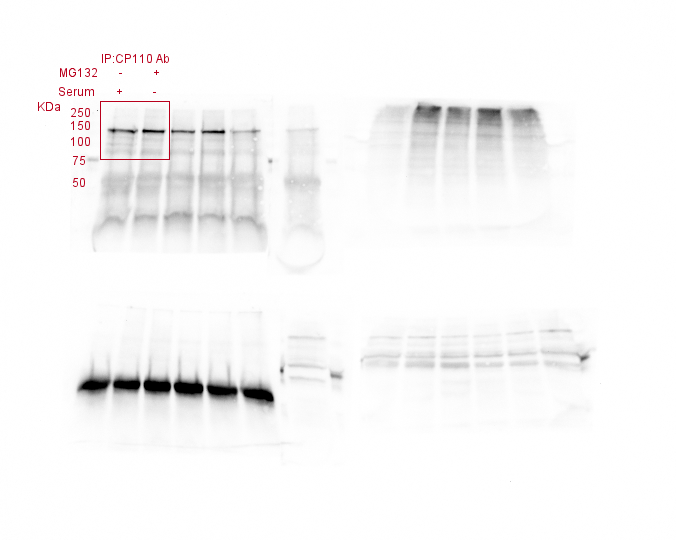

Supplement: Supplementary file 5 — Source Data for Figure 1 [file EMBR-24-e56317-s002.zip › Figure 1/1C/Western CP110 input.tif]

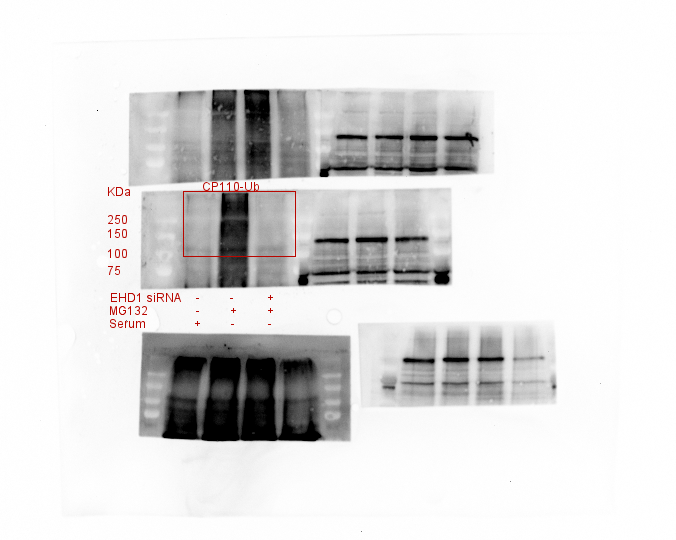

Supplement: Supplementary file 5 — Source Data for Figure 1 [file EMBR-24-e56317-s002.zip › Figure 1/1J/Western CP110-Ub upon EHD1siRNA.tif]

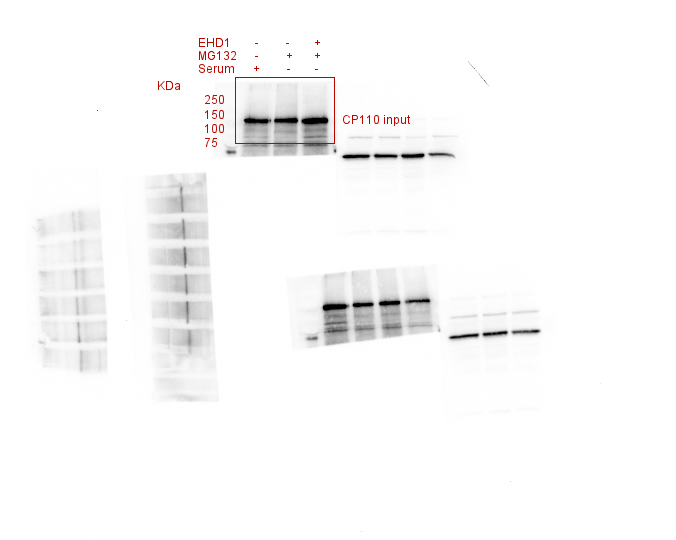

Supplement: Supplementary file 5 — Source Data for Figure 1 [file EMBR-24-e56317-s002.zip › Figure 1/1J/Western CP110 input.tif]

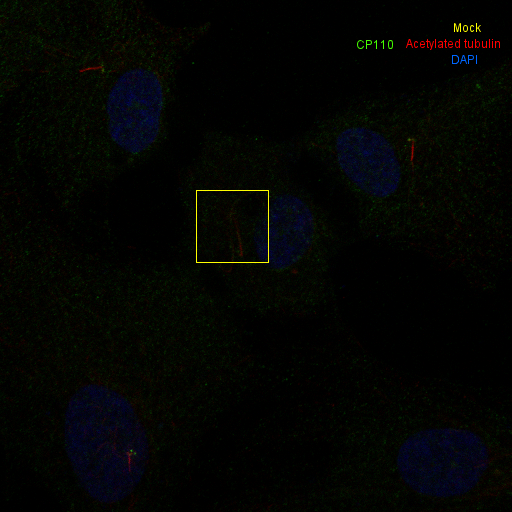

Supplement: Supplementary file 6 — Source Data for Figure 2 [file EMBR-24-e56317-s006.zip › Figure 2/2G/Micr.image Mock inset.tif]

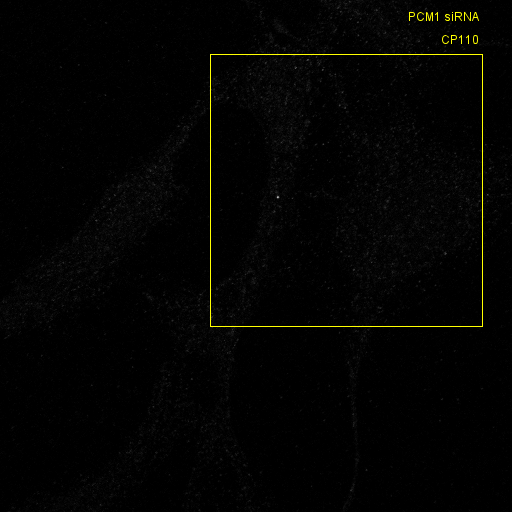

Supplement: Supplementary file 6 — Source Data for Figure 2 [file EMBR-24-e56317-s006.zip › Figure 2/2I/Micr.image PCM1 siRNA CP110.tif]

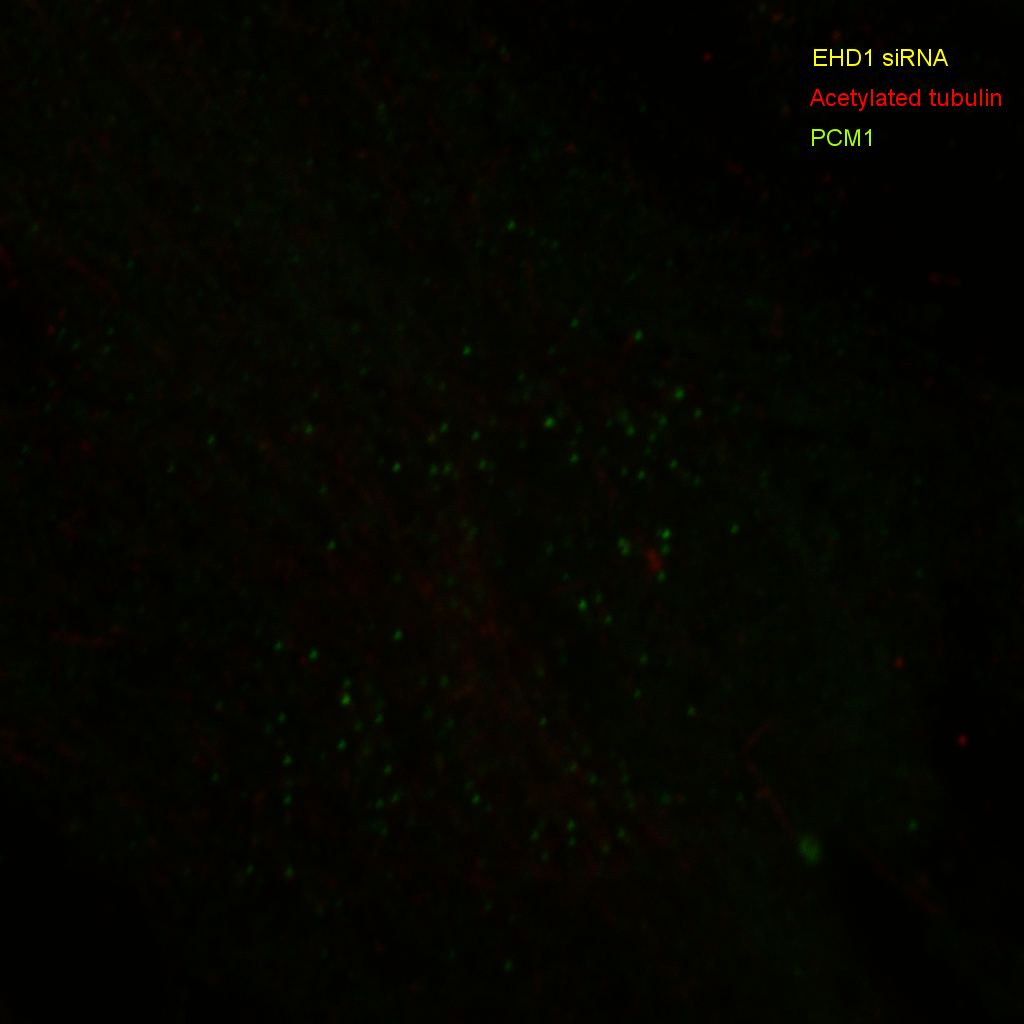

Supplement: Supplementary file 6 — Source Data for Figure 2 [file EMBR-24-e56317-s006.zip › Figure 2/2N/Micr.image EHD1 siRNA.tif]

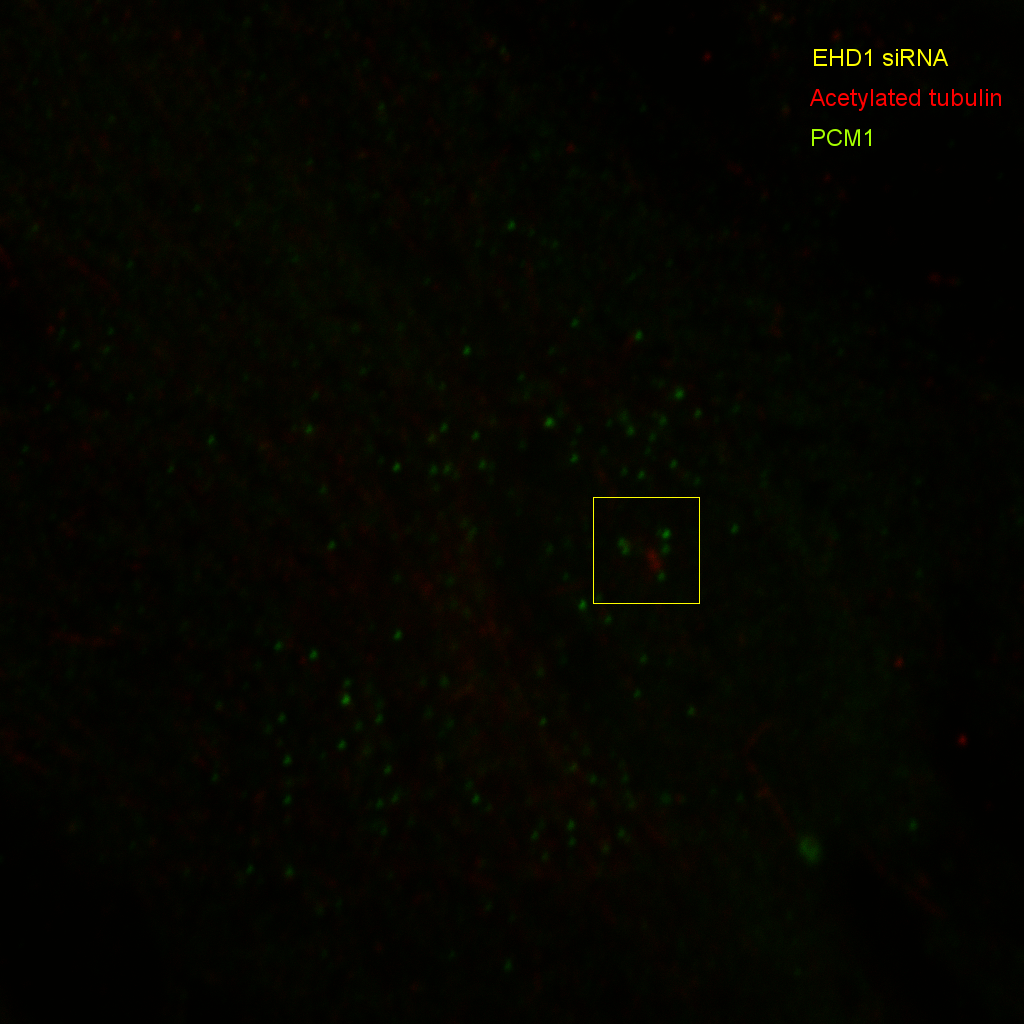

Supplement: Supplementary file 6 — Source Data for Figure 2 [file EMBR-24-e56317-s006.zip › Figure 2/2O/Micr.image EHD1 siRNA inset.tif]

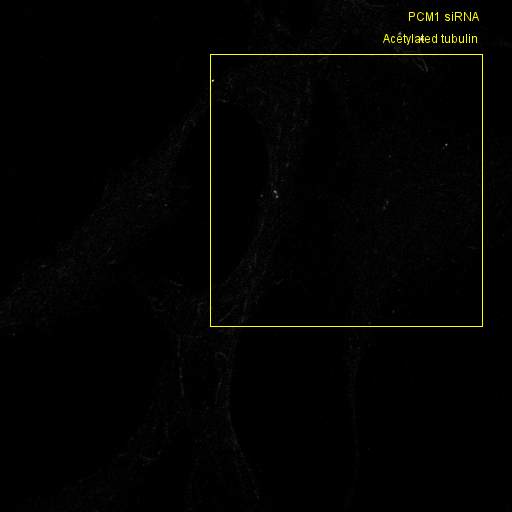

Supplement: Supplementary file 6 — Source Data for Figure 2 [file EMBR-24-e56317-s006.zip › Figure 2/2H/Micr.image PCM1 siRNA acetylated tubulin.tif]

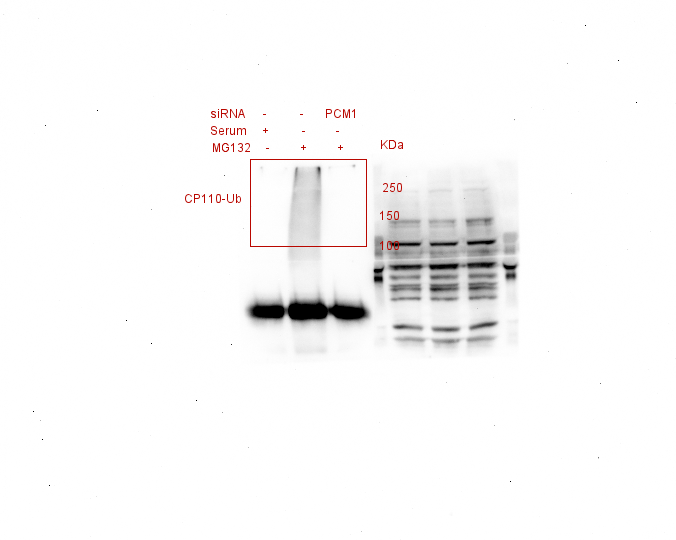

Supplement: Supplementary file 6 — Source Data for Figure 2 [file EMBR-24-e56317-s006.zip › Figure 2/2A/Western CP110-Ub.tif]

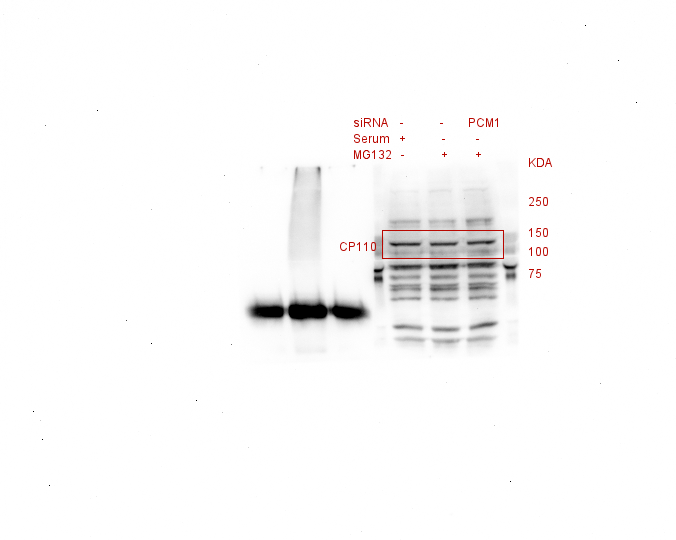

Supplement: Supplementary file 6 — Source Data for Figure 2 [file EMBR-24-e56317-s006.zip › Figure 2/2A/Western CP110input.tif]

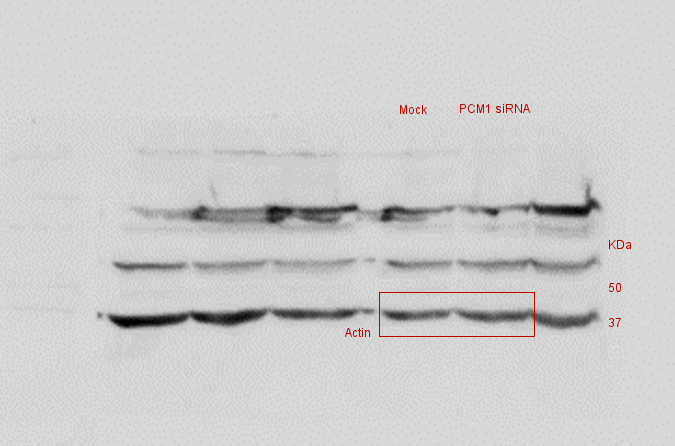

Supplement: Supplementary file 6 — Source Data for Figure 2 [file EMBR-24-e56317-s006.zip › Figure 2/2A/Actin.tif]

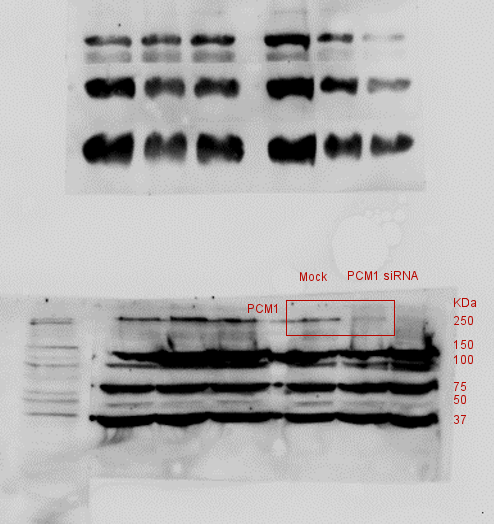

Supplement: Supplementary file 6 — Source Data for Figure 2 [file EMBR-24-e56317-s006.zip › Figure 2/2A/Western PCM1.tif]

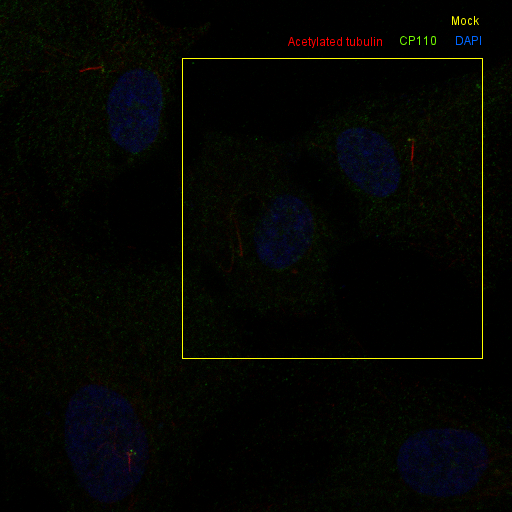

Supplement: Supplementary file 6 — Source Data for Figure 2 [file EMBR-24-e56317-s006.zip › Figure 2/2F/Micr.image Mock.tif]

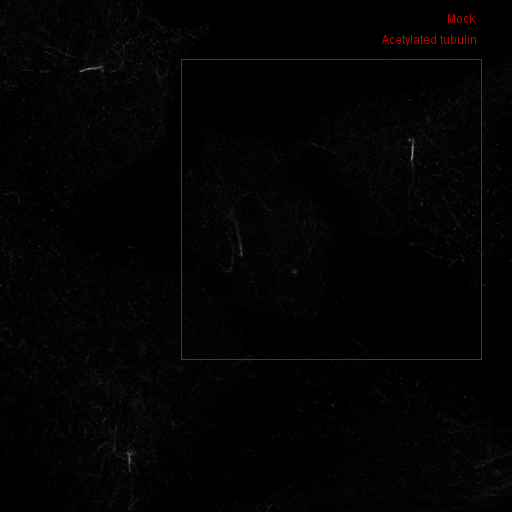

Supplement: Supplementary file 6 — Source Data for Figure 2 [file EMBR-24-e56317-s006.zip › Figure 2/2D/Micr.image Mock acetylated tubulin.tif]

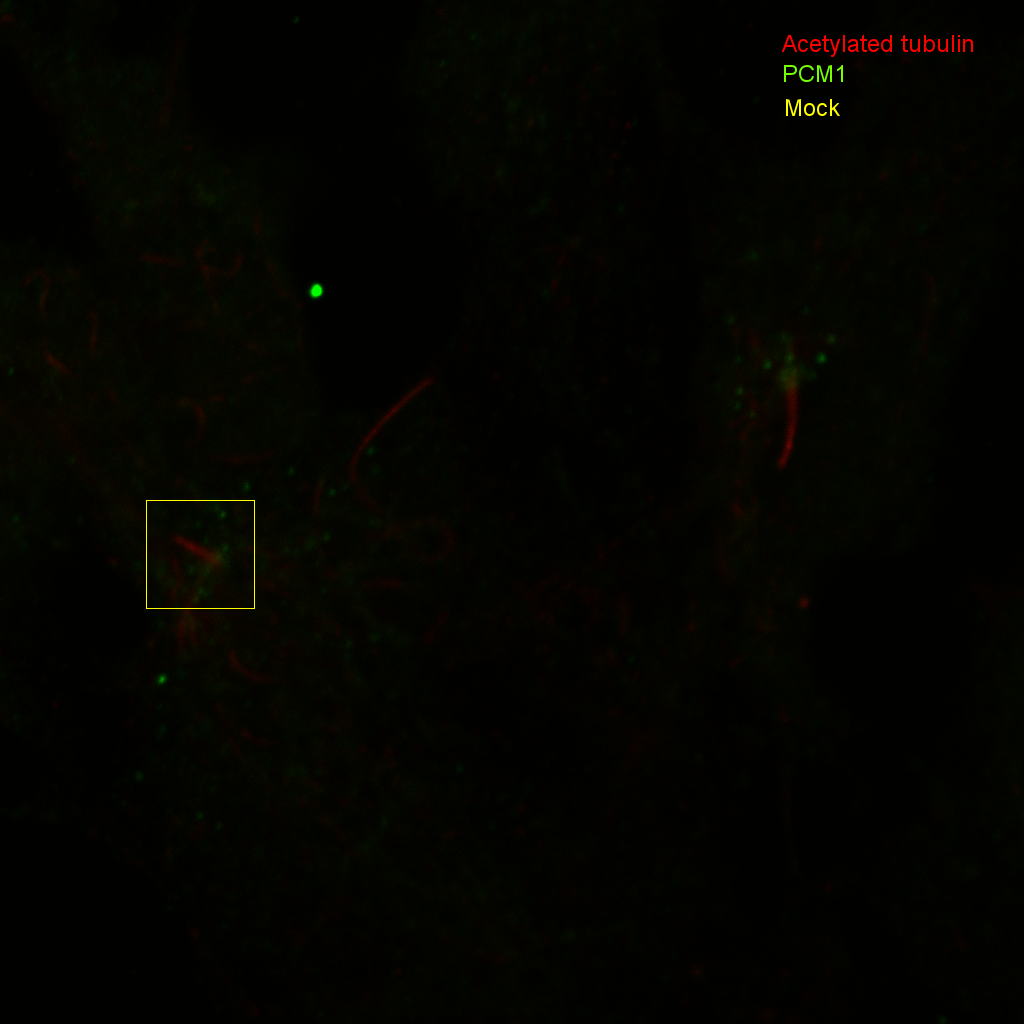

Supplement: Supplementary file 6 — Source Data for Figure 2 [file EMBR-24-e56317-s006.zip › Figure 2/2M/Micr.image Mock inset.tif]

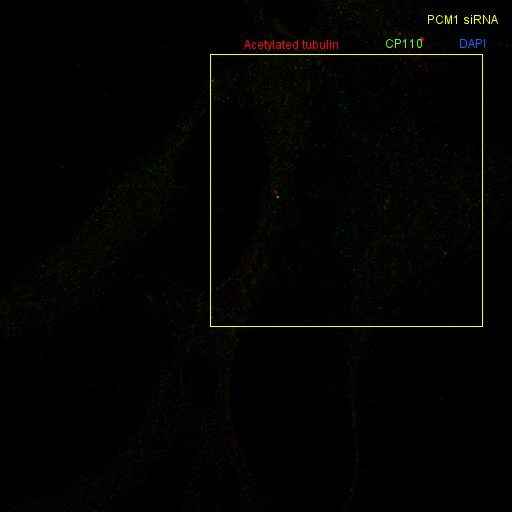

Supplement: Supplementary file 6 — Source Data for Figure 2 [file EMBR-24-e56317-s006.zip › Figure 2/2J/Micr.image PCM1 siRNA merge.tif]

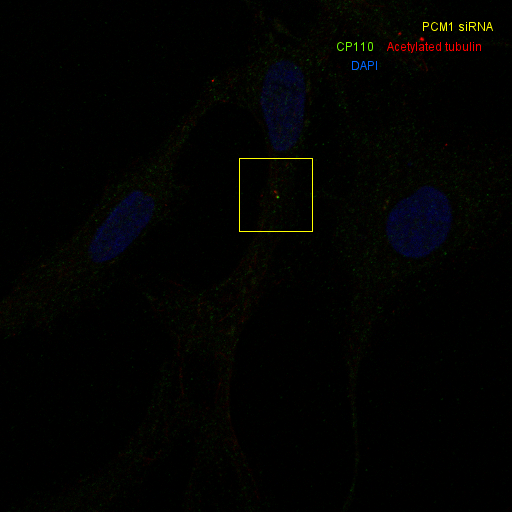

Supplement: Supplementary file 6 — Source Data for Figure 2 [file EMBR-24-e56317-s006.zip › Figure 2/2K/Micr.image PCM1 siRNA inset.tif]

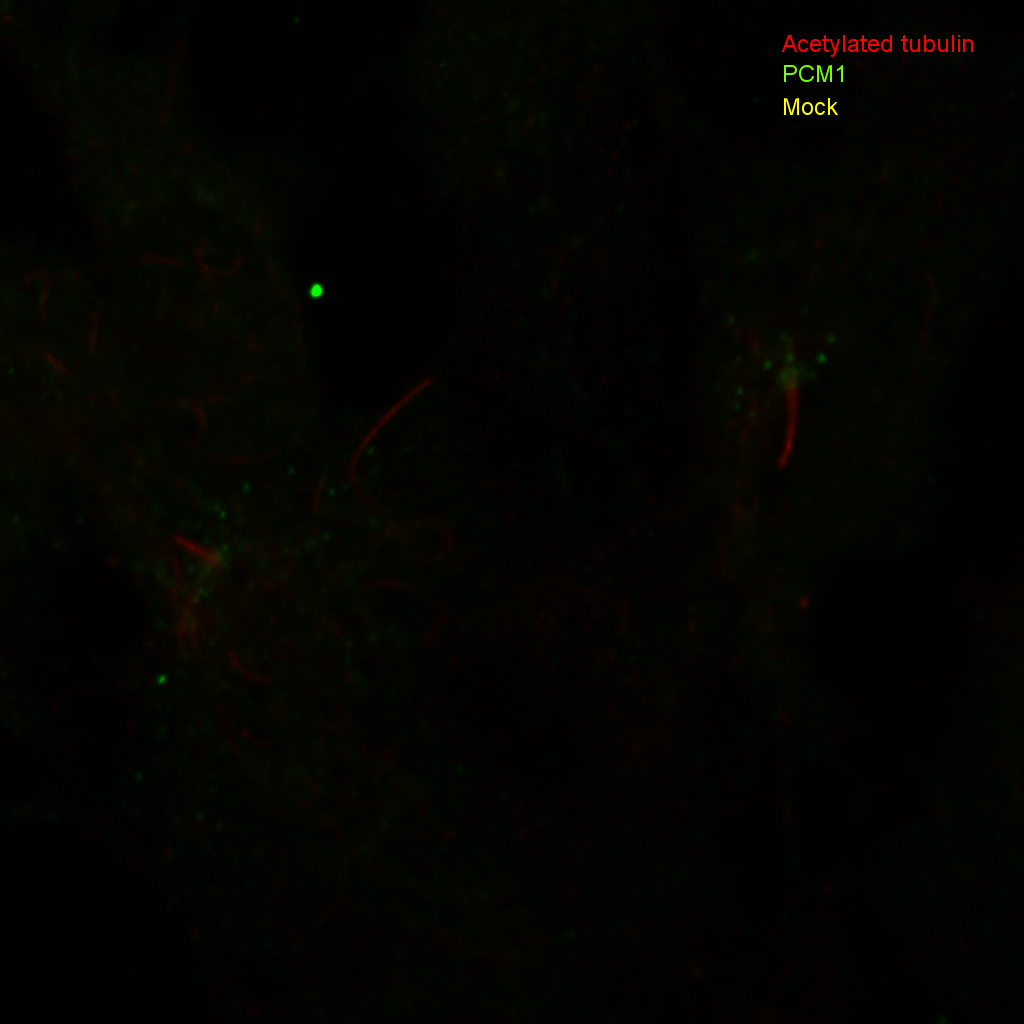

Supplement: Supplementary file 6 — Source Data for Figure 2 [file EMBR-24-e56317-s006.zip › Figure 2/2L/Micr.image Mock.tif]

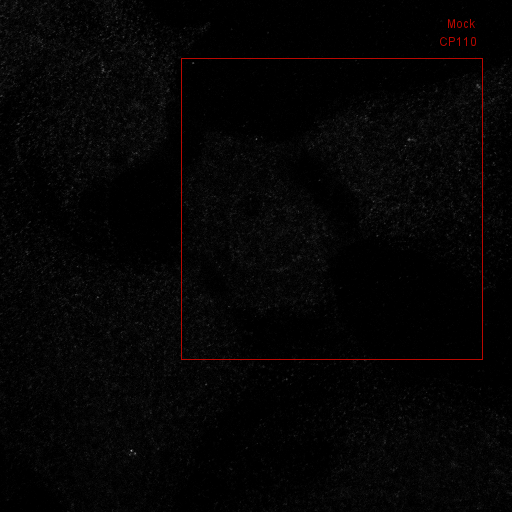

Supplement: Supplementary file 6 — Source Data for Figure 2 [file EMBR-24-e56317-s006.zip › Figure 2/2E/Micr.image Mock CP110.tif]

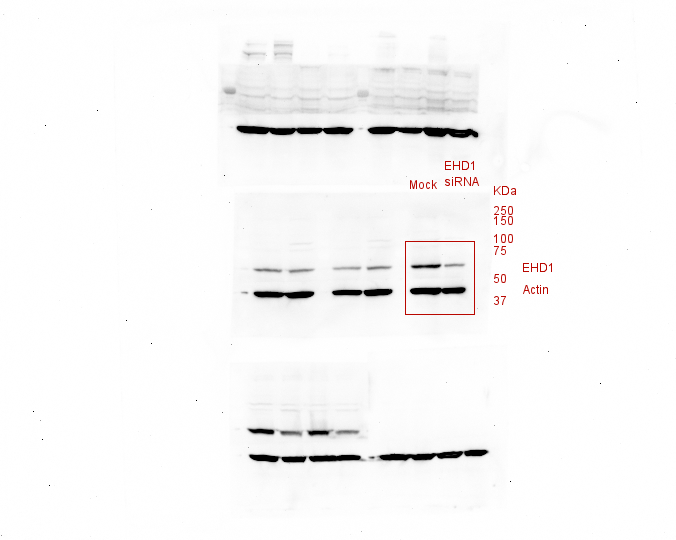

Supplement: Supplementary file 6 — Source Data for Figure 2 [file EMBR-24-e56317-s006.zip › Figure 2/2P/Western EHD1 siRNA + Actin.tif]

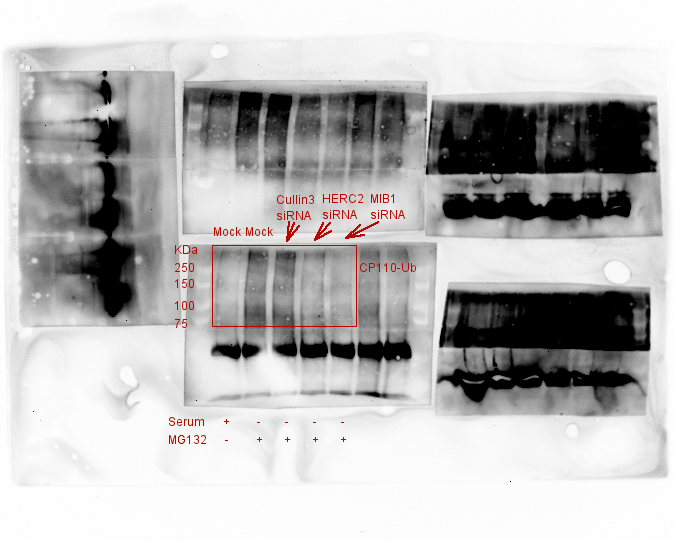

Supplement: Supplementary file 7 — Source Data for Figure 3 [file EMBR-24-e56317-s009.zip › Figure 3/3B/Western CP110-Ub.tif]

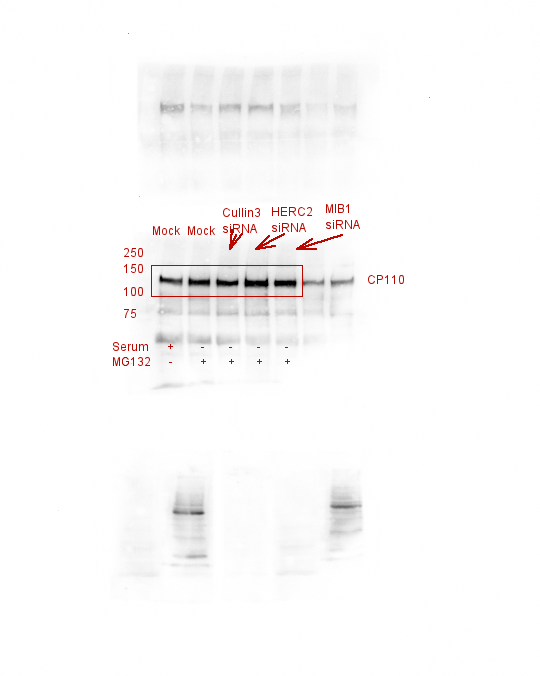

Supplement: Supplementary file 7 — Source Data for Figure 3 [file EMBR-24-e56317-s009.zip › Figure 3/3B/Wetstern CP110 input.tif]

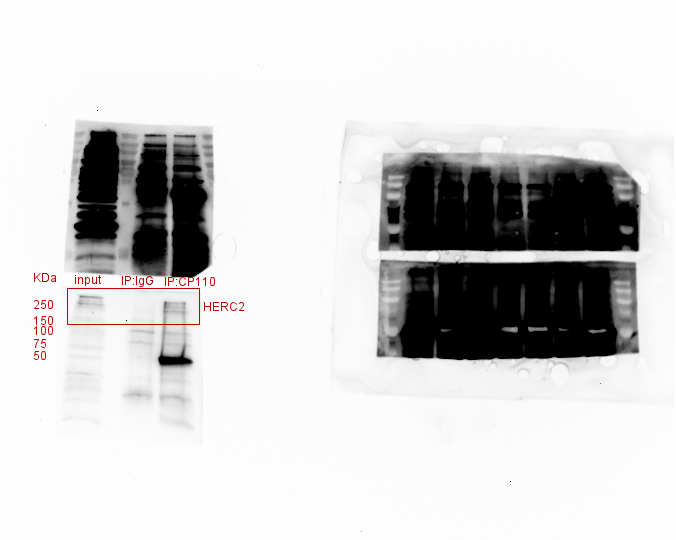

Supplement: Supplementary file 7 — Source Data for Figure 3 [file EMBR-24-e56317-s009.zip › Figure 3/3D/Western IP between HERC2 and CP110- HERC2.tif]

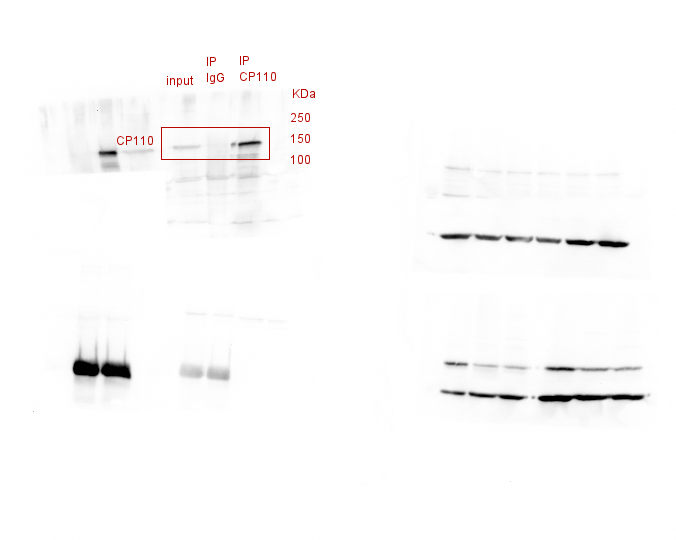

Supplement: Supplementary file 7 — Source Data for Figure 3 [file EMBR-24-e56317-s009.zip › Figure 3/3D/Western IP between Cullin3 and CP110- CP110.tif]

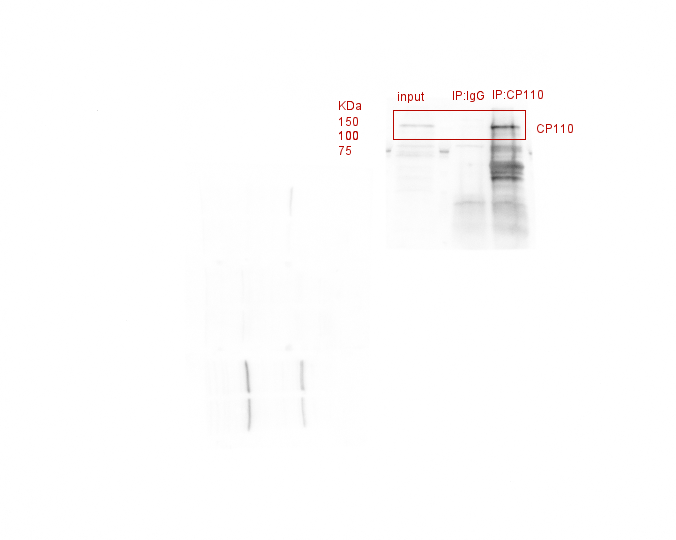

Supplement: Supplementary file 7 — Source Data for Figure 3 [file EMBR-24-e56317-s009.zip › Figure 3/3D/Western IP between HERC2 and CP110- CP110.tif]

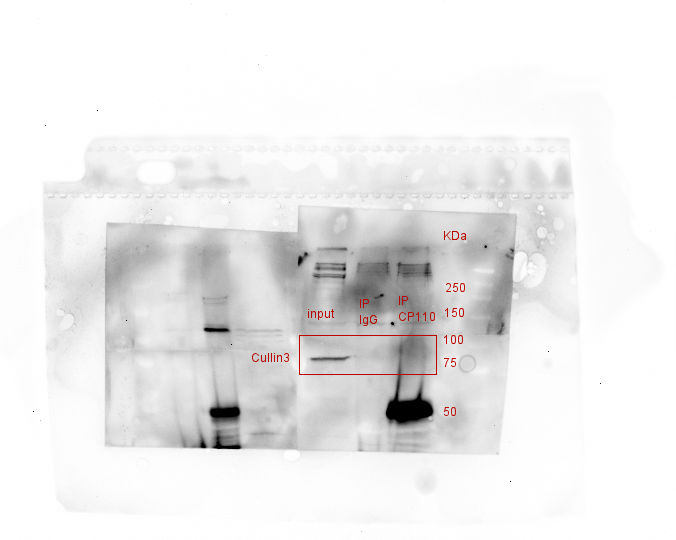

Supplement: Supplementary file 7 — Source Data for Figure 3 [file EMBR-24-e56317-s009.zip › Figure 3/3D/Western IP between Cullin3 and CP110- Cullin3.tif]

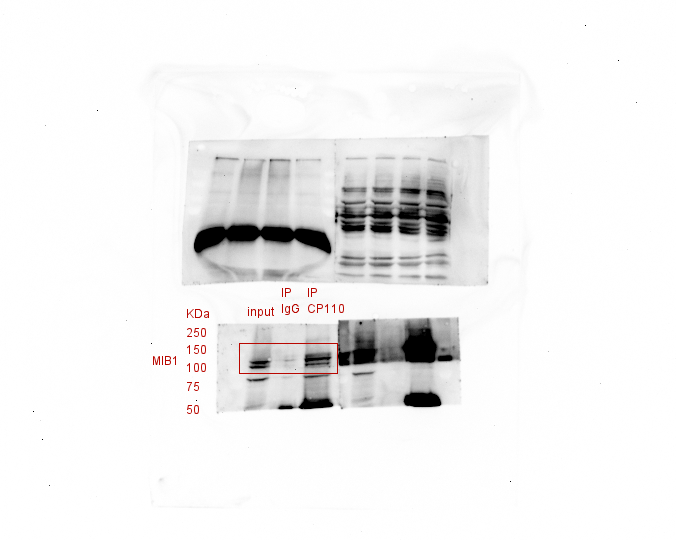

Supplement: Supplementary file 7 — Source Data for Figure 3 [file EMBR-24-e56317-s009.zip › Figure 3/3D/Western IP between MIB1 and CP110- MIB1.tif]

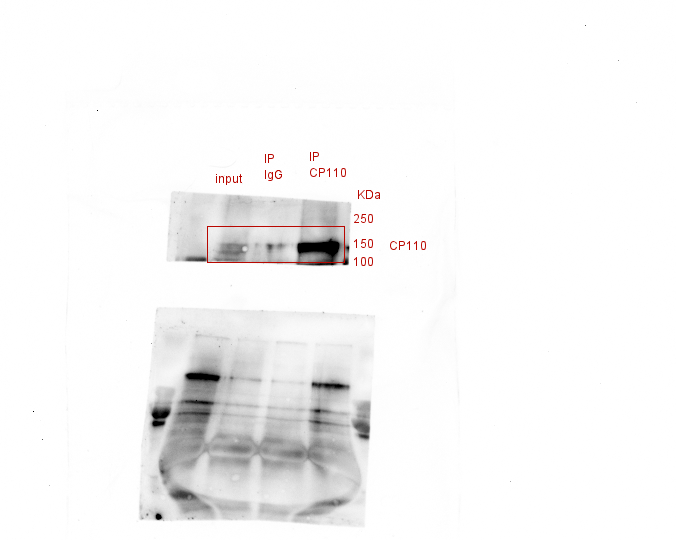

Supplement: Supplementary file 7 — Source Data for Figure 3 [file EMBR-24-e56317-s009.zip › Figure 3/3D/Western IP between MIB1 and CP110- CP110.tif]

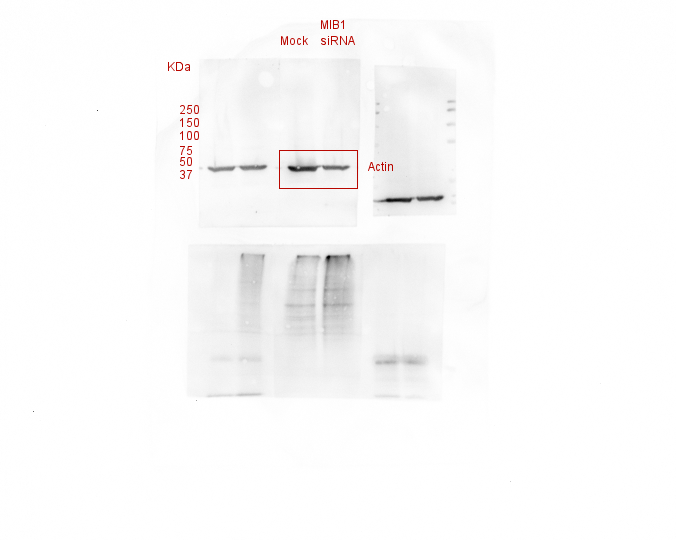

Supplement: Supplementary file 7 — Source Data for Figure 3 [file EMBR-24-e56317-s009.zip › Figure 3/3A/Western MIB1 siRNA actin.tif]

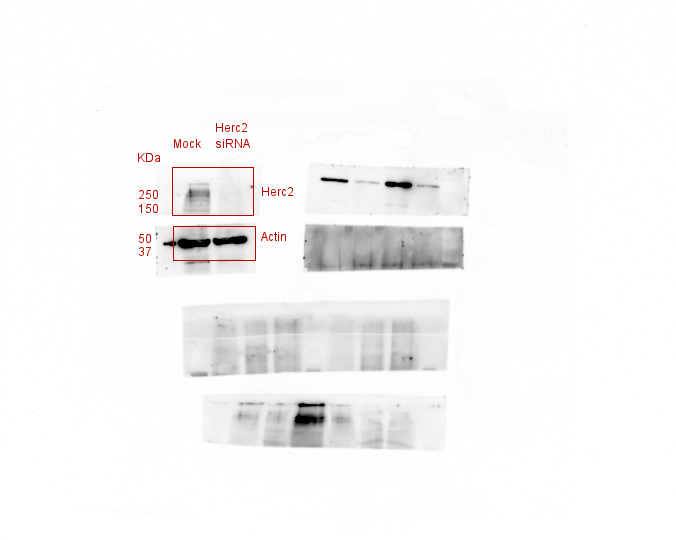

Supplement: Supplementary file 7 — Source Data for Figure 3 [file EMBR-24-e56317-s009.zip › Figure 3/3A/Western Herc2 siRNA + Actin.tif]

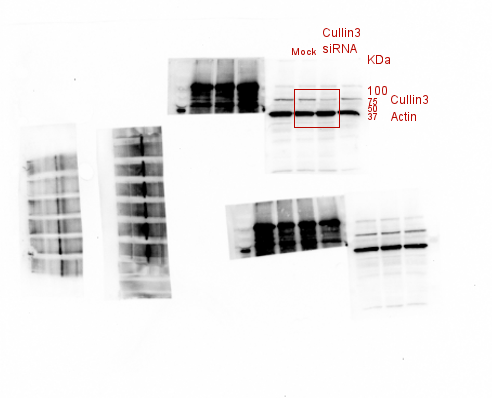

Supplement: Supplementary file 7 — Source Data for Figure 3 [file EMBR-24-e56317-s009.zip › Figure 3/3A/Western Cullin3 siRNA +Actin.tif]

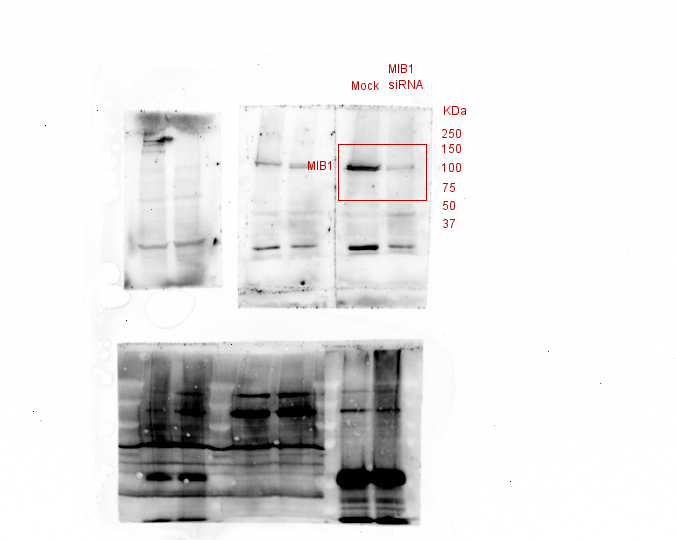

Supplement: Supplementary file 7 — Source Data for Figure 3 [file EMBR-24-e56317-s009.zip › Figure 3/3A/Western MIB1 siRNA MIB1.tif]

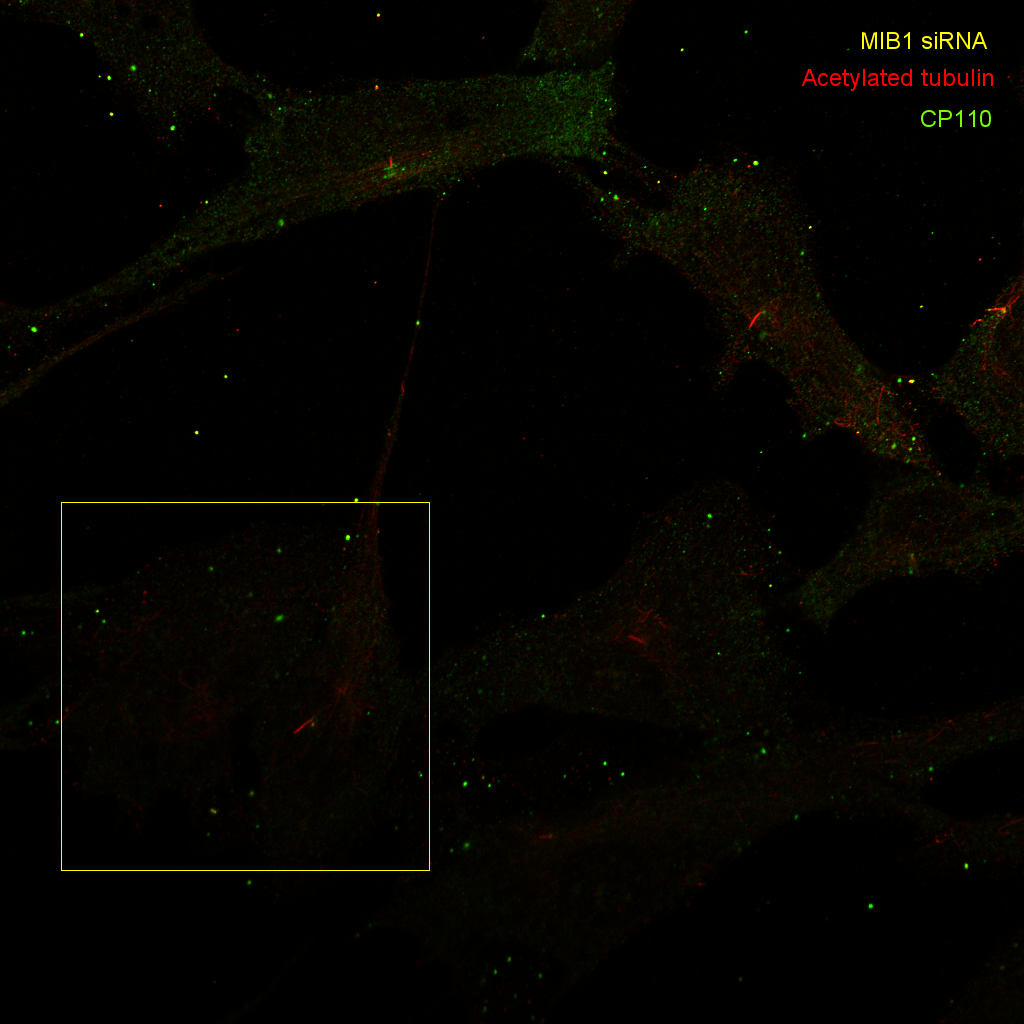

Supplement: Supplementary file 8 — Source Data for Figure 4 [file EMBR-24-e56317-s008.zip › Figure 4/4E/Micr.image MIB1 siRNA.tif]

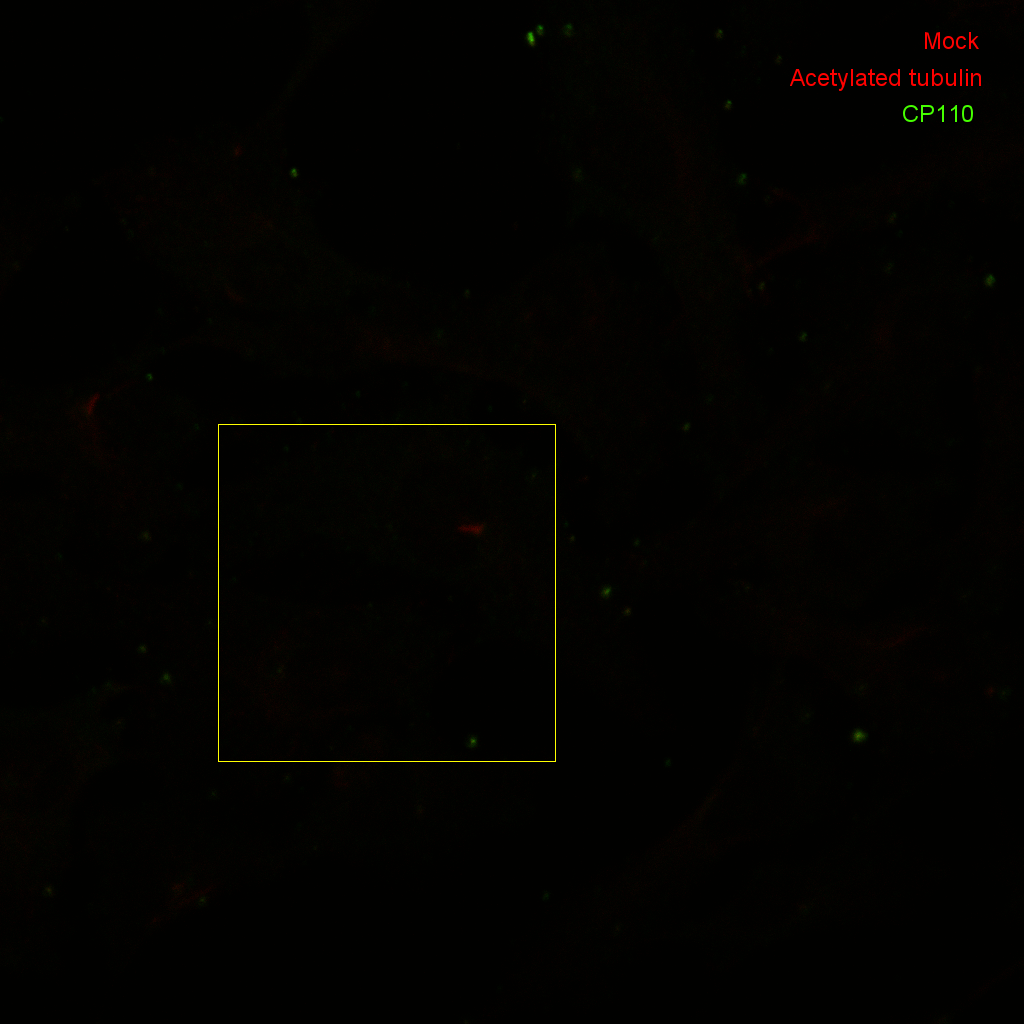

Supplement: Supplementary file 8 — Source Data for Figure 4 [file EMBR-24-e56317-s008.zip › Figure 4/4B/Micr.image Mock.tif]

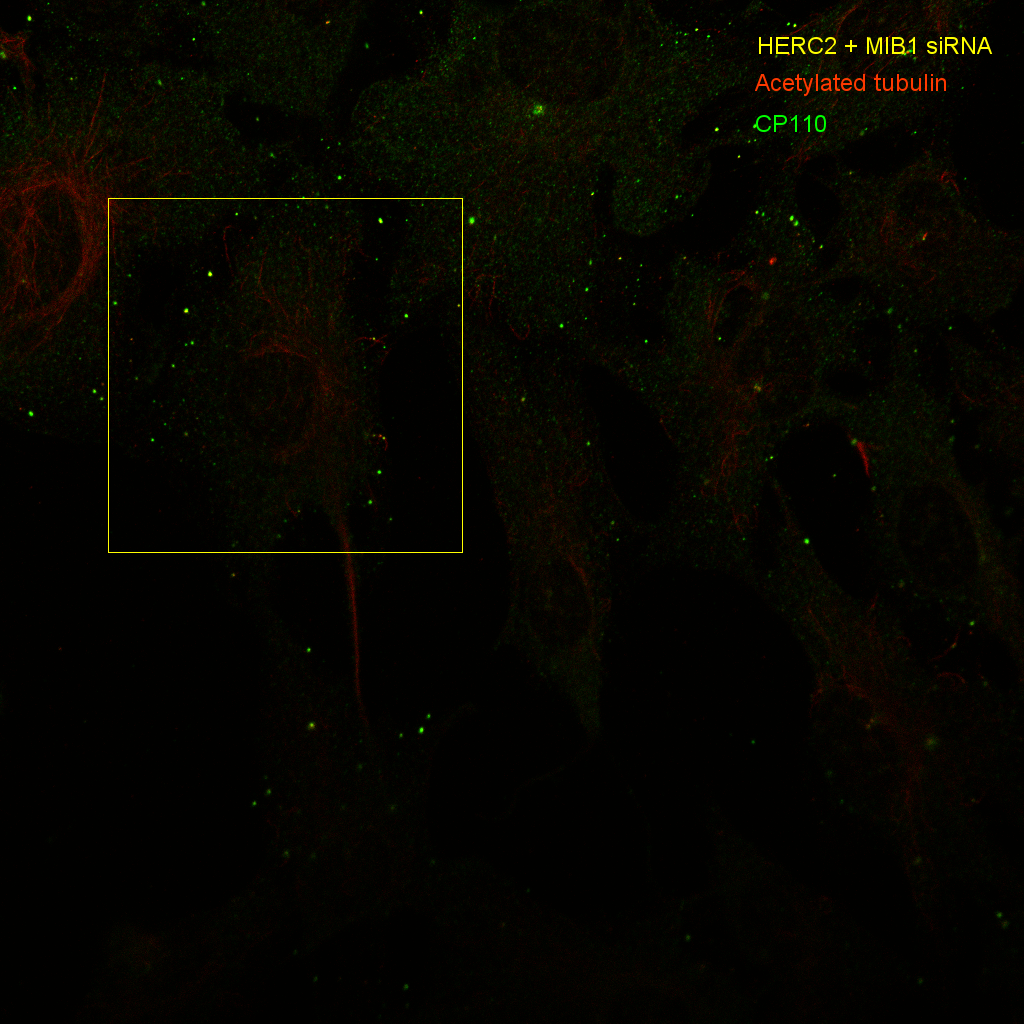

Supplement: Supplementary file 8 — Source Data for Figure 4 [file EMBR-24-e56317-s008.zip › Figure 4/4K/Micr.image HERC2+MIB1 siRNA.tif]

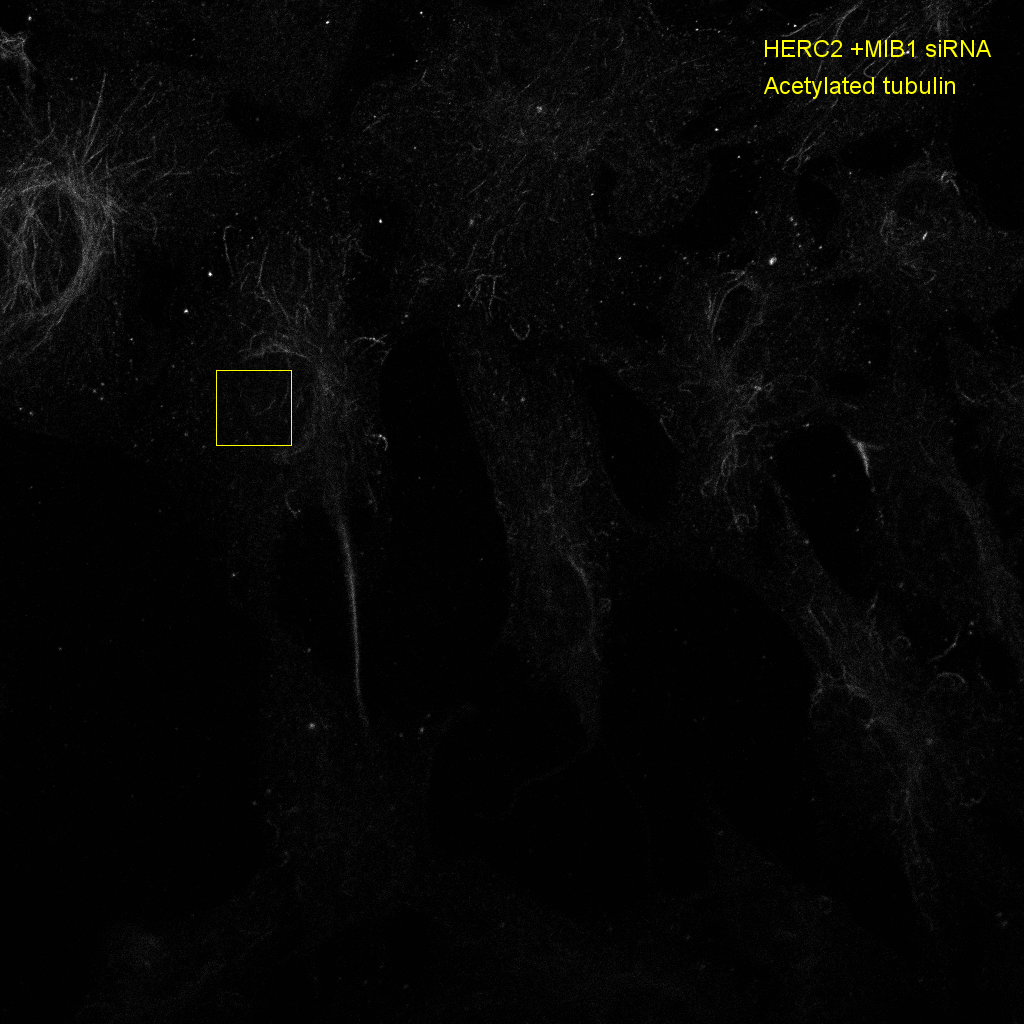

Supplement: Supplementary file 8 — Source Data for Figure 4 [file EMBR-24-e56317-s008.zip › Figure 4/4L/Micr.image HERC2 + MIB1 siRNA -acetylated tubulin.tif]

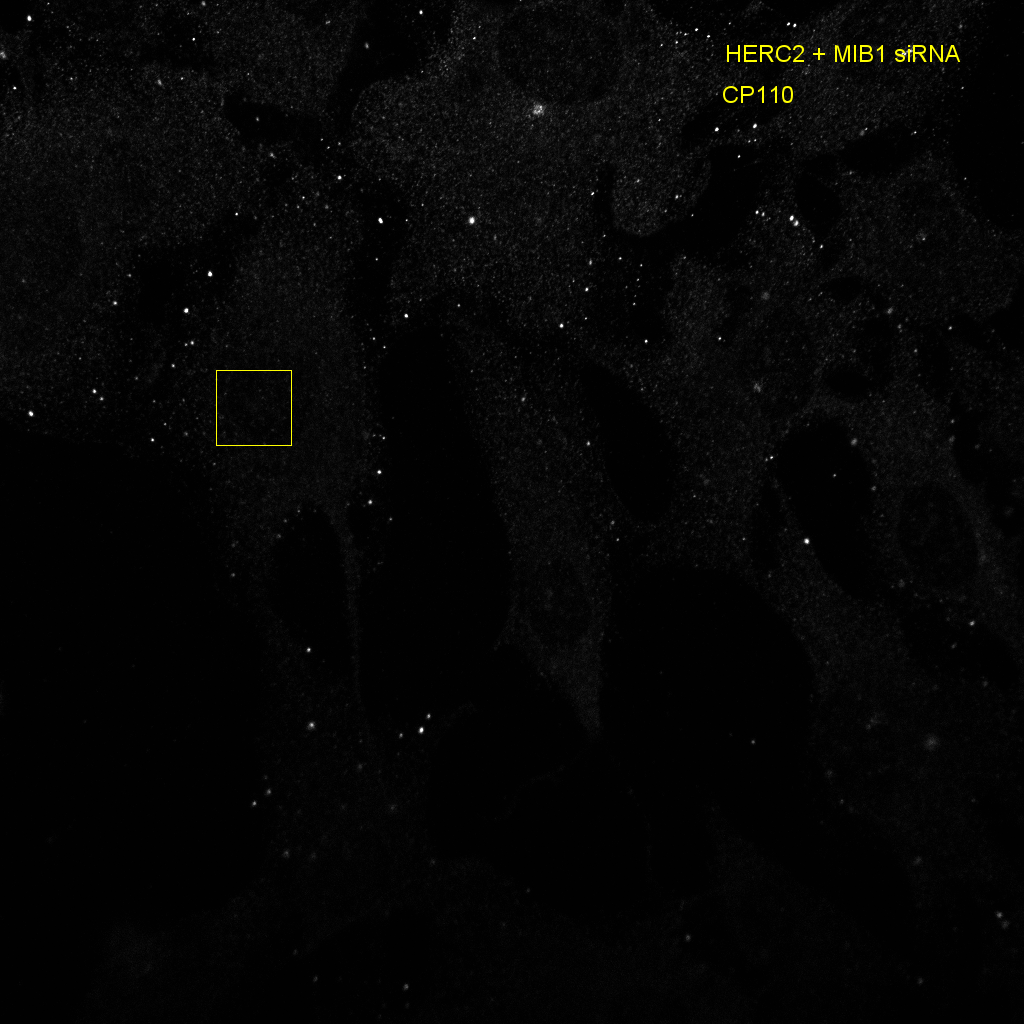

Supplement: Supplementary file 8 — Source Data for Figure 4 [file EMBR-24-e56317-s008.zip › Figure 4/4M/Micr.image HERC2+MIB1 siRNA-CP110.tif]

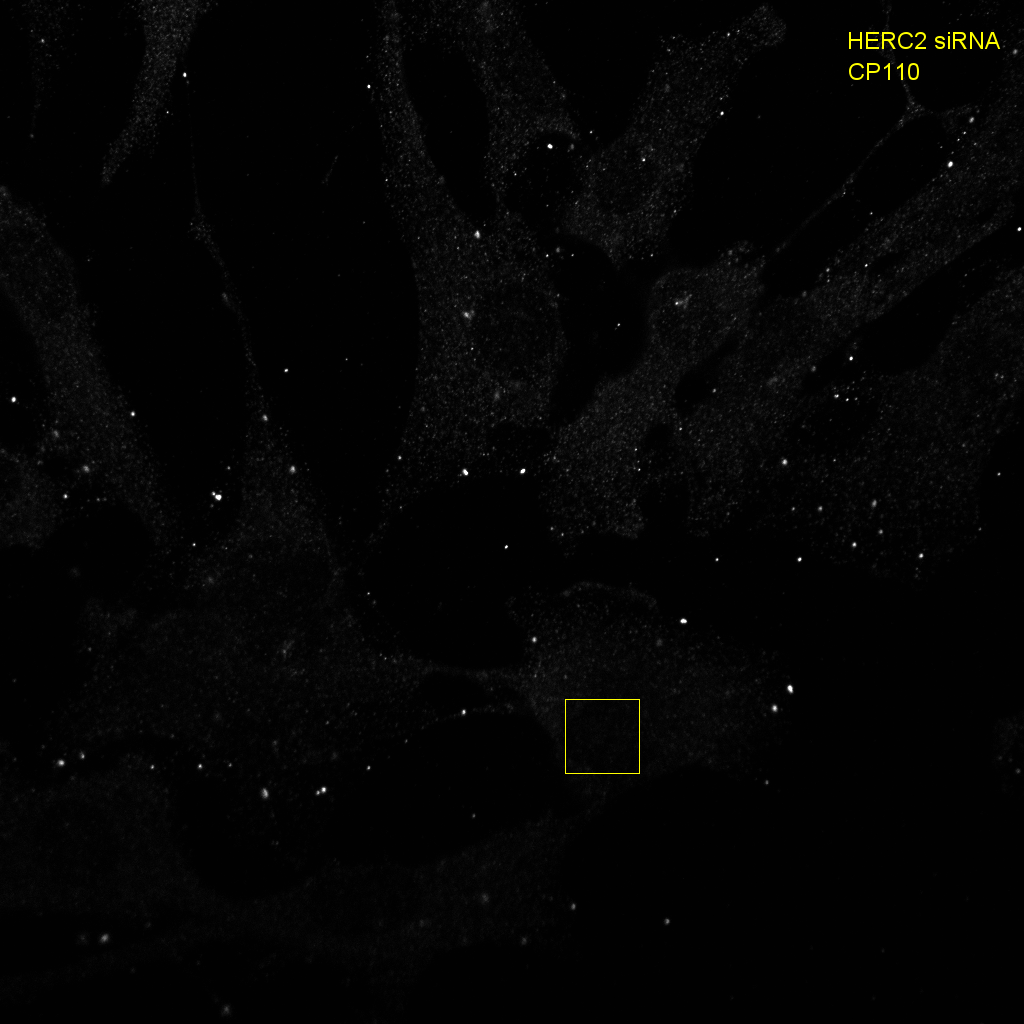

Supplement: Supplementary file 8 — Source Data for Figure 4 [file EMBR-24-e56317-s008.zip › Figure 4/4J/Micr.image HERC2 siRNA-CP110.tif]

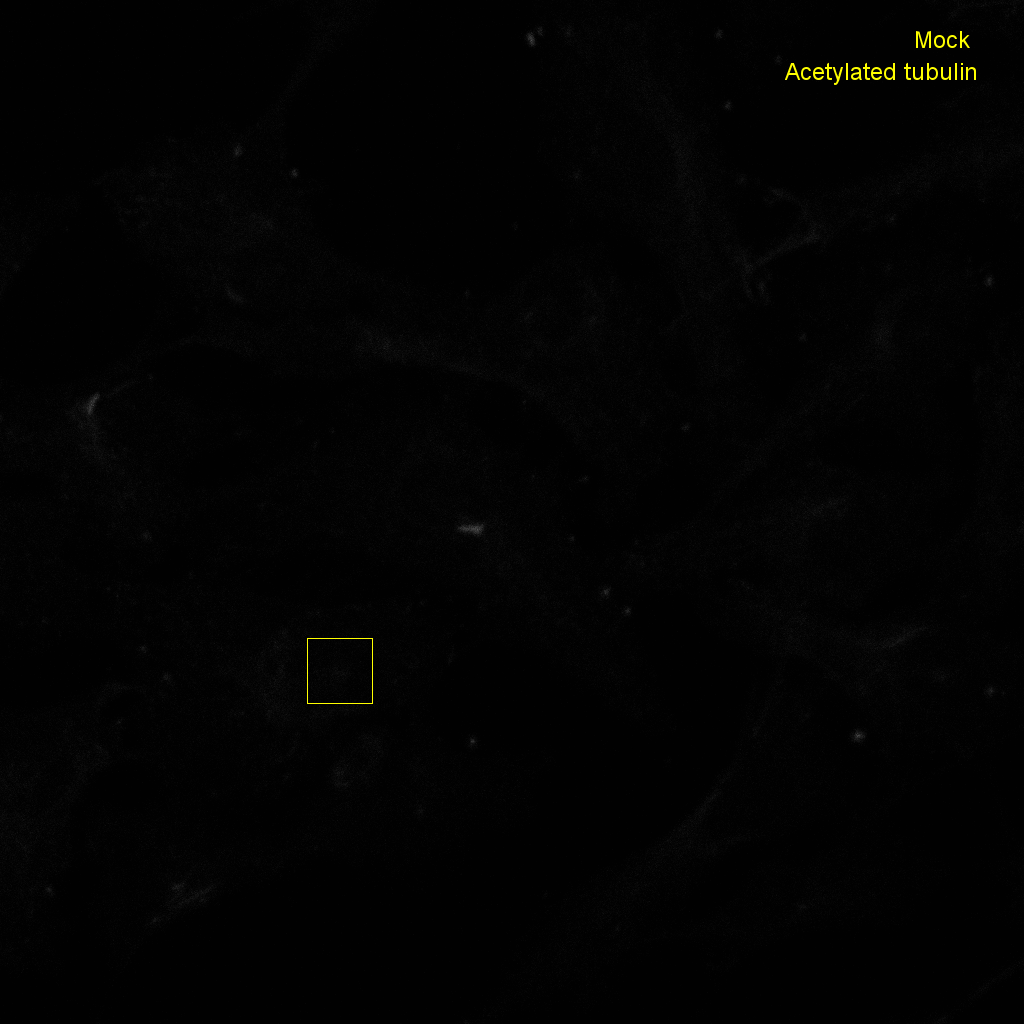

Supplement: Supplementary file 8 — Source Data for Figure 4 [file EMBR-24-e56317-s008.zip › Figure 4/4C/Micr.image Mock-acetylated tubulin.tif]

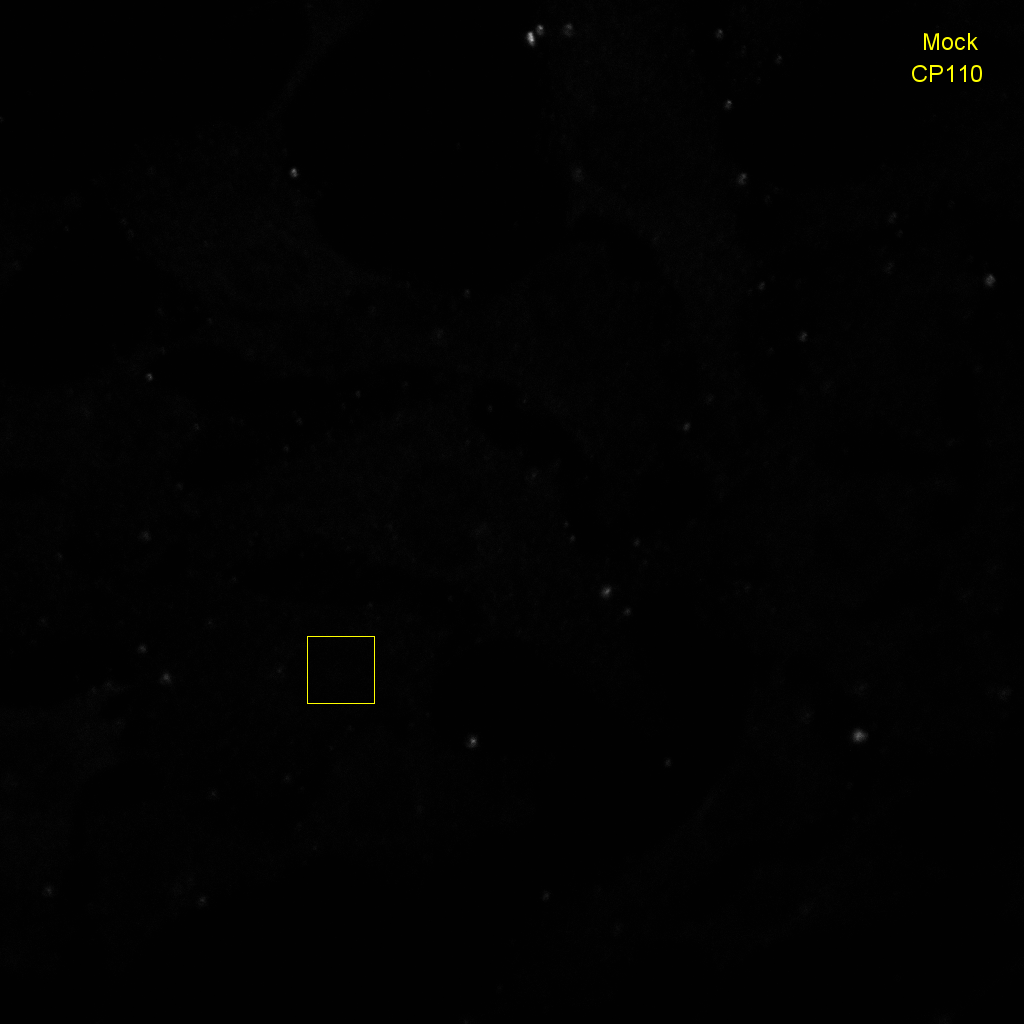

Supplement: Supplementary file 8 — Source Data for Figure 4 [file EMBR-24-e56317-s008.zip › Figure 4/4D/Micr.image Mock-CP110.tif]

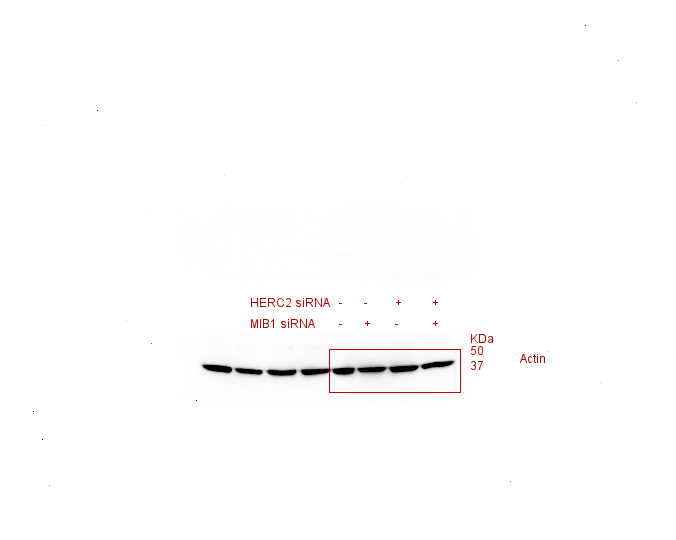

Supplement: Supplementary file 8 — Source Data for Figure 4 [file EMBR-24-e56317-s008.zip › Figure 4/4A/Western actin.tif]

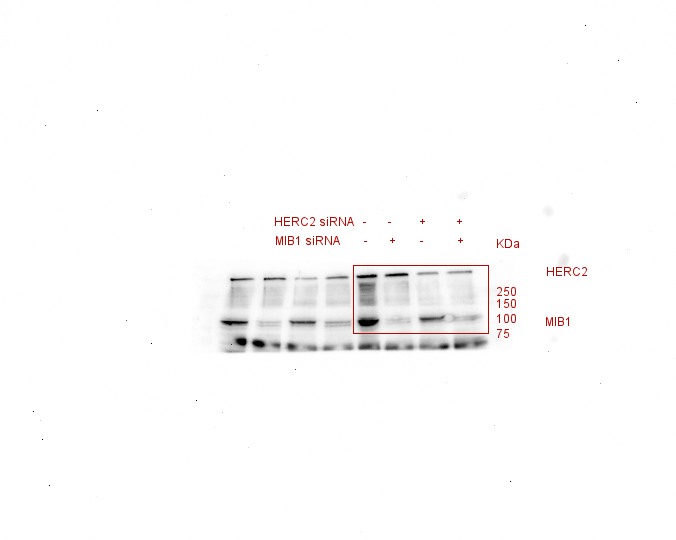

Supplement: Supplementary file 8 — Source Data for Figure 4 [file EMBR-24-e56317-s008.zip › Figure 4/4A/Western HERC2+MIB1.tif]

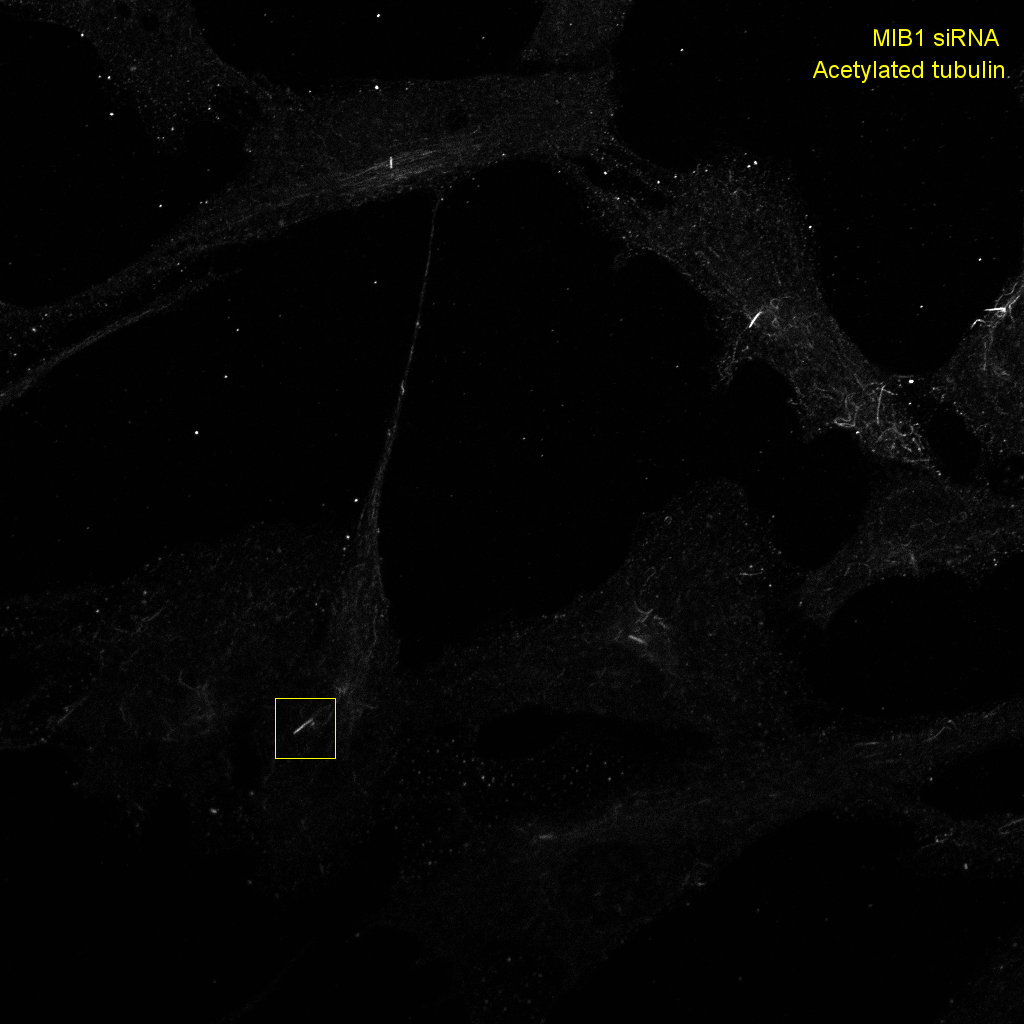

Supplement: Supplementary file 8 — Source Data for Figure 4 [file EMBR-24-e56317-s008.zip › Figure 4/4F/Micr.image MIB1 siRNA-acetylated tubulin.tif]

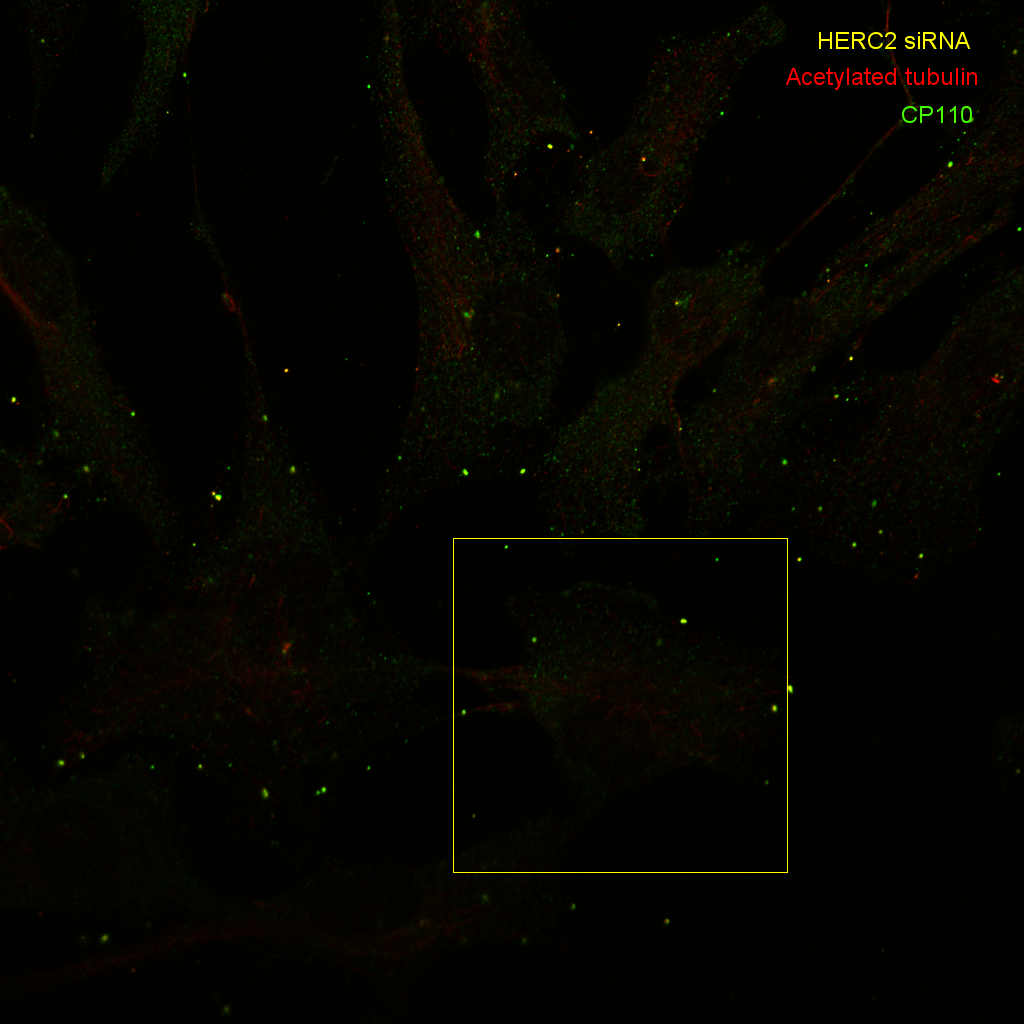

Supplement: Supplementary file 8 — Source Data for Figure 4 [file EMBR-24-e56317-s008.zip › Figure 4/4H/Micr.image HERC2 siRNA.tif]

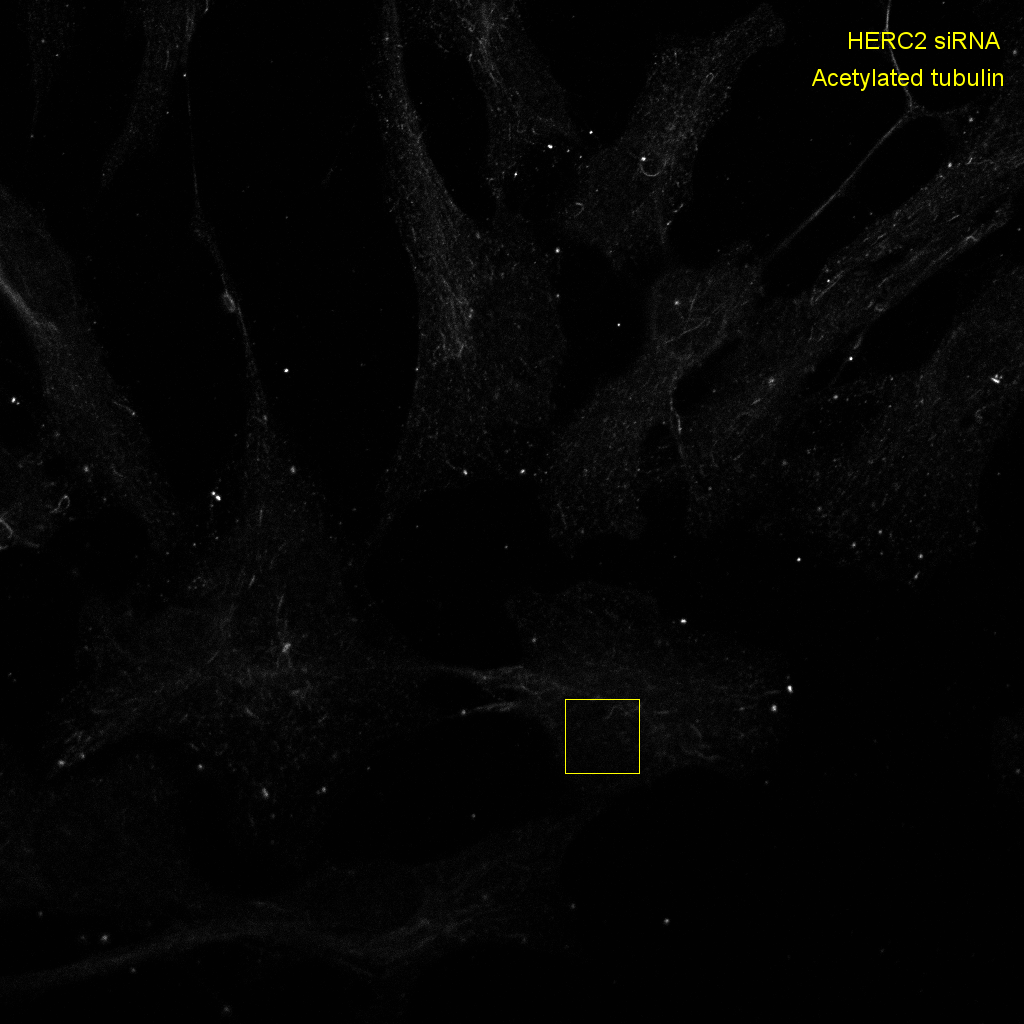

Supplement: Supplementary file 8 — Source Data for Figure 4 [file EMBR-24-e56317-s008.zip › Figure 4/4I/Micr.image HERC2 siRNA-acetylated tubulin.tif]

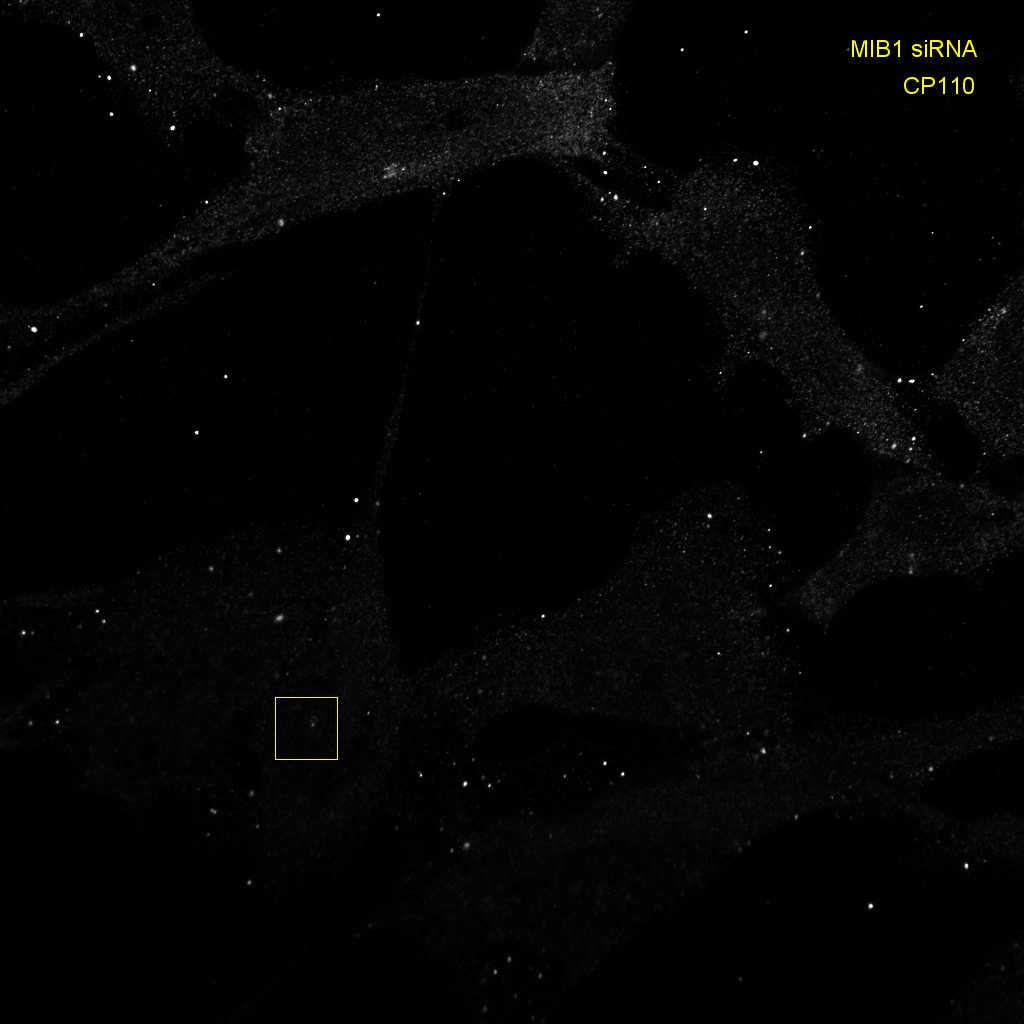

Supplement: Supplementary file 8 — Source Data for Figure 4 [file EMBR-24-e56317-s008.zip › Figure 4/4G/Micr.image MIB1 siRNA-CP110.tif]

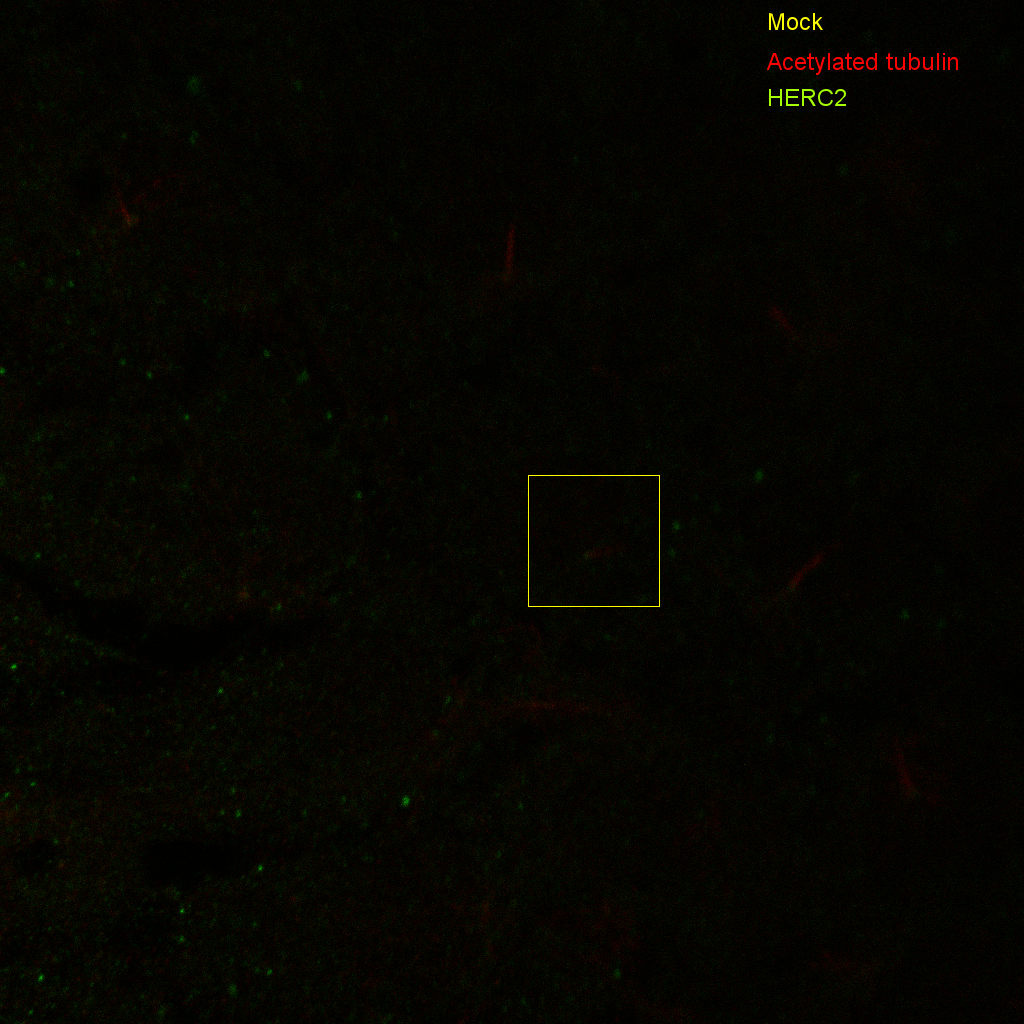

Supplement: Supplementary file 9 — Source Data for Figure 5 [file EMBR-24-e56317-s011.zip › Figure 5/5G/Micr.image Mock.tif]

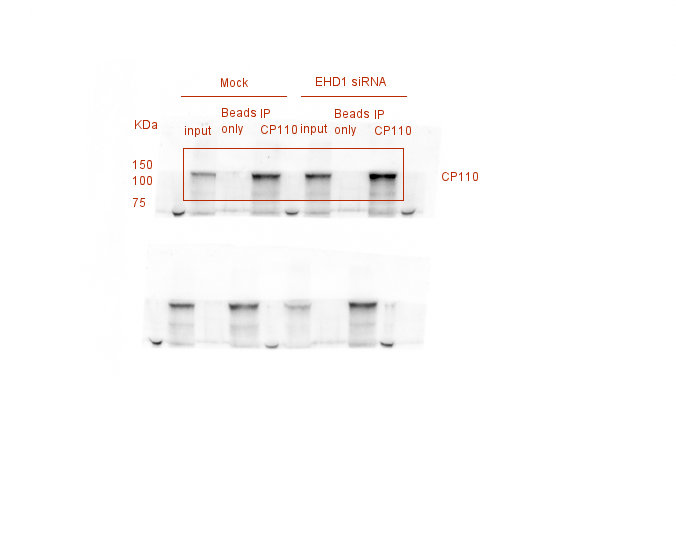

Supplement: Supplementary file 9 — Source Data for Figure 5 [file EMBR-24-e56317-s011.zip › Figure 5/5A/Western CP110.tif]

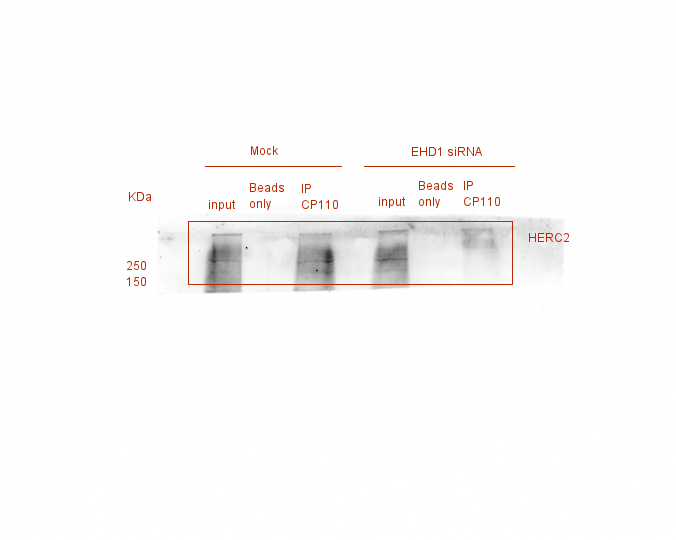

Supplement: Supplementary file 9 — Source Data for Figure 5 [file EMBR-24-e56317-s011.zip › Figure 5/5A/Western Herc2.tif]

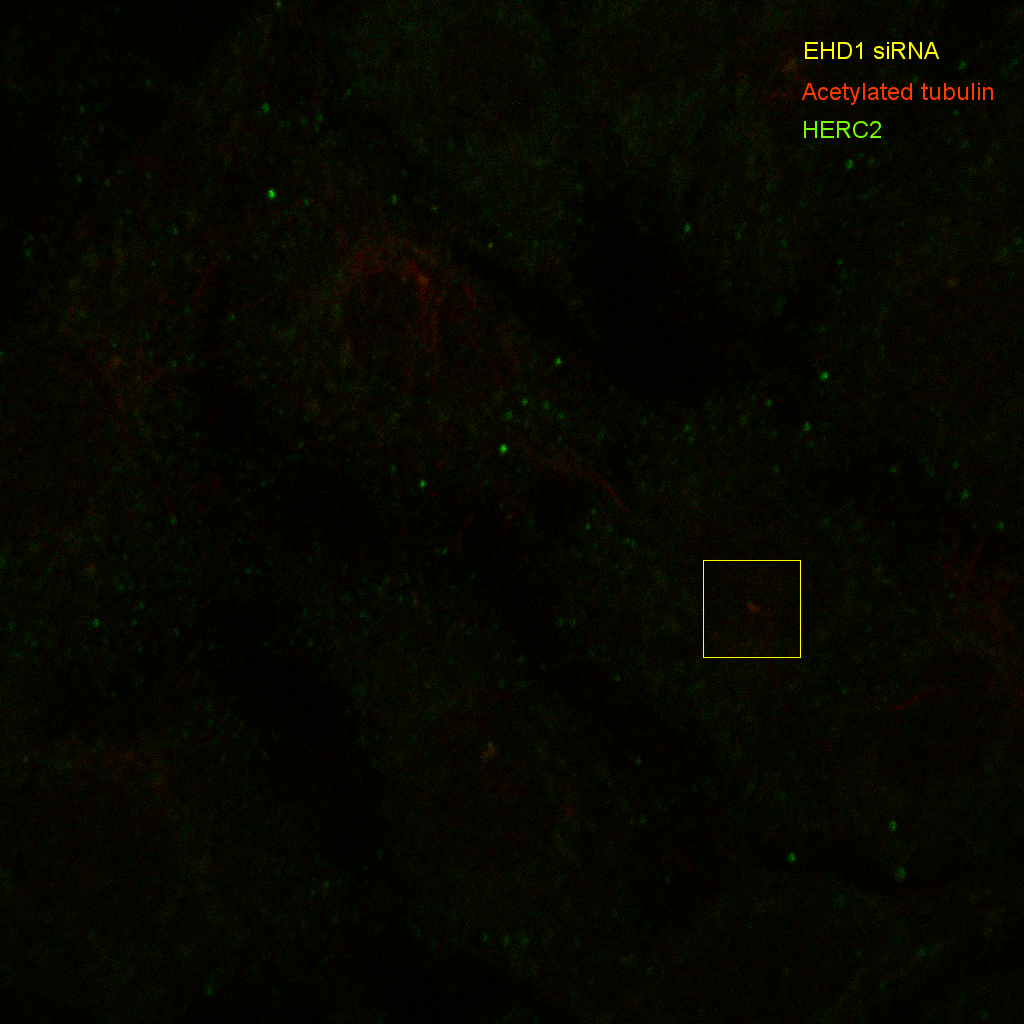

Supplement: Supplementary file 9 — Source Data for Figure 5 [file EMBR-24-e56317-s011.zip › Figure 5/5H/Micr.image EHD1 siRNA.tif]

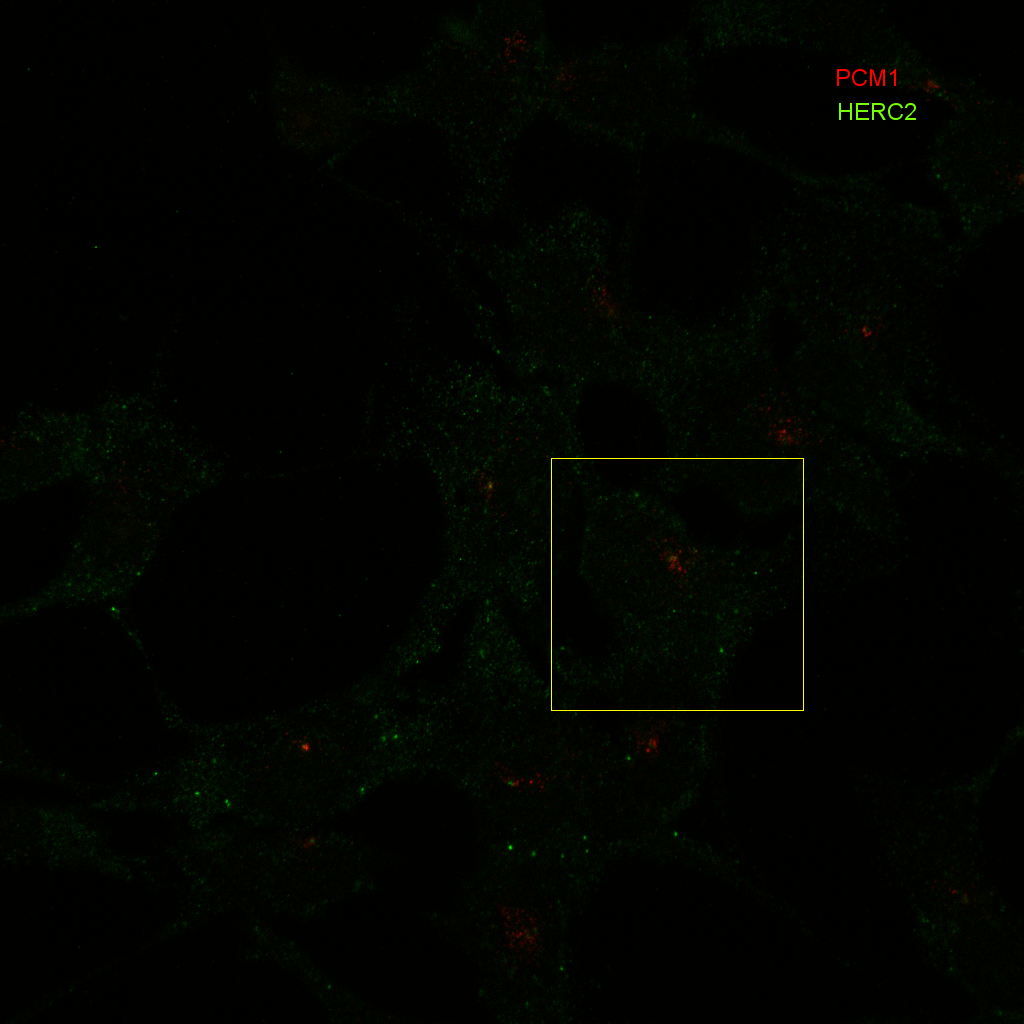

Supplement: Supplementary file 9 — Source Data for Figure 5 [file EMBR-24-e56317-s011.zip › Figure 5/5C/Micr.image PCM1+HERC2.tif]

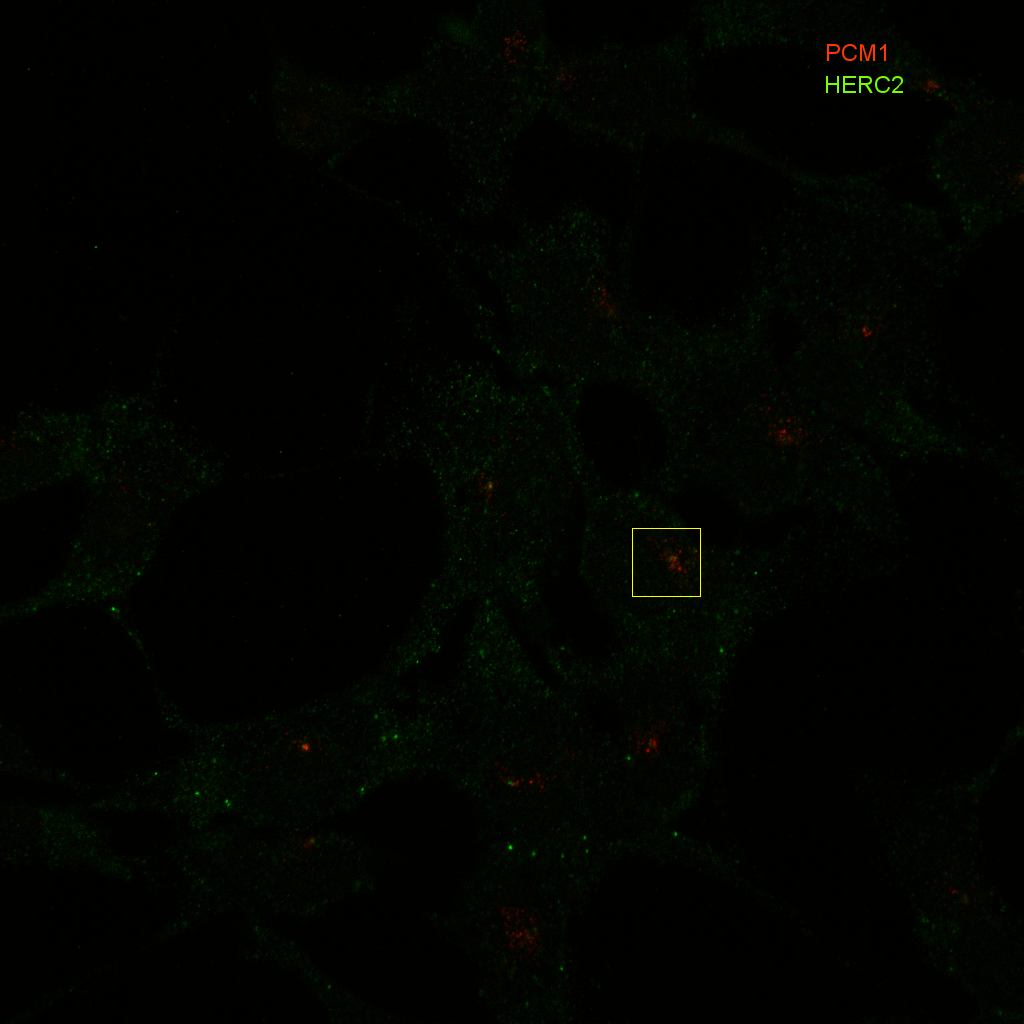

Supplement: Supplementary file 9 — Source Data for Figure 5 [file EMBR-24-e56317-s011.zip › Figure 5/5D/Micr.image PCM1+HERC2 inset.tif]

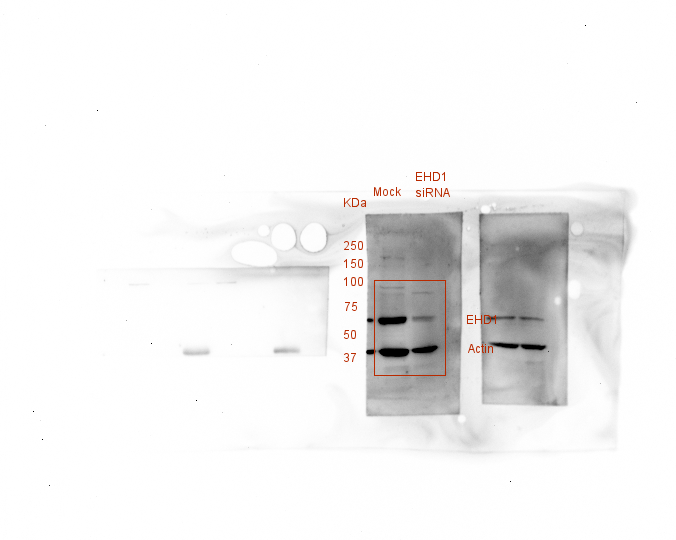

Supplement: Supplementary file 9 — Source Data for Figure 5 [file EMBR-24-e56317-s011.zip › Figure 5/5B/Western EHD1+Actin.tif]

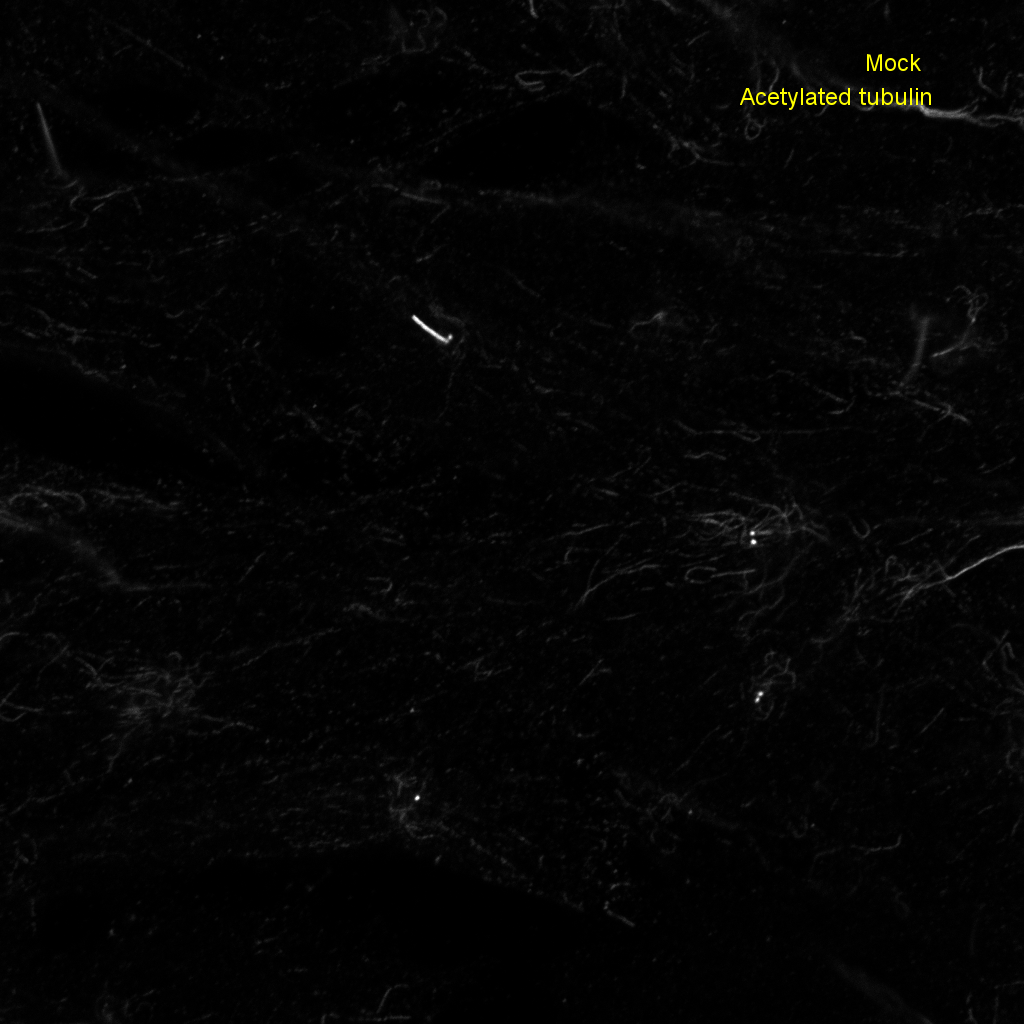

Supplement: Supplementary file 10 — Source Data for Figure 6 [file EMBR-24-e56317-s004.zip › Figure 6/6A/Micr.image Mock.tif]

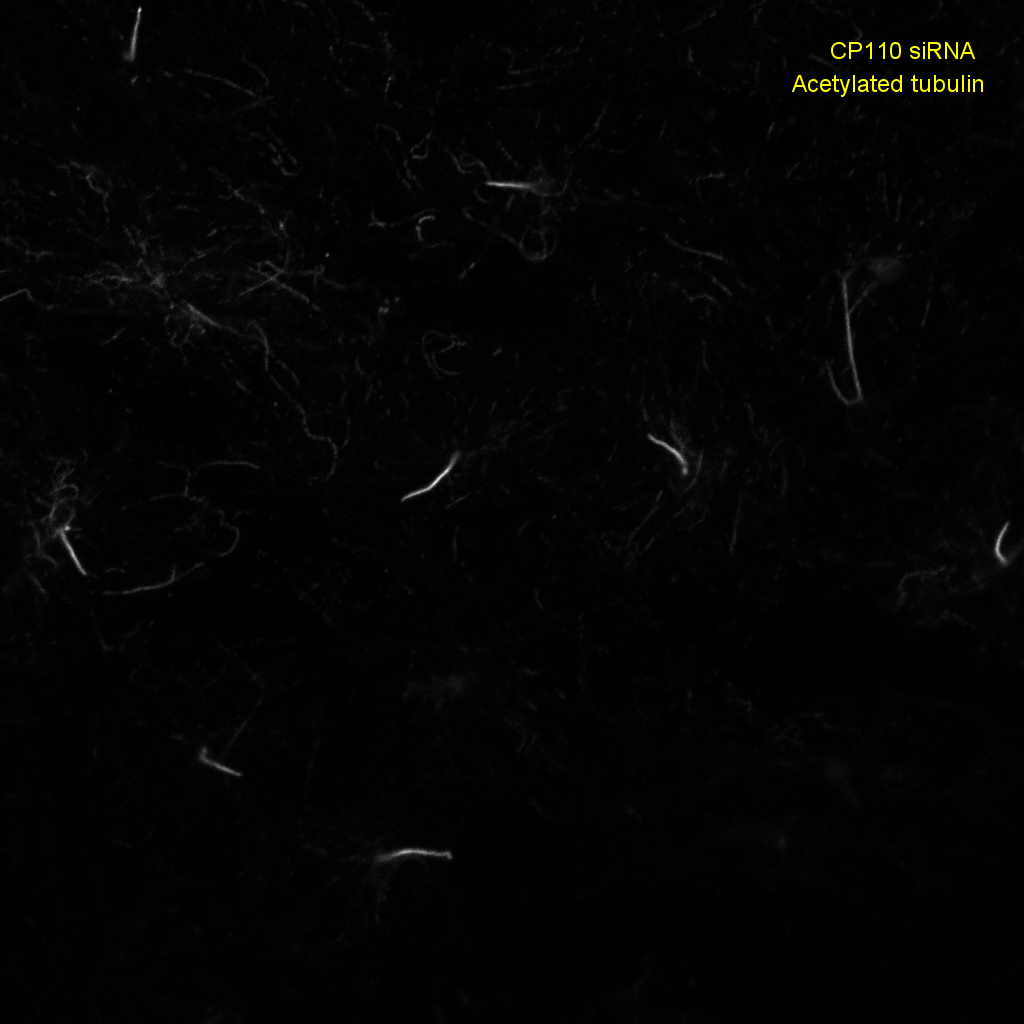

Supplement: Supplementary file 10 — Source Data for Figure 6 [file EMBR-24-e56317-s004.zip › Figure 6/6B/Micr.image CP110 siRNA.tif]

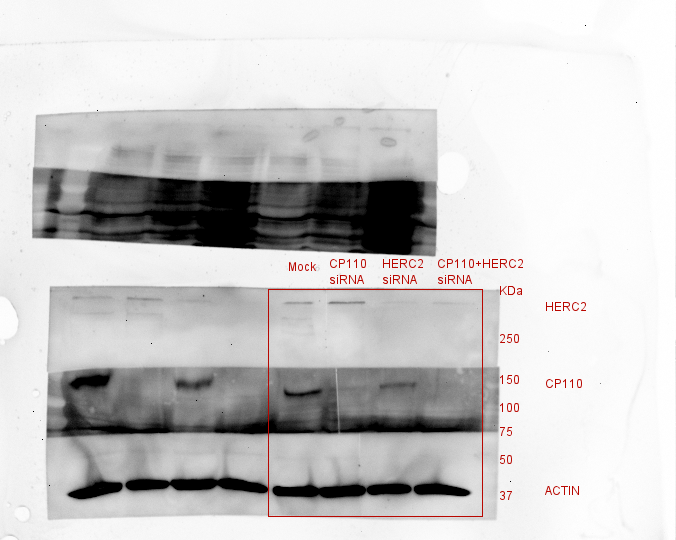

Supplement: Supplementary file 10 — Source Data for Figure 6 [file EMBR-24-e56317-s004.zip › Figure 6/6E/Western HERC2+CP110+Actin.tif]

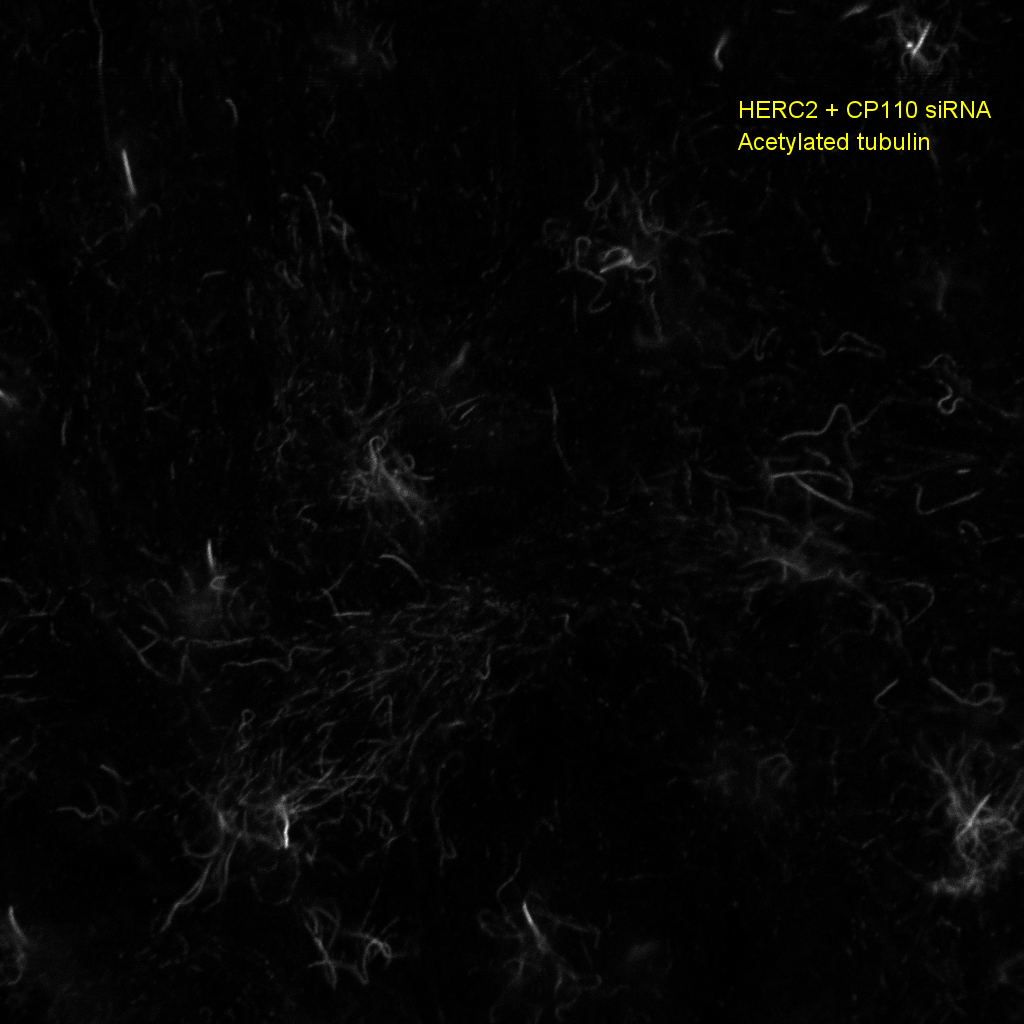

Supplement: Supplementary file 10 — Source Data for Figure 6 [file EMBR-24-e56317-s004.zip › Figure 6/6D/Micr.image HERC2+CP110 siRNA.tif]

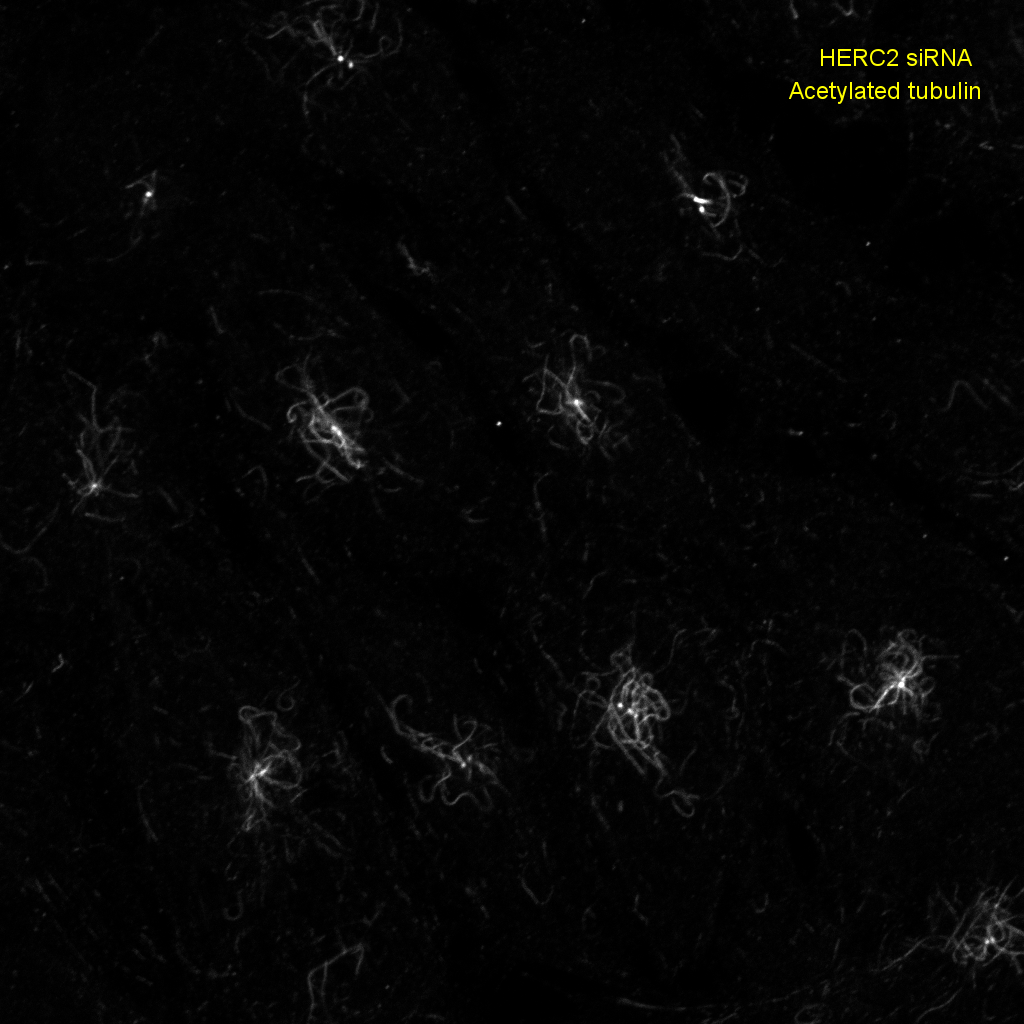

Supplement: Supplementary file 10 — Source Data for Figure 6 [file EMBR-24-e56317-s004.zip › Figure 6/6C/Micr.image HERC2 siRNA.tif]
