# Supplementary material for: EHD1 promotes CP110 ubiquitination by centriolar satellite delivery of HERC2 to the mother centriole
Source: EMBO Rep. 2023 Apr 19;24(6):e56317. doi: 10.15252/embr.202256317 (PMC10240189; doi:10.15252/embr.202256317)
Supplement: Supplementary file 11 [file EMBR-24-e56317-s001.pdf]

EMBO reports

EHD1 promotes CP110 ubiquitination by centriolar satellite delivery of HERC2 to the mother centriole

Shuwei Xie, Naava Naslavsky, and Steve Caplan
DOI: 10.15252/embr.202256317

Corresponding author(s): Steve Caplan (scaplan@unmc.edu)

Review Timeline:

Submission Date:	17th Oct 22
Editorial Decision:	28th Nov 22
Revision Received:	13th Feb 23
Editorial Decision:	29th Mar 23
Revision Received:	30th Mar 23
Accepted:	3rd Apr 23

Editor: Martina Rembold

Transaction Report:

(Note: With the exception of the correction of typographical or spelling errors that could be a source of ambiguity, letters and reports are not edited. Depending on transfer agreements, referee reports obtained elsewhere may or may not be included in this compilation. Referee reports are anonymous unless the Referee chooses to sign their reports.)

1st Editorial Decision 28th Nov 2022

Dear Prof. Caplan

Thank you for the submission of your research manuscript to EMBO reports. I apologize for the delay in handling it. Your manuscript has been sent to three referees, and so far we have received reports from two of them, which I copy below. As both referees feel that the manuscript is interesting and recommend that you should be given a chance to revise it, I would like to ask you to begin revising your manuscript according to the referees' comments. Please note that this is a preliminary decision made in the interest of time, and that it is subject to change should the third referee offer very strong and convincing reasons for this.

In the current reports, referee 1 recommends publication 'as is' but referee 2 raises a number of concerns regarding quantification, the level of CP110 ubiquitylation and MG132's effect on the cell cycle that should be addressed. I will forward you the report from referee 3 as soon as we have it, which should happen within the next few days.

Given these constructive comments, we would like to invite you to revise your manuscript with the understanding that the referee concerns (as detailed above and in their reports) must be fully addressed and their suggestions taken on board. Please address all referee concerns in a complete point-by-point response. Acceptance of the manuscript will depend on a positive outcome of a second round of review. It is EMBO reports policy to allow a single round of revision only and acceptance or rejection of the manuscript will therefore depend on the completeness of your responses included in the next, final version of the manuscript.

We realize that it is difficult to revise to a specific deadline. In the interest of protecting the conceptual advance provided by the work, we recommend a revision within 3 months (February 28th). Please discuss the revision progress ahead of this time with the editor if you require more time to complete the revisions.

I list below our formatting guidelines, but let me point out one thing here: We need the movies as .ZIP file containing the movie and the legend as simple README.txt file.

*****IMPORTANT NOTE:
We perform an initial quality control of all revised manuscripts before re-review. Your manuscript will FAIL this control and the handling will be DELAYED if the following APPLIES:

1) A data availability section providing access to data deposited in public databases is missing. If you have not deposited any data, please add a sentence to the data availability section that explains that.

2) Your manuscript contains statistics and error bars based on n=2. Please use scatter blots in these cases. No statistics should be calculated if n=2.

When submitting your revised manuscript, please carefully review the instructions that follow below. Failure to include requested items will delay the evaluation of your revision.*****

When submitting your revised manuscript, we will require:

1) a .docx formatted version of the manuscript text (including legends for main figures, EV figures and tables). Please make sure that the changes are highlighted to be clearly visible.

2) individual production quality figure files as .eps, .tif, .jpg (one file per figure).
Please download our Figure Preparation Guidelines (figure preparation pdf) from our Author Guidelines pages https://www.embopress.org/page/journal/14693178/authorguide for more info on how to prepare your figures.

3) a .docx formatted letter INCLUDING the reviewers' reports and your detailed point-by-point responses to their comments. As part of the EMBO Press transparent editorial process, the point-by-point response is part of the Review Process File (RPF), which will be published alongside your paper.

4) a complete author checklist, which you can download from our author guidelines (). Please insert information in the checklist that is also reflected in the manuscript. The completed author checklist will also be part of the RPF.

5) Please note that all corresponding authors are required to supply an ORCID ID for their name upon submission of a revised manuscript (). Please find instructions on how to link your ORCID ID to your account in our manuscript tracking system in our Author guidelines
()

6) We replaced Supplementary Information with Expanded View (EV) Figures and Tables that are collapsible/expandable online. A maximum of 5 EV Figures can be typeset. EV Figures should be cited as 'Figure EV1, Figure EV2" etc... in the text and their respective legends should be included in the main text after the legends of regular figures.

- For the figures that you do NOT wish to display as Expanded View figures, they should be bundled together with their legends in a single PDF file called *Appendix*, which should start with a short Table of Content. Appendix figures should be referred to in the main text as: "Appendix Figure S1, Appendix Figure S2" etc. See detailed instructions regarding expanded view here:

- Additional Tables/Datasets should be labeled and referred to as Table EV1, Dataset EV1, etc. Legends have to be provided in a separate tab in case of .xls files. Alternatively, the legend can be supplied as a separate text file (README) and zipped together with the Table/Dataset file.

7) Please note that a Data Availability section at the end of Materials and Methods is now mandatory. In case you have no data that requires deposition in a public database, please state so instead of refereeing to the database.
See also < https://www.embopress.org/page/journal/14693178/authorguide#dataavailability>). Please note that the Data Availability Section is restricted to new primary data that are part of this study.

8) At EMBO Press we ask authors to provide source data for the main figures. Our source data coordinator will contact you to discuss which figure panels we would need source data for and will also provide you with helpful tips on how to upload and organize the files.

Additional information on source data and instruction on how to label the files are available .

9) The journal requires a statement specifying whether or not authors have competing interests (defined as all potential or actual interests that could be perceived to influence the presentation or interpretation of an article). In case of competing interests, this must be specified in your disclosure statement. Further information: https://www.embopress.org/competing-interests

10) Figure legends and data quantification:
The following points must be specified in each figure legend:

- the name of the statistical test used to generate error bars and P values,
- the number (n) of independent experiments (please specify technical or biological replicates) underlying each data point,
- the nature of the bars and error bars (s.d., s.e.m.)

- If the data are obtained from n {less than or equal to} 5, show the individual data points in addition to the SD or SEM.
- If the data are obtained from n {less than or equal to} 2, use scatter blots showing the individual data points.

Discussion of statistical methodology can be reported in the materials and methods section, but figure legends should contain a basic description of n, P and the test applied.

See also the guidelines for figure legend preparation:
https://www.embopress.org/page/journal/14693178/authorguide#figureformat

- Please also include scale bars in all microscopy images.

11) Our journal encourages inclusion of *data citations in the reference list* to directly cite datasets that were re-used and obtained from public databases. Data citations in the article text are distinct from normal bibliographical citations and should directly link to the database records from which the data can be accessed. In the main text, data citations are formatted as follows: "Data ref: Smith et al, 2001" or "Data ref: NCBI Sequence Read Archive PRJNA342805, 2017". In the Reference list, data citations must be labeled with "[DATASET]". A data reference must provide the database name, accession number/identifiers and a resolvable link to the landing page from which the data can be accessed at the end of the reference. Further instructions are available at .

12) As part of the EMBO publication's Transparent Editorial Process, EMBO reports publishes online a Review Process File to accompany accepted manuscripts. This File will be published in conjunction with your paper and will include the referee reports, your point-by-point response and all pertinent correspondence relating to the manuscript.

You are able to opt out of this by letting the editorial office know (emboreports@embo.org). If you do opt out, the Review Process File link will point to the following statement: "No Review Process File is available with this article, as the authors have chosen not to make the review process public in this case."

We would also welcome the submission of cover suggestions, or motifs to be used by our Graphics Illustrator in designing a cover.

I look forward to seeing a revised form of your manuscript when it is ready. Please use this link to submit your revision:
https://embor.msubmit.net/cgi-bin/main.plex

Yours sincerely,

Martina Rembold, PhD
Senior Editor
EMBO reports

****

Referee #1:

This is a very interesting story presented a series of well-executed experiments showing that EDH1 plays a role in delivering the HERC2 E3 enzyme on pericentriolar satellites to ubiquitinate CP110 to enable its removal from centrioles to permit ciliogenesis. The authors first convincingly demonstrate a role for the endocytic regulatory protein EDH1 in regulating CP110 ubiquitination and degradation, thus permitting ciliogenesis. They show this process requires pericentriolar satellite function by depleting PCM1 and showing that CP110 remains at the centriole and cilia rarely form. Moreover, EDH1 is required for the integrity of the pericentriolar satellites. Of the E3 ligases associated with pericentriolar satellites, they show that CP110-associated HERC2 is required for ciliogenesis. EDH1 depletion prevents the interaction between HERC2 and CP110 leading to the conclusion that EDH1 regulates access of HERC2 to the centriolar region to ubiquitinate CP110.
Together this is a convincing story that is clearly described and is suitable for publication without requiring any revision.

Referee #2:

The initiation of ciliogenesis is a tightly regulated process that requires the uncapping of the mother centriole by CP110 to promote axonemal growth. Many proteins have been proposed to contribute to the removal of CP110 from the mother centriole such as the ubiquitin ligases HERC2 and SCFcyclinF. It has also been shown that uncapping the mother centriole requires EHD1-dependent ciliary vesicle formation. Here, the authors show that EHD1 and PCM1, as well as the ubiquitin ligases HERC2 and MIB1, are involved in the ubiquitination of CP110 in RPE1 cells. Because MIB1 deficiency does not affect the removal of CP110 from the mother centriole, unlike the removal of HCER2 (and also EHD1 and PCM1), these results indicate that HCER2 plays a prominent role in the ubiquitination of CP110 and removal from the mother centriole. In agreement with this result, epistatic analysis shows that HERC2 function can be rescued by CP110 removal.

The authors also show that HERC2 is associated with centriolar satellites as revealed by its colocalization with PCM1, in agreement with another published study showing the association of PCM1 and HERC2 (Quarantotti et al., 2019). More unexpectedly the authors show that the targeting of HERC2 and PCM1 to centrioles is dependent on EHD1. This is a novel conceptual result of this study because EHD1 has been associated with endocytic vesicular transport but not yet with centriolar satellites. This suggests an EHD1-dependent mechanism of centriolar satellite transport still unexplained by this study and for which the authors only propose several hypotheses in their discussion. Together, this study provides another level of complexity for CP110 removal from the mother centriole by uncovering a novel regulation of centriolar satellite mediated transport of HERC2 towards the centriole that has yet to be understood.

I have a few concerns about Figure 1 and a few figure panels that need to be addressed for publication as listed below:

Figure 1A: quantifications of the WB should be performed to demonstrate the reduced amount of CP110 after serum deprivation.

Figure 1B: in the text we understand that the input fraction is before IP by anti-ubiquitin antibodies. However, the figure legend says "the membrane was reblotted with CP110 antibody (right panel, input). This is confusing. If right panel is reblotting of left panel, then this means that only an extremely small fraction of CP110 is ubiquitinylated as there is only one band detected for CP110 using CP110 antibody and not even a faint smear like for the right panel (Ub-CP110 should be recognized by CP110 antibody)?
Is this difference sufficient to explain CP110 reduction after 4h of serum starvation, as observed on Figure 1A ?

Figure 1C: in the presence of MG132 apparently 4 centrioles are present suggesting cells are in G2. Does this mean there is any difference in cell cycle using MG132 which could distort counting of ciliated cells and CP110 on centrioles?

Minor

Figure 1B: please provide quantifications

Figure 1G and 1I: quantifications are provided but the normalization to loading control (actin? or CP110 in I) even if obvious should be mentioned.

Figure 2AB: please explain how normalization was performed for the quantification

Figure 3B:please provide quantifications of the WB

Dear Prof. Caplan

We have meanwhile received the report from the third referee, which I herewith forward to you. As you will see, also referee 3 submitted a positive evaluation but suggests several control experiments, asks for clarification of certain points and the repeat of some key experiments to support conclusions. Please also address these concerns in the revised version.

In addition, referee 3 suggests to establish a hierarchy between the different E3 ligases for CP110, which would certainly strengthen the paper but these experiments are not mandatory. I suggest however to at least test the interplay between HERC2 and LUBAC.

Kind regards,

Martina Rembold, PhD
Senior Editor
EMBO reports

****

Referee #3

Removal of CP110 from the distal tip of mother centrioles is a key step in early ciliogenesis. Caplan and colleagues previously identified EHD1 as an important regulator of CP110 removal. In this manuscript they propose that EHD1 modulates CP110 degradation on the distal end of the mother centriole via centriolar satellite transport of the E3 ligase HERC2. In particular, they find that EHD1 depletion reduces levels of the satellite protein PCM1 near centrosomes and demonstrate that CP110 and HERC2 function in same molecular pathway.

The manuscript is well-written and concise, and data quality is generally good. In my view the key discovery here is the HERC2-dependency of CP110 removal from centrioles. Given that besides HERC2, the ubiquitin ligases LUBAC, EDD-DYRK2-DDB1 and SCF/cyclinF have all been shown to degrade/remove CP110 from centrioles, I wonder if the authors could try to establish a hierarchy between these enzymes. They do discuss a possible interplay between HERC2 and LUBAC but testing the idea they put forward would be beneficial for the paper as well as the field.

Major points:

1. The impact of EHD1 on centriolar satellite distribution and PCM1 intensity is interesting but is somewhat preliminary and based largely on correlations. It is unclear how EHD1 controls motility of centriolar satellites and whether the observed change in centriolar satellite distribution is the cause or consequence of EHD1 depletion. PCM1 intensity should be measured separately in ciliated and non-ciliated cells in mock- and EHD1-depleted cells. I realise that in EHD1-depleted cells there will be much fewer cilia, and the ones with cilia are likely to represent cells

with residual EHD1 activity, but these could still serve as positive controls.

2. Co-depletion of CP110 with HERC2 clearly shows rescue of ciliogenesis. However, from the image cilia of co-depleted cells appear shorter and perhaps bendier. Is that so? Also, there is a notable change in overall acetylation levels of tubulin in cells. A closer examination of these cells could provide additional insight.

3. Fig 1H, 2A: IP efficiency should also be demonstrated with CP110 antibody. Densitometry analysis could then be normalised against total immunoprecipitated CP110.

4. Fig 3A: HERC2 blot is very poor, specificity is unclear. Cullin 3 is weakly depleted.

5 Fig 3B: Again, a blot to show efficacy of CP110 immunoprecipitation is lacking. Quantitative analysis by densitometry is needed.

6. Fig 5A: the blot demonstrating lack of interaction between CP110 and HERC2 in absence of EHD1 is really important. The current blot does not provide high enough quality proof due to weak HERC2 signal and the unfortunate shading on top of blot in the EHD1 siRNA lanes.

7. Fig 4F-M: images are very pixelated

8. Figures generally include statistical analysis, although the actual test carried out should be mentioned in figure legends. For measuring intensities in images (i.e. PCM1, HERC2) or mean particle speeds, it would be more informative to provide dot/box plots of actual datapoints and compare distributions rather than a single average value per experiment.

Minor points:

Lack of line and page numbers made it difficult to reference text.

1. I am surprised that the greatest increase in HERC2 levels at centrosomes occurs within 10 minutes of serum withdrawal. How would the authors explain this very fast response?

2. According to 1D, over 70% of cells grow a primary cilia within 4 hours of serum starvation. This again seems higher than values reported in the literatures. By the same measure, in Fig 6E mock transfected cells show only 50% ciliation.

3. "they might assemble in an immune complex" Shouldn't this be 'protein complex'?

4. HERC2 has been shown to positively regulate ciliogenesis (Quarantotti et al, EMBO J, 2019).

Point-by-Point Response to Reviewers' Critiques:

Referee #1:

This is a very interesting story presented a series of well-executed experiments showing that EDH1 plays a role in delivering the HERC2 E3 enzyme on pericentriolar satellites to ubiquitinate CP110 to enable its removal from centrioles to permit ciliogenesis.
The authors first convincingly demonstrate a role for the endocytic regulatory protein EDH1 in regulating CP110 ubiquitination and degradation, thus permitting ciliogenesis. They show this process requires pericentriolar satellite function by depleting PCM1 and showing that CP110 remains at the centriole and cilia rarely form. Moreover, EDH1 is required for the integrity of the pericentriolar satellites. Of the E3 ligases associated with pericentriolar satellites, they show that CP110-associated HERC2 is required for ciliogenesis. EDH1 depletion prevents the interaction between HERC2 and CP110 leading to the conclusion that EDH1 regulates access of HERC2 to the centriolar region to ubiquitinate CP110. Together this is a convincing story that is clearly described and is suitable for publication without requiring any revision.

We thank the reviewer and appreciate the support for acceptance/publication of our manuscript in its current form.

Referee #2:

The initiation of ciliogenesis is a tightly regulated process that requires the uncapping of the mother centriole by CP110 to promote axonemal growth. Many proteins have been proposed to contribute to the removal of CP110 from the mother centriole such as the ubiquitin ligases HERC2 and SCFcyclinF. It has also been shown that uncapping the mother centriole requires EHD1-dependent ciliary vesicle formation. Here, the authors show that EHD1 and PCM1, as well as the ubiquitin ligases HERC2 and MIB1, are involved in the ubiquitination of CP110 in RPE1 cells. Because MIB1 deficiency does not affect the removal of CP110 from the mother centriole, unlike the removal of HCER2 (and also EHD1 and PCM1), these results indicate that HCER2 plays a prominent role in the ubiquitination of CP110 and removal from the mother centriole. In agreement with this result, epistatic analysis shows that HERC2 function can be rescued by CP110 removal.

The authors also show that HERC2 is associated with centriolar satellites as revealed by its colocalization with PCM1, in agreement with another published study showing the association of PCM1 and HERC2 (Quarantotti et al., 2019). More unexpectedly the authors show that the targeting of HERC2 and PCM1 to centrioles is dependent on EHD1. This is a novel conceptual result of this study because EHD1 has been associated with endocytic vesicular transport but not yet with centriolar satellites. This suggests an EHD1-dependent mechanism of centriolar satellite transport still unexplained by this study and for which the authors only propose several hypotheses in their discussion. Together, this study provides another level of complexity for CP110 removal from the mother centriole by uncovering a novel regulation of centriolar satellite mediated transport of HERC2 towards the centriole that has yet to be understood.

We thank the reviewer for incisive comments and support for our manuscript.

I have a few concerns about Figure 1 and a few figure panels that need to be addressed for publication as listed below:

Figure 1A: quantifications of the WB should be performed to demonstrate the reduced amount of CP110 after serum deprivation.

We have now performed quantification of the decreased CP110 following serum deprivation (see Revised Fig. 1, specifically Fig. 1B).

Figure 1B: in the text we understand that the input fraction is before IP by anti-ubiquitin antibodies. However, the figure legend says "the membrane was reblotted with CP110 antibody (right panel, input). This is confusing. If right panel is reblotting of left panel, then this means that only an extremely small fraction of CP110 is ubiquitinylated as there is only one band detected for CP110 using CP110 antibody and not even a faint smear like for the right panel (Ub-CP110 should be recognized by CP110 antibody)? Is this difference sufficient to explain CP110 reduction after 4h of serum starvation, as observed on Figure 1A ?

We thank the reviewer for this excellent question. First, we have better clarified how we have done this experiment (see Revised Fig. Legend 1, p. 19). Briefly, we used anti-CP110 antibodies to pull down CP110 and then first immunoblotted with anti-ubiquitin to detect ubiquitinated CP110. We then stripped these blots and reblotted with anti-CP110 to detect total CP110 levels. The most likely explanation for detecting only a single CP110 band is that when CP110 undergoes polyubiquitination, the anti-ubiquitin antibody is more likely to recognize even smaller amounts of CP110, because of the multiple ubiquitins present. Thus, even amounts of CP110 that might be poorly detectable by CP110 antibodies might be resolvable by anti-ubiquitin antibodies. Our data is largely consistent with gels for CP110 immunoprecipitated by other published studies (with a single CP110 band seen with anti-CP110 antibodies), including that of the LUBAC study by Shen et. al (2022). With regard to whether this is sufficient to explain the degradation observed in Fig. 1A, it is necessary for us to note several points: 1) It is difficult to compare ubiquitination to CP110 levels because the antibodies used for CP110 and ubiquitin have different affinities, and 2) in Fig. 1A (for the 4 h starvation) the CP110 is likely undergoing continual ubiquitination and degradation over time, whereas with the MG132 treatment in Fig. 1B, there is no continuous (or almost any) proteasomal degradation. In other words, it is possible that at a given time point within the 4 h of starvation, only a relatively small amount of protein is ubiquitinated, and as it undergoes degradation, additional CP110 is ubiquitinated. On the other hand, this will not occur in the 4 h MG132 treatment, because there is continual accumulation of ubiquitinated protein.

Figure 1C: in the presence of MG132 apparently 4 centrioles are present suggesting cells are in G2. Does this mean there is any difference in cell cycle using MG132 which could distort counting of ciliated cells and CP110 on centrioles?

We thank the reviewer for this excellent point; we have now addressed this with a new experiment in shown in new Fig. EV1 (see sample cell cycle experiment and graphs depicting means from 3 experiments). Our data show that upon treatment with MG132, about 20-25% more cells leave G0G1 and enter S phase. On the other hand, we see a 5-fold (500%) reduction in the number of ciliated cells upon MG132 treatment. Thus, at most, only a very small fraction of cells (~5%) could be characterized as failing to generate a primary cilium as a result of potential impact on cell cycle by MG132 treatment. Accordingly, based on these new data and the additional data presented in our manuscript, the primary impact of impaired ciliogenesis described results from failure to ubiquitinate and degrade CP110. We have clarified these points (and added references) within the text on p. 4, lines115-123.

Minor

Figure 1B: please provide quantifications

Former Fig. 1B has now been quantified. It now appears as Fig. 1C, with quantification shown in the graph as Fig. 1D.

Figure 1G and 1I: quantifications are provided but the normalization to loading control (actin? or CP110 in I) even if obvious should be mentioned.

We have now discussed in the legend how normalization was done for the quantification of these experiments.

Figure 2AB: please explain how normalization was performed for the quantification

We have now discussed in the legend how normalization was done for the quantification of these experiment and overall in the study.

Figure 3B:please provide quantifications of the WB

Fig. 3B has now been quantified and normalized, and this is shown now as Fig. 3C.

<u>Referee #3</u>:

Removal of CP110 from the distal tip of mother centrioles is a key step in early ciliogenesis. Caplan and colleagues previously identified EHD1 as an important regulator of CP110 removal. In this manuscript they propose that EHD1 modulates CP110 degradation on the distal end of the mother centriole via centriolar satellite transport of the E3 ligase HERC2. In particular, they find that EHD1 depletion reduces levels of the satellite protein PCM1 near centrosomes and demonstrate that CP110 and HERC2 function in same molecular pathway.

The manuscript is well-written and concise, and data quality is generally good. In my view the key discovery here is the HERC2-dependency of CP110 removal from centrioles. Given that besides HERC2, the ubiquitin ligases LUBAC, EDD-DYRK2-DDB1 and SCF/cyclinF have all been shown to degrade/remove CP110 from centrioles, I wonder if the authors could try to establish a hierarchy between these enzymes. They do discuss a possible interplay between HERC2 and LUBAC but testing the idea they put forward would be beneficial for the paper as well as the field.

We thank the reviewer for the positive feedback and excellent suggestions. The complex and intricate relationship between the established E3 ligases involved in CP110 removal/degradation is an exciting new area that we hope to develop over the next several years. Although a more comprehensive development of the interplay between E3 ligases is both extremely complex and well beyond the scope of our current findings, we have nonetheless carried out several experiments in an attempt to determine the relationship between HERC2 and LUBAC. For example, we have demonstrated and now show in new Fig. EV3 that efficient knockdown of the LUBAC component HOIP does not impede the interaction between CP110 and HERC2. This is consistent with the notion that HERC2 ubiquitination of CP110 precedes the linear ubiquitination of the CP110 ubiquitin by the LUBAC system. We have discussed this in the Discussion on p. 10, lines 319-326. We have further attempted to address whether HERC2 knockdown prevents PRPF8 from interacting with CP110 (which we would predict in this model); however, multiple experiments were inconclusive because the PRPF8 antibodies were of insufficient quality to test this hypothesis. While PRPF8 was identified in interactions with ubiquitinated CP110 (Shen et al., J. Cell Biol., 2021), those studies were done using transfected proteins. Accordingly, a full

elucidation of the complex interplay between these ubiquitin ligases and CP110 will require significant additional efforts in the coming years.

Major points:

1. The impact of EHD1 on centriolar satellite distribution and PCM1 intensity is interesting but is somewhat preliminary and based largely on correlations. It is unclear how EHD1 controls motility of centriolar satellites and whether the observed change in centriolar satellite distribution is the cause or consequence of EHD1 depletion. PCM1 intensity should be measured separately in ciliated and non-ciliated cells in mock- and EHD1-depleted cells. I realise that in EHD1-depleted cells there will be much fewer cilia, and the ones with cilia are likely to represent cells with residual EHD1 activity, but these could still serve as positive controls.

We thank the reviewer for this suggestion and now provide new data showing PCM1 intensity proximal to the centrosome in both ciliated and non-ciliated Mock and EHD1-depleted cells. This data now appears in Revised Fig. 2Q.

2. Co-depletion of CP110 with HERC2 clearly shows rescue of ciliogenesis. However, from the image cilia of co-depleted cells appear shorter and perhaps bendier. Is that so? Also, there is a notable change in overall acetylation levels of tubulin in cells. A closer examination of these cells could provide additional insight.

We thank the reviewer for these interesting observations; accordingly, we have measured cilia length in the Mock depleted, HERC2 depleted, CP110 depleted and co-depleted cells and find that either CP110 depletion or CP110 depletion together with HERC2 depletion lead not only to more ciliated cells, but also to longer cilia (see new Fig. 6G). Interestingly, the HERC2 and CP110 double knock-down cells had slightly shorter cilia than the CP110 knock-down cells. With regard to the degree of bending of the cilia, this is far more difficult to measure. However, by careful examination of multiple fields of cells we are not convinced that there is a difference in the overall linearity of these structures. Regarding the overall levels of acetylated tubulin, it is difficult to measure since the "background" of non-ciliar acetylated tubulin observed relies on the plane of focus, and in the absence of cilia it is difficult to ensure that we are on the equivalent focal plane. However, this is a very significant question and based on some preliminary experimentation we speculate that EHD1 knock-down might impact microtubules, thus the reviewer has highlighted a crucial area of focus for future studies.

3. Fig 1H, 2A: IP efficiency should also be demonstrated with CP110 antibody. Densitometry analysis could then be normalised against total immunoprecipitated CP110.

We apologize if our original submission was not clear—Revised Fig. 1J (formerly 1H), 2A and 3B do represent IP of CP110 which is first immunoblotted with anti-ubiquitin prior to stripping and reblotting with anti-CP110 antibodies. Accordingly, levels of ubiquitinated CP110 are normalized to the amount of CP110 pulled down by IP, as recommended by the reviewer. The description of the IP and immunoblotting is described in each relevant figure legend.

4. Fig 3A: HERC2 blot is very poor, specificity is unclear. Cullin 3 is weakly depleted.

HERC2 is a very large protein that has proved difficult to resolve and detect by immunoblotting in the literature. It is often detected as a smeared band on gels in the literature. In comparison with other publications, our HERC2 is relatively well-detected. Nonetheless, we have scaled up our experiments to

try and obtain a gel with higher signal-to-noise ratio, now shown in Revised Fig. 3A. With regard to Cullin3, our depletion ranges from 60-85% depending on the experiment and linear range of the densitometry. While depletion levels of closer to 100% would be optimal, we are unable to significantly improve knockdown levels beyond what we show.

5 Fig 3B: Again, a blot to show efficacy of CP110 immunoprecipitation is lacking. Quantitative analysis by densitometry is needed.

As noted above, we apologize if our original submission was not clear—Former Fig. 1H (now 1J), 2A and 3B do represent IP of CP110 which is first immunoblotted with anti-ubiquitin prior to stripping and reblotting with anti-CP110 antibodies. Accordingly, levels of ubiquitinated CP110 are normalized to the amount of CP110 pulled down by IP, as recommended by the reviewer. The description of the IP and immunoblotting is described in each relevant figure legend.

6. Fig 5A: the blot demonstrating lack of interaction between CP110 and HERC2 in absence of EHD1 is really important. The current blot does not provide high enough quality proof due to weak HERC2 signal and the unfortunate shading on top of blot in the EHD1 siRNA lanes.

We agree with the reviewer about the significance of this finding. However, as noted above, HERC2 is a very large protein that has proved difficult to resolve and detect by immunoblotting in the literature. Moreover, when immunoprecipitated with CP110, the band is relatively modest, but clearly observed. In EHD1 knockdown cells, the HERC2 band is always undetectable. We have nonetheless carried out this experiment again, scaling up the cells used, and now provide a somewhat clearer/brighter gel showing the same effect in revised Fig. 5A.

7. Fig 4F-M: images are very pixelated

We apologize for the pixelation; this is a result of enlarging the original images (in B, E, H and K) to clearly highlight the loss (or not) of CP110 from the mother centriole. The images are only meant to supplement B, E, H and K and better showcase the mother centrioles.

8. Figures generally include statistical analysis, although the actual test carried out should be mentioned in figure legends. For measuring intensities in images (i.e. PCM1, HERC2) or mean particle speeds, it would be more informative to provide dot/box plots of actual datapoints and compare distributions rather than a single average value per experiment.

We have now included the methods for statistical analysis both in each individual legend as well as the Materials and Methods. We have also modified the type of graphs used, where appropriate, to include all the data points as recommended.

Minor points:

Lack of line and page numbers made it difficult to reference text.

We apologize for this oversight and have now included line and page numbers.

1. I am surprised that the greatest increase in HERC2 levels at centrosomes occurs within 10 minutes of serum withdrawal. How would the authors explain this very fast response?

This is an excellent question. Although we do not know the answer, we rationalize that since CP110 removal needs to occur prior to fusion of distal appendage vesicles and formation of the ciliary vesicle at the early stages of ciliogenesis, centriolar satellite delivery of HERC2 to the mother centriole for CP110 ubiquitination must occur relatively rapidly. This is an interesting follow-up question for future study.

2. According to 1D, over 70% of cells grow a primary cilia within 4 hours of serum starvation. This again seems higher than values reported in the literatures. By the same measure, in Fig 6E mock transfected cells show only 50% ciliation.

We agree with the reviewer that 50-60% ciliation is commonly observed in the literature and in our studies as well. However, we find that the range can vary between 40-75% using our RPE-1 cells depending on a given set of experiments. We do not know whether this reflects the status of the cells in culture or other factors, but there is biological variation in the degree of ciliation observed. Importantly, however, is the degree to which this "baseline ciliation" is affected upon various treatments or knockdowns, and our data is very consistent for MG132 treatment and knockdown of EHD1, HERC2, PCM1, etc.

3. "they might assemble in an immune complex" Shouldn't this be 'protein complex'?

We thank the reviewer for this point and have modified the text accordingly. (p.6, line 203)

4. HERC2 has been shown to positively regulate ciliogenesis (Quarantotti et al., EMBO J, 2019).

We thank the reviewer for this reminder and have modified the text accordingly. (p.9, line 309-312)

1st Revision - Editorial Decision 29th Mar 2023

Dear Steve,

Thank you once more for the submission of your revised manuscript to EMBO reports. As you know, we have meanwhile received the report from referee 3 who was asked to assess the revised manuscript.

As you will see, the referee is now very positive about the study and requests only minor changes to clarify text and conclusions.

Browsing through the manuscript myself, I noticed a few editorial things that we need before we can proceed with the official acceptance of your study.

- Please reduce the number of keywords to 5.

- Regarding the Author Contributions, we now use CRediT to specify the contributions of each author in the journal submission system. CRediT replaces the author contribution section, which therefore needs to be removed from the manuscript text. You can use the free text box in our system if you wish to provide more detailed descriptions. See also guide to authors https://www.embopress.org/page/journal/14693178/authorguide#authorshipguidelines.

- Please add callouts to the panels of the EV figures (e.g., Fig EV2A-H)

- Please ZIP each movie with its legend (txt file) and upload the ZIPed file.

- Please correct the heading "Expanded View Figure" to Expanded View Figure Legends

- The scale bars should be thicker and the scale number should only be in the figure legend and removed from the figure itself. Please also add a scale bar for Fig 1E.

- I attach to this email a related manuscript file with comments by our data editors. Please address all comments and upload a revised file with tracked changes with your final manuscript submission.

- Finally, EMBO reports papers are accompanied online by A) a short (1-2 sentences) summary of the findings and their significance, B) 2-3 bullet points highlighting key results and C) a synopsis image that is 550x300-600 pixels large (width x height) in PNG for JPG format. You can either show a model or key data in the synopsis image. Please note that the size is rather small and that text needs to be readable at the final size. Please send us this information along with the revised manuscript.

- In case you will be using BioRender to generate the synopsis image, please insert text "Created with BioRender' in small letters (7.5ish) in the synopsis image itself. Please also send us a copy of your BioRender license for Figure 7. Thank you.

We look forward to seeing a final version of your manuscript as soon as possible.

With kind regards,

Martina

Martina Rembold, PhD
Senior Editor
EMBO reports

****

Referee #3:

The authors have substantially improved their manuscript and addressed my comments satisfactorily. I have only one minor point to make.

1. Line 123: These data collectively suggest that CP110 ubiquitination...

This conclusion does not reflect the data the authors refer to (Figs 1 and EV1). It is not shown that the impact of MG132 on ciliation is directly linked to CP110 removal/ubiquitination. This is simply a correlation, so the sentence should be adjusted accordingly, especially because MG132 treatment has pleiotropic effects.

Point-by-Point Response to the Reviewer's Comment:

The authors have substantially improved their manuscript and addressed my comments satisfactorily. I have only one minor point to make.

1. Line 123: These data collectively suggest that CP110 ubiquitination...

This conclusion does not reflect the data the authors refer to (Figs 1 and EV1). It is not shown that the impact of MG132 on ciliation is directly linked to CP110 removal/ubiquitination. This is simply a correlation, so the sentence should be adjusted accordingly, especially because MG132 treatment has pleiotropic effects.

We thank the reviewer for her/his support of our revised manuscript. We have now adjusted the conclusion to better reflect our actual findings and separate between these observed findings and what we consider to be a potential or likely interpretation of the findings. The revision is highlighted in yellow.

Prof. Steve Caplan
University of Nebraska Medical Center
Biochemistry and Molecular Biology
5870 Nebraska Medical Center
Duram Research Center 7013
Omaha, Nebraska 68198-5870
United States

Dear Steve,

I am very pleased to accept your manuscript for publication in the next available issue of EMBO reports. Thank you for your contribution to our journal.

At the end of this email I include important information about how to proceed. Please ensure that you take the time to read the information and complete and return the necessary forms to allow us to publish your manuscript as quickly as possible.

As part of the EMBO publication's Transparent Editorial Process, EMBO reports publishes online a Review Process File to accompany accepted manuscripts. As you are aware, this File will be published in conjunction with your paper and will include the referee reports, your point-by-point response and all pertinent correspondence relating to the manuscript.

If you do NOT want this File to be published, please inform the editorial office within 2 days, if you have not done so already, otherwise the File will be published by default [contact: emboreports@embo.org]. If you do opt out, the Review Process File link will point to the following statement: "No Review Process File is available with this article, as the authors have chosen not to make the review process public in this case."

Thank you again for your contribution to EMBO reports and congratulations on a successful publication. Please consider us again in the future for your most exciting work.

Kind regards,

Martina

Martina Rembold, PhD
Senior Editor
EMBO reports

****

THINGS TO DO NOW:

Please note that you will be contacted by Wiley Author Services to complete licensing and payment information. The required 'Page Charges Authorization Form' is available here: https://www.embopress.org/pb-assets/embo-site/er_apc.pdf - please download and complete the form and return to embopressproduction@wiley.com

EMBO Press participates in many Publish and Read agreements that allow authors to publish Open Access with reduced/no publication charges. Check your eligibility: https://authorservices.wiley.com/author-resources/Journal-Authors/open-access/affiliation-policies-payments/index.html

You will receive proofs by e-mail approximately 2-3 weeks after all relevant files have been sent to our Production Office; you should return your corrections within 2 days of receiving the proofs.

Please inform us if there is likely to be any difficulty in reaching you at the above address at that time. Failure to meet our deadlines may result in a delay of publication, or publication without your corrections.

All further communications concerning your paper should quote reference number EMBOR-2022-56317V3 and be addressed to emboreports@wiley.com.

Should you be planning a Press Release on your article, please get in contact with emboreports@wiley.com as early as possible, in order to coordinate publication and release dates.

EMBO Press Author Checklist

Corresponding Author Name: Steve Caplan	
Journal Submitted to: EMBO Reports	
Manuscript Number: EMBOR-2022-56317-T	

USEFUL LINKS FOR COMPLETING THIS FORM
The EMBO Journal - Author Guidelines
EMBO Reports - Author Guidelines
Molecular Systems Biology - Author Guidelines
EMBO Molecular Medicine - Author Guidelines

Reporting Checklist for Life Science Articles (updated January 2022)
This checklist is adapted from Materials Design Analysis Reporting (MDAR) Checklist for Authors. MDAR establishes a minimum set of requirements in transparent reporting in the life sciences (see Statement of Task: 10.31222/osf.io/9sm4x). Please follow the journal's guidelines in preparing your manuscript.
Please note that a copy of this checklist will be published alongside your article.

Abridged guidelines for figures
1. Data
The data shown in figures should satisfy the following conditions:
- the data were obtained and processed according to the field's best practice and are presented to reflect the results of the experiments in an accurate and unbiased manner.
- ideally, figure panels should include only measurements that are directly comparable to each other and obtained with the same assay.
- plots include clearly labeled error bars for independent experiments and sample sizes. Unless justified, error bars should not be shown for technical replicates.
- if n<5, the individual data points from each experiment should be plotted. Any statistical test employed should be justified.
- Source Data should be included to report the data underlying figures according to the guidelines set out in the authorship guidelines on Data Presentation.

2. Captions
Each figure caption should contain the following information, for each panel where they are relevant:
- a specification of the experimental system investigated (eg cell line, species name).
- the assay(s) and method(s) used to carry out the reported observations and measurements.
- an explicit mention of the biological and chemical entity(ies) that are being measured.
- an explicit mention of the biological and chemical entity(ies) that are altered/varied/perturbed in a controlled manner.
- the exact sample size (n) for each experimental group/condition, given as a number, not a range;
- a description of the sample collection allowing the reader to understand whether the samples represent technical or biological replicates (including how many animals, litters, cultures, etc.).
- a statement of how many times the experiment shown was independently replicated in the laboratory.
- definitions of statistical methods and measures:
  - common tests, such as t-test (please specify whether paired vs. unpaired), simple χ2 tests, Wilcoxon and Mann-Whitney tests, can be unambiguously identified by name only, but more complex techniques should be described in the methods section;
  - are tests one-sided or two-sided?
  - are there adjustments for multiple comparisons?
  - exact statistical test results, e.g., P values = x but not P values < x;
  - definition of 'center values' as median or average;
  - definition of error bars as s.d. or s.e.m.

Please complete ALL of the questions below.
Select "Not Applicable" only when the requested information is not relevant for your study.

Materials

Newly Created Materials	Information included in the manuscript?	In which section is the information available? (Reagents and Tools Table, Materials and Methods, Figures, Data Availability Section)
New materials and reagents need to be available; do any restrictions apply?	Not Applicable	

Antibodies	Information included in the manuscript?	In which section is the information available? (Reagents and Tools Table, Materials and Methods, Figures, Data Availability Section)
For **antibodies** provide the following information:<br>- Commercial antibodies: RRID (if possible) or supplier name, catalogue number and or/clone number<br>- Non-commercial: RRID or citation	Yes	In Materials and Methods, Antiboies and Reagents section

DNA and RNA sequences	Information included in the manuscript?	In which section is the information available? (Reagents and Tools Table, Materials and Methods, Figures, Data Availability Section)
**Short novel DNA or RNA including primers, probes**: provide the sequences.	Yes	Materials and Methods Cell culture and treatments section

Cell materials	Information included in the manuscript?	In which section is the information available? (Reagents and Tools Table, Materials and Methods, Figures, Data Availability Section)
**Cell lines:** Provide species information, strain. Provide accession number in repository **OR** supplier name, catalog number, clone number, and/**OR** RRID.	Yes	Materials and Methods, Cell culture and treatments
**Primary cultures:** Provide species, strain, sex of origin, genetic modification status.	Not Applicable	
Report if the cell lines were recently **authenticated** (e.g., by STR profiling) and tested for mycoplasma contamination.	Yes	Materials and Methods, Cell culture and treatments

Experimental animals	Information included in the manuscript?	In which section is the information available? (Reagents and Tools Table, Materials and Methods, Figures, Data Availability Section)
**Laboratory animals or Model organisms:** Provide species, strain, sex, age, genetic modification status. Provide accession number in repository **OR** supplier name, catalog number, clone number, **OR** RRID.	Not Applicable	
**Animal observed in or captured from the field:** Provide species, sex, and age where possible.	Not Applicable	
Please detail **housing and husbandry conditions**.	Not Applicable	

Plants and microbes	Information included in the manuscript?	In which section is the information available? (Reagents and Tools Table, Materials and Methods, Figures, Data Availability Section)
**Plants:** provide species and strain, ecotype and cultivar where relevant, unique accession number if available, and source (including location for collected wild specimens).	Not Applicable	
**Microbes:** provide species and strain, unique accession number if available, and source.	Not Applicable	

Human research participants	Information included in the manuscript?	In which section is the information available? (Reagents and Tools Table, Materials and Methods, Figures, Data Availability Section)
If collected and within the bounds of privacy constraints report on age, sex and gender or ethnicity for all study participants.	Not Applicable	

Core facilities	Information included in the manuscript?	In which section is the information available? (Reagents and Tools Table, Materials and Methods, Figures, Data Availability Section)
If your work benefited from core facilities, was their service mentioned in the acknowledgments section?	Yes	Mentioned in Materials and Methods and Acknowledgments

Design

Study protocol	Information included in the manuscript?	In which section is the information available? (Reagents and Tools Table, Materials and Methods, Figures, Data Availability Section)
If study protocol has been **pre-registered, provide DOI in the manuscript**. For clinical trials, provide the trial registration number **OR** cite DOI.	Not Applicable	
Report the **clinical trial registration number** (at ClinicalTrials.gov or equivalent), where applicable.	Not Applicable	

Laboratory protocol	Information included in the manuscript?	In which section is the information available? (Reagents and Tools Table, Materials and Methods, Figures, Data Availability Section)
Provide DOI OR other citation details if **external detailed step-by-step protocols** are available.	Not Applicable	

Experimental study design and statistics	Information included in the manuscript?	In which section is the information available? (Reagents and Tools Table, Materials and Methods, Figures, Data Availability Section)
Include a statement about **sample size** estimate even if no statistical methods were used.	Yes	Sample size is given in each figure legend
Were any steps taken to minimize the effects of subjective bias when allocating animals/samples to treatment (e.g. **randomization procedure**)? If yes, have they been described?	Not Applicable	
Include a statement about **blinding** even if no blinding was done.	Yes	Materials and Methods, Statistical analysis section
Describe **inclusion/exclusion criteria** if samples or animals were excluded from the analysis. Were the criteria pre-established?<br><br>If sample or data points were omitted from analysis, report if this was due to attrition or intentional exclusion and provide justification.	Not Applicable	
For every figure, are **statistical tests** justified as appropriate? Do the data meet the assumptions of the tests (e.g., normal distribution)? Describe any methods used to assess it. Is there an estimate of variation within each group of data? Is the variance similar between the groups that are being statistically compared?	Yes	In the figure legends of every figure and sub-figure, the relevant normality tests that were used have been detailed, and it has been noted whether the data meet the assumption for normality. Accordingly, the appropriate statistical analyses were used (parametric or non-parametric) and this is detailed in each figure legend. A general expalnation of statistical analysis is provided in the Materials and Methods (Statistical Analysis Section)

Sample definition and in-laboratory replication	Information included in the manuscript?	In which section is the information available? (Reagents and Tools Table, Materials and Methods, Figures, Data Availability Section)
In the figure legends: state number of times the experiment was **replicated** in laboratory.	Yes	In figure legends
In the figure legends: define whether data describe **technical or biological replicates**.	Yes	In figure legends

Ethics

Ethics	Information included in the manuscript?	In which section is the information available? (Reagents and Tools Table, Materials and Methods, Figures, Data Availability Section)
Studies involving **human participants**: State details of **authority granting ethics approval** (IRB or equivalent committee(s), provide reference number for approval.	Not Applicable	
Studies involving **human participants**: Include a statement confirming that **informed consent** was obtained from all subjects and that the experiments conformed to the principles set out in the WMA Declaration of Helsinki and the Department of Health and Human Services Belmont Report.	Not Applicable	
Studies involving **human participants:** For publication of **patient photos**, include a statement confirming that consent to publish was obtained.	Not Applicable	
Studies involving experimental **animals**: State details of **authority granting ethics approval** (IRB or equivalent committee(s), provide reference number for approval. Include a statement of compliance with ethical regulations.	Not Applicable	
Studies involving **specimen and field samples:** State if relevant **permits** obtained, provide details of authority approving study; if none were required, explain why.	Not Applicable	

Dual Use Research of Concern (DURC)	Information included in the manuscript?	In which section is the information available? (Reagents and Tools Table, Materials and Methods, Figures, Data Availability Section)
Could your study fall under dual use research restrictions? Please check biosecurity documents and list of **select agents and toxins** (CDC): https://www.selectagents.gov/sat/list.htm	Not Applicable	
If you used a select agent, is the security level of the lab appropriate and reported in the manuscript?	Not Applicable	
If a study is subject to dual use research of concern regulations, is the name of the **authority granting approval and reference number** for the regulatory approval provided in the manuscript?	Not Applicable	

Reporting

The MDAR framework recommends adoption of discipline-specific guidelines, established and endorsed through community initiatives. Journals have their own policy about requiring specific guidelines and recommendations to complement MDAR.

Adherence to community standards	Information included in the manuscript?	In which section is the information available? (Reagents and Tools Table, Materials and Methods, Figures, Data Availability Section)
State if relevant guidelines or checklists (e.g., **ICMJE, MIBBI, ARRIVE, PRISMA**) have been followed or provided.	Not Applicable	
For **tumor marker prognostic studies,** we recommend that you follow the **REMARK** reporting guidelines (see link list at top right). See author guidelines, under 'Reporting Guidelines'. Please confirm you have followed these guidelines.	Not Applicable	
For **phase II and III randomized controlled trials**, please refer to the **CONSORT** flow diagram (see link list at top right) and submit the CONSORT checklist (see link list at top right) with your submission. See author guidelines, under 'Reporting Guidelines'. Please confirm you have submitted this list.	Not Applicable	

Data Availability

Data availability	Information included in the manuscript?	In which section is the information available? (Reagents and Tools Table, Materials and Methods, Figures, Data Availability Section)
Have **primary datasets** been deposited according to the journal's guidelines (see 'Data Deposition' section) and the respective accession numbers provided in the Data Availability Section?	Not Applicable	
Were **human clinical and genomic datasets** deposited in a public access-controlled repository in accordance to ethical obligations to the patients and to the applicable consent agreement?	Not Applicable	
Are **computational models** that are central and integral to a study available without restrictions in a machine-readable form? Were the relevant accession numbers or links provided?	Not Applicable	
If publicly available data were reused, provide the respective **data citations in the reference list**.	Not Applicable	
